# Plant-Derived Senotherapeutics in Cellular Senescence: A Scoping Review of Preclinical Evidence, Mechanistic Pathways, and Metabolomic-Guided Discovery

**DOI:** 10.3390/ijms27167181

**Published:** 2026-08-11

**Authors:** Nor Muhammad Hilmi Hussin, Ahmed Mediani, Normala Abd Latip, Michael Fenech, Rahma Micho Widyanto, Razinah Sharif

**Affiliations:** 1 Centre for Healthy Ageing and Wellness (H-CARE), Faculty of Health Sciences, Universiti Kebangsaan Malaysia, Kuala Lumpur 50300, Malaysia; p153456@siswa.ukm.edu.my; 2 Institute of Systems Biology (INBIOSIS), Universiti Kebangsaan Malaysia, Bangi 43600, Malaysia; 3 Integrative Pharmacogenomics Institute (iPROMISE), Universiti Teknologi MARA (UiTM) Selangor, Puncak Alam 42300, Malaysia; drnormala6351@uitm.edu.my; 4 Health and Biomedical Innovation, UniSA Clinical and Health Sciences, University of South Australia, Adelaide 5000, Australia; mf.ghf@outlook.com; 5 Faculty of Health Sciences, Universitas Brawijaya, Malang 65151, Indonesia; micho@ub.ac.id

**Keywords:** cellular senescence, senolytic, senomorphic, bioactive compound, aging, plant-derived compounds, SASP, metabolomics

## Abstract

Senotherapeutic agents targeting senescent cell (SnC) accumulation represent a promising frontier in aging research. These agents encompass senolytics that selectively eliminate accumulated SnCs and senomorphics that suppress the pathological persistence of the senescence-associated secretory phenotype (SASP). Concerns regarding off-target effects of synthetic senolytics have intensified interest in plant-derived alternatives that offer multitargeted mechanisms and favorable safety profiles. This scoping review was conducted following Joanna Briggs Institute guidelines and PRISMA-ScR, mapped preclinical evidence on plant-derived senotherapeutics published between 2015 and 2025 across PubMed, Scopus, Web of Science, Wiley Library and Google Scholar. Of 1355 identified articles, 111 studies met inclusion criteria. Most characterized compound classes included flavonoids, non-flavonoid polyphenols and stilbenes, terpenoids and alkaloids, and combination and complex plant extracts. Mechanistically, BCL-2/BCL-XL apoptosis, PI3K/AKT/mTOR and p53/p21/p16^INK4a^ modulation emerged as senolytic mechanisms, while NF-κB-mediated SASP suppression predominated among senomorphic agents. Ginkgetin-mediated cyclic GMP-AMP-synthase–stimulator of interferon genes (cGAS-STING) inhibition was identified as a mechanistically novel target within natural senotherapy. Metabolomics demonstrated dual utility in guiding compound discovery from complex plant matrices (e.g., phenolamides from *Allium hookeri*) and mechanistic validation by characterizing senescence-associated metabolic remodeling, including retinoic acid metabolism restoration, lipotoxic metabolites attenuation, tricarboxylic acid (TCA) cycle, and choline-betaine-TCA cascade regulation. However, challenges in pharmacokinetic optimization, methodological heterogeneity in senescence induction and biomarker panels persist. Plant-derived senotherapy characterized through metabolomics-guided pipelines provides a compelling foundation for their progression toward clinical validation and functional food applications as accessible interventions for healthy aging and age-related disease management.

## 1. Introduction

Recent advances in geroscience have defined aging as a biological process underpinned by 12 interconnected hallmarks, among which the progressive accumulation of senescent cells (SnCs) has emerged as a central pillar of aging pathophysiology [1]. Cellular senescence is an irreversible cell cycle arrest state mediated primarily through p53-p21 and p16^INK4a^-Rb signaling cascades. Senescence is induced through distinct routes, including replicative senescence driven by progressive telomere attrition, stress-induced premature senescence (SIPS) triggered by oxidative stress, DNA damage or other cellular insults, and oncogene-induced senescence (OIS) [1]. SnCs undergo profound phenotypic changes, including increased lysosomal content (senescence-associated β-galactosidase (SA-β-gal)) and notably, the acquisition of the senescence-associated secretory phenotype (SASP). The SASP is a potent cell-non-autonomous secretome enriched in pro-inflammatory cytokines, chemokines, matrix metalloproteinases, and growth factors that collectively propagate local and systemic inflammation [2]. While acute senescence serves as a protective mechanism against proliferation of damaged cells, the sustained accumulation of SnCs and its associated SASP with advancing age promotes a self-perpetuating cycle of low-grade inflammation, termed inflammaging [1,2]. This process progressively impairs tissue regenerative capacity and drives the onset of multiple age-related morbidity, including cardiovascular disease, neurodegeneration, metabolic disorders and cancer.

In response, senotherapeutics have emerged as a promising strategy for targeted intervention against SnC populations. This therapeutic approach encompasses two mechanistically discrete agent classes classified as senolytic, which selectively eliminate SnCs by disrupting senescent cell anti-apoptotic pathways (SCAPs) and senomorphics, which suppress the SASP without eliminating the cells themselves [3,4,5]. The antioxidant and anti-inflammatory actions of many bioactive compounds are relevant to senescence biology, as oxidative stress and chronic inflammation are major drivers of SnC and SASP amplification, and their attenuation can meaningfully reduce the burden that promotes the senescent phenotype [6,7]. Nonetheless, true senomorphic agents do not merely dampen secondary oxidative and inflammatory stress; rather, they require direct engagement with core SASP-driven upstream transcriptional and signaling mechanisms. Both classes have demonstrated efficacy to extend both healthspan and lifespan in preclinical models by attenuating the progression of age-related tissue dysfunction while preventing the transition of healthy cells toward senescence. Since the landmark discovery of the first senolytic by Zhu et al. (2015) [8], significant challenges have arisen regarding clinical translation. The BCL-2 and BCL-XL inhibitor navitoclax (ABT-263) caused dose-limiting thrombocytopenia in human trials owing to on-target elimination of platelets [9,10] and cardiac glycoside analogues were associated with adverse effects, including cardiac arrhythmias, blood pressure disturbance, and gastrointestinal and neurological toxicity [11]. Henceforth, these limitations have motivated intensive investigation into plant-derived alternatives that may offer safe and more favorable tissue-selectivity profiles.

Plant-derived compounds from medicinal plants, functional foods, and dietary sources frequently engage with multiple conserved molecular targets, conferring the intrinsic polypharmacology [12,13]. This allows several aging-related signaling mechanisms to be modulated in parallel, potentially achieving senotherapeutic efficacy through mechanistically diverse and non-hematotoxic routes [13,14]. Owing to their evolution within biological systems, these compounds often exhibit favorable safety profiles and access unique chemical spaces characterized by structural diversity underrepresented in synthetic compounds. The flavanol quercetin was among the first natural compounds validated as a senolytic component in a dasatinib and quercetin (D + Q) combination, a milestone that catalyzed research for additional natural senotherapeutics. Emerging evidence suggests that structurally diverse plant compounds attenuate senescence induction and SASP-mediated inflammatory cascades across in vitro and in vivo models and in preliminary human investigations [13,15]. Recent examples include procyanidin C1 (PCC1), which exhibits dual senolytic and senomorphic properties through modulation of the BCL-2 family of proteins, and ROS [16] and apigenin, which suppresses SASP via ATM–p38 MAPK signaling [17]. Despite this promise, the chemical complexity of botanical matrices creates a fundamental discovery challenge. When senotherapeutic activity is observed in a crude plant extract or polyherbal formulation, identifying which constituent molecules are responsible and through which mechanistic pathways they act requires analytical tools capable of resolving the full chemical and metabolic complexity of the biological system. Existing reviews have predominantly focused on compound mechanism relationships for isolated pure compounds, leaving the senotherapeutic potential of crude plant extracts and poly-extract formulations in which activity may arise from synergistic activity among multiple co-occurring compounds systematically undercharacterized.

Metabolomics addresses this challenge by providing comprehensive qualitative and quantitative profiling of small-molecule compounds within biological systems, including carbohydrates, amino acids, lipids, organic acids, polyphenols, hormones, and signaling molecules [18]. Over the past few years, it has become a powerful platform for identifying the variations affecting various metabolic pathways, discovery of biomarkers, and investigation of drug efficacy and toxicity and novel therapeutic targets, opening up new routes for therapeutic approaches, especially for age-related diseases [18]. Multiple studies have applied metabolomics to medicinal plant research [19]. Markedly, metabolomics has revolutionized natural product drug discovery by profiling a range of bioactive compounds within complex plant matrices, dereplication of known compounds from novel botanical sources, and direct mapping of the metabolic pathway alterations that define the cellular response to biological effects [18,19]. When integrated with molecular networking and computational annotation, this discovery pipeline accelerates the transition from crude extract to validated senotherapeutic or anti-aging lead compounds and elucidating synergistic interactions within complex combinations and botanicals [19]. Yet, the systematic application of metabolomics to the discovery and validation of plant-derived senotherapeutics remains nascent. Comprehensive evidence mapping through the lens of metabolomics-guided discovery is therefore needed for compound discovery, mechanistic pathway characterization or bioavailability assessment across the full range of plant senotherapeutics studied. Moreover, extant evidence is fragmented across methodologically heterogenous study designs, encompassing in vitro cell models, senescence induction protocols, in vivo animal studies, mechanistic investigations, and narrative reviews. Such heterogeneity renders quantitative synthesis particularly systematic and meta-analysis methodologies challenging to apply at present. According to the methodological guidance of the Joanna Briggs Institute (JBI) and the PRISMA Extension for Scoping Reviews (PRISMA-ScR), scoping reviews are particularly well-suited for mapping the breadth of available evidence, identifying research gaps, and clarifying key concepts in emerging and heterogeneous fields. Hence, a scoping review represents the most appropriate approach to characterize how plant-derived compounds and extracts exert senotherapeutic activity across diverse cell types, senescence inducers, and model systems, and to inform future research priorities.

Accordingly, our review aims to synthesize the available preclinical evidence on plant-derived compounds with documented senolytic and/or senomorphic activities. Specifically, we sought to (i) identify and characterize natural senotherapeutic compounds and extracts; (ii) elucidate the molecular signaling pathways through which these compounds exert effects, including their interactions with established aging-related pathways across diverse cell types and models; (iii) characterize the current contribution of and substantive gaps in metabolomics-guided discovery and mechanistic validation workflows within the natural senotherapy pipeline; and (iv) evaluate their preclinical efficacy and identify translational barriers. The review seeks to generate a structured evidence base that informs the rational development of plant-derived senotherapeutics as a safe and effective intervention targeting cellular senescence and healthy aging.

## 2. Materials and Methods

**Figure 1:**
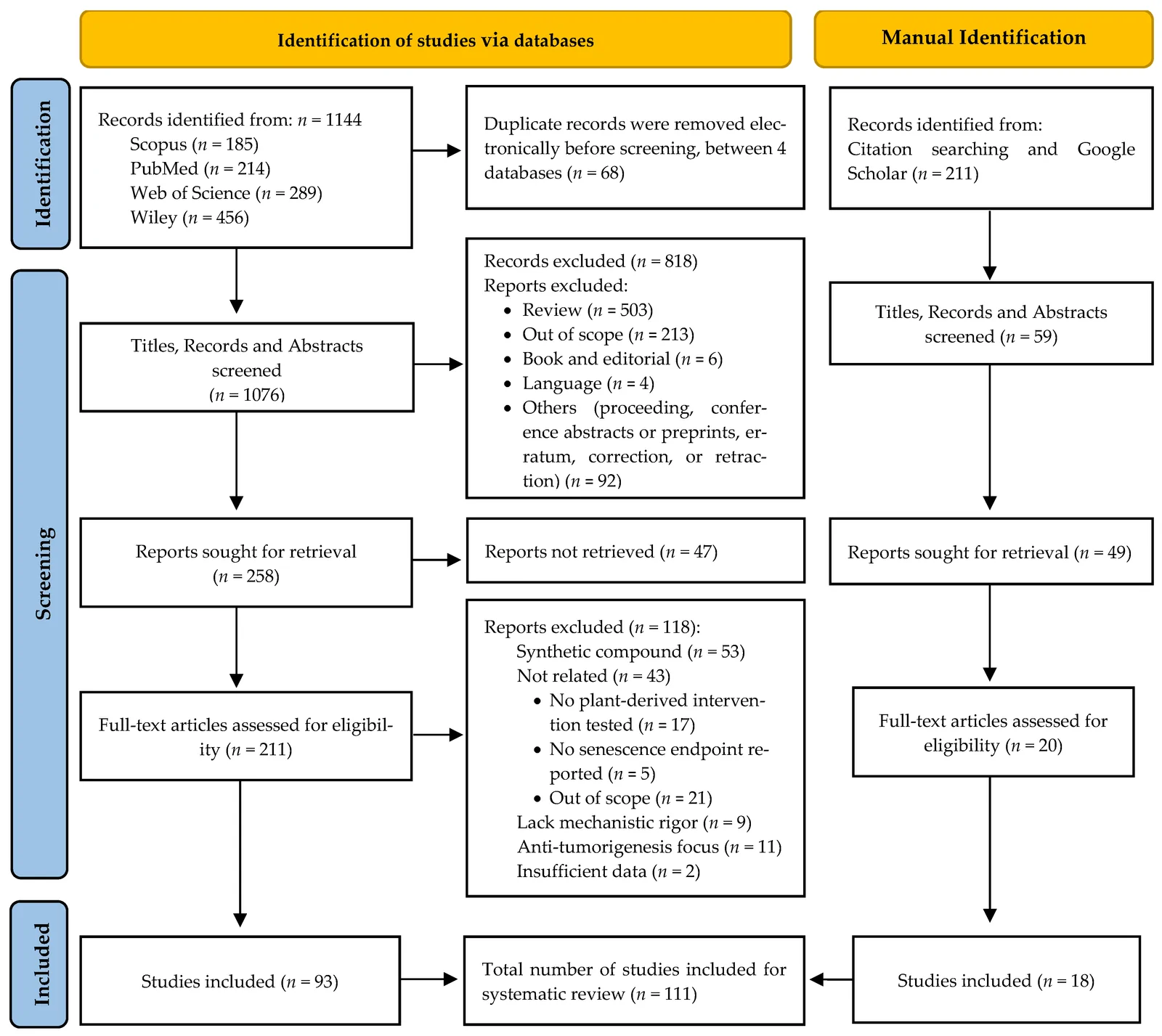
PRISMA flow diagram illustrating the identification, screening, and inclusion process for the scoping review.

This scoping review was conducted in accordance with the Joanna Briggs Institute (JBI) [20] framework and the Preferred Reporting Items for Systematic Reviews and Meta-Analyses Extension for Scoping Reviews (PRISMA-ScR) guidelines [21]. A formal protocol was not prospectively registered. Instead, the review methodology was developed and agreed upon by all authors prior to conducting the searches. The completed PRISMA-ScR checklist is provided as Supplementary Material Table S1, and the PRISMA flow diagram is presented in Figure 1.

### 2.1. Identifying the Research Questions

The purpose of this study is to present a comprehensive synthesis of available preclinical evidence on plant-derived compounds with documented senolytic and/or senomorphic activities and to characterize how they exert senotherapeutic effects. The research questions were identified to direct the review and determine the relevant studies. Thus, this scoping review was intended to answer the following research questions:

1. Which plant-derived compounds and extracts have been identified as natural senotherapeutics, and what compound classes do they represent?
2. What molecular signaling pathways are modulated by these plant-derived compounds?
3. What is the preclinical therapeutic efficacy and translational barriers of these compounds, including bioavailability, optimal dosing, and long-term safety?
4. What is the current application of analytical workflows in the discovery of natural senotherapeutics?

### 2.2. Identifying Relevant Studies

#### 2.2.1. Search Terms

The search strategy was restricted to original research articles published between January 2015 and December 2025, capturing most current knowledge and research advancements in the field while including the period following senolytic discoveries. All database searches were executed in January 2026. The Population, Concept, and Context (PCC) framework guided this stage (Table 1).

A combination of PubMed’s Medical Subject Headings (MeSH) terms and free-text keywords related to cellular senescence, senotherapeutics, natural products, biological effects, and related pathways were carefully analyzed by two reviewers (N.M.H.H. and R.S.). The complete database-specific search strings and the number of records retrieved from each source are provided in Supplementary Table S3. Search terms consisted of various combinations of keywords utilizing Boolean operators (AND, OR) to enhance the precision and relevance of the search results. Some examples of search terms included the following:

- (“natural product” OR “herbal medicine” OR “plant extract” OR “polyherbal” OR “medicinal plant” OR “phytochemicals” OR “polyphenol” OR “flavonoid*”);
- AND (“cellular senescence” OR “senolytic” OR “senomorphic” OR “senescence-associated secretory phenotype” OR “sasp”);
- AND (“mechanism” OR “pathway” OR “mode of action” OR “inhibition” OR “activation”).

Eligible studies included in vitro and in vivo studies published in English-language-only journals. The articles provided sufficient data on efficacy and underlying biological mechanisms of natural senotherapeutics by assessing senescence biomarkers, including, but not limited to, SA-β-gal activity, cell cycle regulators p16 and p21, SASP factors, markers of SnCs viability, and apoptosis. Studies were excluded according to the following criteria: (1) non-primary research (reviews, meta-analyses, editorials, commentaries, book chapters, and conference abstracts); (2) focus exclusively on synthetic compounds without investigation of natural product counterparts or derivatives; (3) insufficient methodological data (sample sizes, replicate numbers, or statistical tests) or without clear outcomes (senescence findings reported only as non-quantified narrative statement (e.g., ‘Senescence markers were reduced’) without supporting verification); (4) absent senescence biomarker or mechanism assessment; (5) inaccessible as full text.

#### 2.2.2. Databases

Four databases, including PubMed, Scopus, Wiley Library, and Web of Science, were queried in this review. Google Scholar was also used to supplement gray literature articles and identify studies through manual reference cross-checking. We believe these databases collectively capture the most relevant journals in the fields of geroscience, natural products, nutritional biochemistry, and molecular biology. In summary, 1355 articles were retrieved using the above keywords and databases. All retrieved references were exported to Mendeley Desktop (Version 1.19.8, Elsevier, Amsterdam, The Netherlands) for organization and duplicate removal.

#### 2.2.3. Study Selection

Following PRISMA-ScR guidelines, duplicated titles were removed (*n* = 220), leading to 1132 articles to be considered for title and abstract screening. After these articles were reviewed independently by N.M.H.H. and R.S., 828 of them were excluded due to irrelevant study populations (e.g., clinical human studies without preclinical mechanistic data), in vitro or in vivo studies involving non-plant-derived bioactive components, studies without senescence biomarker assessment, and unrelated conditions. Only 211 articles proceeded to full-text eligibility assessment, from which 111 studies met all inclusion criteria and were finally included in the review. Discrepancies between reviewers were resolved through consultation with a third reviewer (M.F.). The complete record counts for each stage are presented in the PRISMA-ScR flow diagram (Figure 1).

Methodological quality and risk of bias were assessed independently by N.M.H.H. and R.S. using the SYRCLE Risk of Bias tool for animal studies [22], evaluating selection, performance, detection, attrition, and reporting bias across 10 items. For in vitro studies, a checklist assessing senescence model validity, control inclusion, biomarker breadth, replication, statistical rigor, and reporting transparency was used. Studies were categorized as high (7–9 criteria met), moderate (4–6), or low (0–3) quality.

#### 2.2.4. Data Extraction

All selected data extracted from the included studies were organized in Microsoft Excel™. The extracted data included (1) study metadata, (2) intervention characteristics, (3) study model (cell type and senescence induction method for in vitro studies, animal species, strain, age, sex, and sample size for in vivo), (4) outcome and treatment effect data, (5) mechanistic and pathway data with additional data related to analytical approach for bioactive identification, and (6) senotherapeutic classification rationale. This approach ensures that all relevant information is recorded for thorough analysis. The complete charting table is provided as Supplementary Table S2.

#### 2.2.5. Collating, Summarizing, and Reporting Results

Following the JBI scoping review framework, the final stage was to categorize the relevant findings based on the four research questions and synthesize them. Due to substantial heterogeneity in natural products investigated, senescence models employed, and outcomes reported, quantitative meta-analysis was not performed. Instead, a narrative synthesis was conducted and supplemented with summary tables. The flow diagram for this scoping review has been summarized in Figure 1.

## 3. Results and Discussion

### 3.1. Characteristics of the Selected Studies

Following the JBI framework and the eligibility criteria outlined above, 111 relevant studies were included in the final narrative synthesis (Figure 1). Publication trends demonstrated exponential growth with 28 studies (25.5%) published in 2015–2020 and 83 studies (75%) in 2021–December 2025, such that approximately three-quarters of all included studies were published within the most recent five years. This reflects the growing interest in plant-derived senotherapeutics and recognition of cellular senescence and SASP as therapeutic targets in aging-related disease.

Study designs encompassed in vivo animal studies (*n* = 43, 38.7%), in vitro cell culture (*n* = 40, 36.0%) and complementary in vitro–in vivo mechanistic designs (*n* = 23, 20.7%). In vivo studies primarily used C57BL/6 mice aged 4 to 27 months, encompassing naturally aged rodents, progeroid syndromes (Ercc1^−/Δ^, TERT^−/−^), accelerated aging models (e.g., D-gal administration, high-fat diet (HFD)-induced metabolic senescence) and disease-specific models such as atherosclerosis (ApoE-deficient mice), ulcerative colitis, (dextran sodium sulphate (DSS)-induced), and senescence-accelerated mouse (SAMP8) strain. Primary in vitro models included human fibroblasts (dermal (IMR90), lung (WI-38), foreskin (BJ)), human umbilical vein endothelial cells (HUVECs) for vascular aging modeling, mesenchymal stem cells (MSCs) for regenerative tissue aging, and a range of tissue-specific cell lines. Senescence was most commonly induced via oxidative stress using hydrogen peroxide (H_2_O_2_), followed by replicative exhaustion (serial passaging to the Hayflick limit), genotoxic agents including bleomycin, etoposide, doxorubicin, ionizing radiation (IR), and D-galactose (D-gal) and oncogene-induced senescence (OIS) models incorporating activated oncogenes (e.g., RAS). Whole-organism studies (4.5%) employed *Caenorhabditis elegans* lifespan assays to provide a rapid, genetically tractable system and zebrafish (*Danio rerio*) aging models for longitudinal assessment of longevity and health outcomes. Treatment durations ranged from acute (3–7 days) to chronic regimens (≤9 months), administered via oral gavage, intraperitoneal injection, dietary incorporation or intermittent dosing.

With respect to compound discovery and validation methodology, the majority of included studies (*n* = 91, 82.0%) adopted a hypothesis-driven approach in which candidate compounds with established bioactive profiles were evaluated directly against senescence endpoints. Metabolomics-guided and multi-omics strategies encompassing untargeted liquid chromatography–tandem mass spectrometry (LC-MS/MS), high-performance liquid chromatography (HPLC), nuclear magnetic resonance (NMR) spectroscopy, gas chromatography–tandem mass spectrometry (GC/MS), integrated transcriptomics–metabolomics and other platforms were employed in 15 studies (13.5%), while bioassay-guided fractionation accounted for the remaining five studies (4.5%). This distribution reflects an emerging but still nascent application of metabolomics platforms in the field of plant-derived senotherapeutics. A summary of study characteristics is presented in Table 2.

### 3.2. Quality Assessment

A methodological quality assessment was assessed using the SYRCLE Risk of Bias tool for in vivo studies and a structured checklist for in vitro studies. This evaluation classified 71 studies (64.0%) as high quality, 33 studies (29.7%) as moderate, and seven studies (6.3%) as low quality. High-quality studies featured rigorous experimental designs incorporating multiple senescence and SASP biomarkers, appropriate vehicle and positive controls, sufficient biological replication (*n* ≥ 3 per group), and mechanistic investigations in in vivo or cell line systems. Moderate quality was assigned to studies employing two or more biomarkers and mechanistic data within experimental models, but lacking one or more of the additional high-quality criteria, most commonly SASP profiling, dose-response characterization or SnC selectivity viability/apoptosis assessment—that is, the failure to evaluate compound effects on proliferating non-SnCs versus SnCs. Among studies classified as low quality, common methodological limitations observed included restricted biomarker panels in which only one or no standard senescence markers were assessed, omitting mechanistic pathway descriptions, and absence of selectivity assays. The methodological limitations of moderate-to-low-quality studies do not invalidate the findings but indicate that their contributions should be interpreted as preliminary or hypothesis-generating evidence. Independent validation through expanded biomarker panels, in vivo corroboration, and standardized reporting frameworks will be essential to establish the clinical translational potential of the novel compounds and human-relevant models investigated therein.

### 3.3. Natural Senotherapeutic Characterization

The included studies identified isolated pure compounds, crude plant extracts, and multi-component formulations with senolytic and/or senomorphic properties. Senotherapeutic characterization of these agents can be understood across classification of activity, specificity and selectivity of action, mechanistic depth, in vivo translational evidence and methodological pipeline from source to validated mechanism.

The majority of activity classification was senomorphic (*n* = 51, 45.9%), followed by dual senolytic/senomorphic activity (*n* = 39, 35.1%) and pure senolytic activity (*n* = 21, 18.9%). A further 13 studies (11.7%) demonstrated concentration-dependent switching between senolytic and senomorphic modes, wherein the predominant biological activity was determined by compound dose rather than an intrinsic, fixed characteristic of a given compound (Table S2). Flavonoids constituted the most extensively investigated chemical class (*n* = 49 studies, 44.1%). Among isolated pure compounds evaluated as monotherapies, fisetin emerged as the most frequently studied (*n* = 13), followed by quercetin (*n* = 9) and a D + Q combination, with several studies additionally combining comparator and co-administered compounds, including fisetin, epigallocatechin gallate (EGCG), and sulforaphane. Additional flavonoids investigated were flavones (luteolin, apigenin), flavonol (kaempferol), flavanones (hesperetin), anthocyanins (delphinidin), flavonoid glycosides (ginkgetin, rutin), and flavan-3-ols (EGCG, PCC1). Other polyphenolic classes include curcuminoids (curcumin), chalcones (4,4′-dimethoxychalcone, 4-methoxychalcone derivatives), stilbenes (resveratrol, polydatin), and phenolic acids (chlorogenic acid, rosmarinic acid). Non-polyphenolic compounds comprised alkaloids (rutaecarpine, berberine), saponins and terpenoids (ginsenosides Rg1 and F1, astragaloside IV, gingerenone A, cycloastragenol, parishin, magnolin, shea-derived amyrins), a cardiac glycoside scaffold (ouabain, digitoxin, digoxin), and miscellaneous compounds (indole-3-carbinol, ligustilide, pyrroloquinoline).

Plant extracts and complex formulations accounted for 23 studies (20.7%), including preparations from *S. marianum*, *Zingiber officinale* extracts, *A. membranaceus*, *Agrimonia pilosa*, *Rehmannia glutinosa*, *Centella asiatica*, *Nephelium lappaceum* seeds, *Camellia Sect. Chrysantha*, *Pleurotus eryngii*, *Sonchus oleraceus*, aerial parts of *Salvia haenkei*, among others [25,26,27,28,36,37,96,97,98,99,100,101,102,108,127,128]. Four studies evaluated polyherbal or multi-extract formulations, including a dual-herb preparation of *R. glutinosa* and *A. membranaceus* [104], a traditional Chinese ophthalmic decoction [105], a proprietary two-extract combination [106], and a Tibetan polyherbal formulation (SBT) [125], while one study characterized an optimized three-component extract-derived formulation [107].

### 3.4. Senescence Biomarker and Assessment Methods

Most of the studies (*n* = 90, 81.1%) used SA-β-gal activity as a primary gold standard cellular senescence biomarker, consistent with its widespread validation as the most accessible and reproducible histochemical indicator of the senescent phenotype across both in vitro and in vivo systems. Cell cycle regulator assessments of p16 (66.7%) and p21 (61.3%) were quantified at both mRNA and protein levels. p53 protein expression and phosphorylation status were evaluated in 30 studies (27%). SASP factor evaluation was prevalent throughout. IL-6 and TNF-α (*n* = 72, 64.9%) represent the most profound measured SASP components, followed by IL-8/CXCL8 (*n* = 18, 16.2%) and IL-1β (*n* = 31, 27.9%). Additional SASP components assessed included matrix metalloproteinases (MMP-1, -3, -9, -13) (19.8%) and their tissue inhibitors (TIMPs), chemokines (MCP-1/CCL2, CXCL1), growth factors (GDF-15), and other secreted mediators (PAI-1, ICAM-1, IGFBP family member).

Oxidative stress marker quantification was incorporated into 44.1% of included studies, employing measurements of total and mitochondrial ROS (38.7%); malondialdehyde (MDA) (8.1%) as a marker of lipid peroxidation; antioxidant enzyme activities, including superoxide dismutase isoforms (SOD1 and SOD2), catalase, and glutathione peroxidase (GSH-Px); reduced-to-oxidized glutathione (GSH/GSSG) ratios; and total antioxidant capacity (T-AOC). Measurement of ROS with cell cycle arrest and SASP reflects the established mechanistic interplay between mitochondrial dysfunction, oxidative damage accumulation, and SASP amplification in the senescent phenotype. Proliferation capacity markers, including Ki67 expression and SnC-selective viability/apoptosis assays comprising MTT/CCK-8, Annexin V/PI flow cytometry, TUNEL staining, and caspase-3/7 activity measurements, were assessed in 40% of the studies. Some studies additionally employed nuclear structural markers, specifically lamin B1, as a complementary indicator of nuclear integrity (*n* = 4, 3.6%) and DNA damage response (DDR) markers (γH2AX, 53BP1), alongside telomere length measurement to provide orthogonal genomic senescence validation.

### 3.5. Compound Classes and Mechanisms of Action

**Figure 2:**
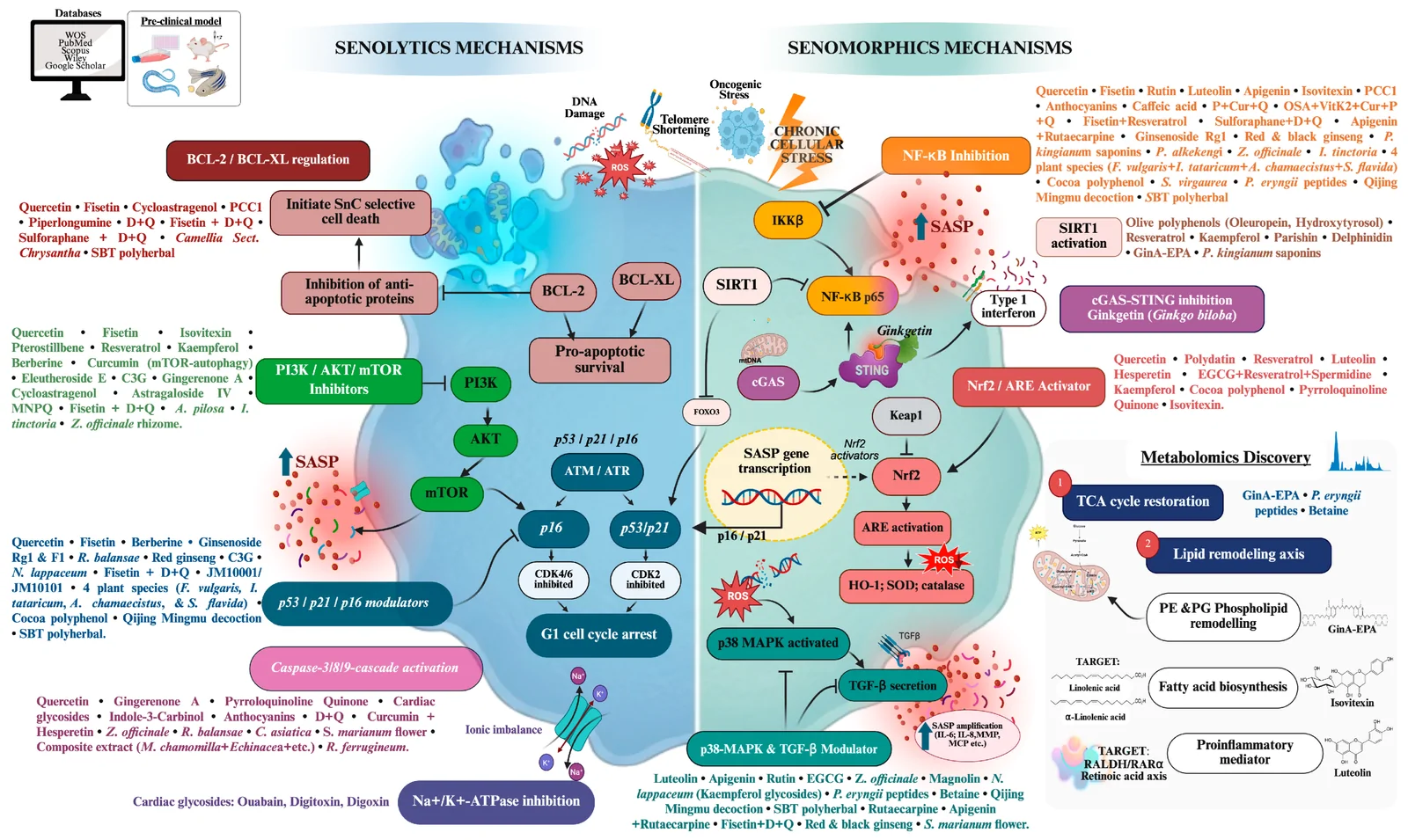
Mechanisms of action of plant-derived senotherapeutics in modulating cellular senescence organized by senolytic and senomorphic classification across signaling mechanisms, demonstrating that most bioactive compounds act pleiotropically across multiple pathway nodes. The figure is divided into two panels. The left panel (senolytic) depicts compounds that selectively eliminate SnCs via inhibition of BCL-2/BCL-XL anti-apoptotic proteins, suppression of the PI3K/AKT/mTOR survival axis, p53/p21/p16-driven cell cycle arrest, caspase-3/8/9 cascade activation, and Na^+^/K^+^-ATPase disruption. The right panel (senomorphic) depicts compounds that suppress SASP without eliminating SnCs, primarily through NF-κB inhibition, SIRT1 activation, Nrf2/ARE antioxidant induction, cGAS-STING pathway blockade, and p38-MAPK/TGF-β modulation. The central panel illustrates the convergent upstream stressors, DNA damage, telomere shortening, and oncogenic stress that activate ATM/ATR signaling, CDK inhibition, and sustained SASP transcription. A dedicated section highlights metabolomics-guided discovery of three novel mechanistic axes: TCA cycle restoration, phospholipid remodeling and RALDH/RARα retinoic acid signaling. Created in BioRender. Hussin, N. M. H. (2026). https://BioRender.com/8q5thfs (accessed on 15 July 2026).

Mechanistic findings are organized by chemical class to enable identification of patterns in pathway modulation and senotherapeutic outcome. Flavonoids are discussed first given their majority (44.1% of included studies), followed by non-flavonoid polyphenols, terpenoids and alkaloids, plant extracts and polyherbal preparations. Across classes, senolytic activity converges on disruption of SCAPs, mainly via phosphoinositide 3-kinase/protein kinase B/mammalian targets of rapamycin (PI3K/AKT/mTOR)-mediated, indirect downregulation of BCL-2/BCL-XL, whereas senomorphic activity converges on suppression of NF-κB-driven SASP transcription, reinforced by Nrf2/antioxidant and SIRT1/AMPK signaling. The mechanistic convergence of plant-derived senotherapeutics across multiple signaling pathways is summarized in Figure 2, which illustrates the major metabolic pathways of senescence targeted by these compounds.

#### 3.5.1. Flavonoids

##### Quercetin and Rutin

Quercetin (3,3′,4′,5,7-pentahydroxyflavone) can be readily found in fruits, including apples and berries, as well as in leafy greens and cruciferous vegetables and was the most extensively studied natural compound. It demonstrates remarkable pleiotropic senolytic and senomorphic effects through multi-pathway targeting of SnC vulnerabilities.

The primarily senolytic mechanism involves disruption of SnC anti-apoptotic pathways (SCAPs) via PI3K/AKT/mTOR axis inhibition [40,41,42,43]. Mechanistically, quercetin directly inhibits PI3K enzyme activity, which blocks AKT phosphorylation at threonine 308 and serine 473. This thereby represses activation of anti-apoptotic effectors, including B-cell lymphoma 2 (BCL-2) and BCL-XL, ultimately sensitizing SnCs to apoptosis. Concurrently, the activation of AMPK with mTOR suppression further enhances autophagic flux and creates a metabolic milieu unfavorable for SnC survival [40,42,43]. Because it does not bind BCL-2/BCL-XL directly, quercetin differs from BH3 mimetics such as navitoclax [43]. This indirect mode of action has implications for selectivity, as context-dependent PI3K/AKT/mTOR activity across tissue compartments may produce more cell-type-specific senolytic profiles compared with direct binding agents. Studies demonstrating effective senolytic concentration is cell-type-specific following quercetin treatment is a consistent finding, with optimal senolytic activity observed at 1 µM in bone marrow MSCs [16], 10–20 µM in nucleus pulposus cells [17], 50–100 µM in dermal and foreskin fibroblasts [39], and 20–50 μM concentrations in HUVECs and VSMCs [43]. This intercell-type heterogeneity is consistent with differential SnC dependence on PI3K/AKT survival signaling across tissue compartments and confirms that effective dose ranges cannot be generalized across model systems. A critical pharmacokinetic caveat applies with human plasma quercetin concentrations following standard supplementation, reaching only 0.3–1.6 µM [131], substantially below the 50–100 µM concentrations required for senolytic activity in fibroblast and smooth muscle models.

Senomorphic NF-κB inhibition by quercetin occurs at substantially lower concentrations (1–20 µM) than those required for senolytic effects, suppressing IL-6, IL-8, IL-1β, MCP-1, MMP-1, MMP-3, and TNF-α through prevention of IκB kinase (IKK) phosphorylation and p65/p50 nuclear translocation [16,17,38,39]. At that sub-senolytic concentration [16], quercetin preserved proliferating bone MSC viability while suppressing SASP, which supports a concentration-stratified administration strategy for clinical translation. Complementary mechanisms include activation of nuclear factor erythroid 2-related factor 2 (Nrf2) and antioxidant response element (ARE)-dependent gene expression (SOD, catalase, and heme oxygenase-1), which directly counters oxidative stress burden in SnCs. Subsequently, downregulation of microRNA-155-5p further restores Nrf2 expression and suppresses NF-κB signaling as this mechanism provides an additional layer of regulatory control over both oxidative stress and inflammatory cascades [16,17,39,41,44]. Quercetin additionally modulates p16 and p21 at both mRNA and protein levels, though the direction and magnitude of effects exhibit cell-type dependency [39,40,41,43]. SIRT1 upregulation by quercetin observed in pre-adipocyte models [39] most likely occurs via indirect AMPK-mediated NAD^+^ elevation rather than through direct enzymatic binding, distinguishing it from stilbene-class direct SIRT1 activators, though this mechanism requires further validation.

*Dasatinib Plus Quercetin (D + Q) Combination*

The combination of dasatinib, a multi-tyrosine kinase inhibitor, with quercetin (D + Q) represents synergistic senolytic efficacy across a broader spectrum of SnC types by simultaneously disrupting SCAP networks [57,58,59,60,61,65,132,133]. Dasatinib suppresses survival signaling targeting SnC subtypes, particularly preadipocytes and macrophages, that are relatively resistant to PI3K inhibition alone, while quercetin contributes PI3K/AKT-mediated BCL-2 suppression predominantly in senescent endothelial and fibroblast lineage. This mechanistic complementarity broadens SnC type coverage relative to monotherapy with either constituent. Caspase-3, -8, and -9 activation that reflects both the intrinsic and extrinsic apoptosis pathways was also documented as a downstream senolytic mechanism in IR-senescent human dermal fibroblasts [57]. In aged mice, D + Q administration demonstrated robust senolytic efficacy standardized at 5 mg/kg D + 50 mg/kg Q in intermittent 3-day cycles [59,60,64]. This intermittent “hit and run” dosing strategy, whereby SnCs eliminated during a treatment cycle are not immediately replenished, as new senescence events accumulate on a timescale of weeks to months, maximizes therapeutic benefit while minimizing off-target toxicity effects. Notably, aging mice receiving D + Q therapy exhibited reduced circulating SASP markers (IL-1α/β, IL-6, TNF-α, MCP-1, IP-10, CXCL1/2, PAI-1/2), improved physical function, extended healthspan, and favorable gut microbiome modulation [132]. Intestinal microbiome analyses revealed significant compositional shifts following D + Q, with increased beneficial genera correlating with decreased intestinal senescence and reduced systemic inflammation, suggesting senolytic efficacy may be partially mediated through gut–immune system axis modulation. Furthermore, D + Qs reportedly direct SnC elimination via immunomodulatory and microbiome-mediated mechanisms [64,132]. Camell et al. (2021) additionally demonstrated that D + Q ameliorated SARS-CoV-2-associated pathology by restoring immune cell function and pathogen-associated molecular pattern (PAMP)-induced SASP amplification dampening [58]. When sulforaphane was co-administered alongside D + Q, this regimen extended gut–brain axis modulation, synergistically targeting the gut SnC cell and modulating the microbiome, which collectively decreases SA-β-gal, p21, IL-6, ROS, MCP-1, and NF-κB pathways, reducing neuroinflammation and synaptic decline [64].

*Rutin as a Senomorphic Agent*

Rutin, a quercetin glycosylated derivative, conjugates to the disaccharide rutinose, one of the components in vegetables and fruits, especially citrus and plant-based beverages. Specifically, at 50–100 µM, rutin suppressed IL-6, IL-8, IL-1α/β, MMP-3, GM-CSF, IL-2, and IL-12 without altering p16^INK4A^ and p21^CIP1^ expression [46]. Mechanistically, rutin acts between DNA damage sensing and inflammatory signaling cascades by preventing p38/MAPK activation downstream of TAK1 kinase while maintaining ataxia-telangiectasia mutated (ATM) phosphorylation status. PI3K/AKT/mTOR axis deactivation and hypoxia-inducible factor-1α (HIF-1α) inactivation provide additional molecular explanation for rutin’s capacity to suppress NF-κB-driven SASP signaling without perturbing core senescence programming [46]. This evidence confirms rutin as a “pure” senomorphic rather than senolytic agent. In vivo validation in NOD/SCID mice at 10 mg/kg intraperitoneal weekly confirmed retention of SASP suppression in a transplant-based senescence model. This mechanistic profile positions rutin as a candidate for chronic intermittent administration to suppress the paracrine pro-inflammatory activity of slowly accumulating SnCs in aged tissues, though its senomorphic effects are expected to be reversible and the duration of benefit following treatment discontinuation has not been evaluated.

##### Fisetin

Fisetin (3,3′,4′,7-tetrahydroxyflavone) demonstrates potent senolytic activity across the broadest range of SnC types of any compound identified in this review. A systematic screening study by Yousefzadeh et al. (2018) [5] identified fisetin as the most efficacious of 10 flavonoids tested, on the basis of superior SnC clearance selectivity across multiple cell types and extension of both median and maximum lifespan in progeroid and wild-type aged mice. Cell-type selectivity distinguishes fisetin from quercetin, which demonstrates robust activity against senescent HUVECs and IMR-90 fibroblasts but limited efficacy in preadipocytes [5]. This selectivity pattern reflects differential SCAP network dependencies across tissues. HUVECs and dermal fibroblasts exhibit high BCL-2 and BCL-XL expression with strong PI3K/AKT pathway dependence for survival, rendering them sensitive to PI3K inhibitors. Preadipocytes, in contrast, show greater dependence on the ephrin-dependent and BCL-2B-mediated survival pathways with lower baseline PI3K/AKT activity that reduces their sensitivity to PI3K-mediated indirect BCL-2 suppression. Fisetin’s BCL-2 family modulation occurs primarily through indirect PI3K/AKT inhibition, though evidence from specific cellular contexts suggests fisetin may engage BCL-XL with somewhat greater potency with broader cell-type coverage at the downstream effector level [51,134].

Additionally, fisetin treatment in human skin grafts selectively eliminates senescent dermal fibroblasts, increases collagen density, and reduces SASP without apparent adverse effects for its applications in dermatological aging [57]. Intermittent administration (100 mg/kg daily for 5 consecutive days) in both Ercc1^−/Δ^ progeria and aged mice significantly reduced SnC burden across adipose, kidney, skeletal muscle, vascular, and brain tissues, with concurrent functional improvements [5,47,50]. The tissue range responsive to fisetin is further evidenced by studies reporting reduced age-related bone density loss [48], attenuation of chondrocyte senescence and osteoarthritis progression [54], clearance of senescent tubular epithelial cells in a murine lupus nephritis model [55], and reduction in cholangiocyte senescence alongside improvement of hepatic fibrosis and inflammation in a cholestatic liver model [56]. An approximately 10% extension of median lifespan in wild-type mice has also been reported when late-life fisetin supplementation is initiated [5]. However, independent replication of this lifespan extension effect across additional laboratories and mouse strains has not yet been reported and is required before this result can be treated as established evidence.

Additional mechanisms include selective apoptosis induction through both p53/p21-dependent and -independent pathways with enhanced caspase-3/7 activation confirmed specifically in SnCs across multiple studies. Interestingly, fisetin reduced intestinal SnC burden and SASP biomarkers in a murine model of DSS-induced colitis while concurrently reshaping gut microbiota composition and reduce local inflammation [52]. This is the first gut–senescence axis targeted by a single senolytic agent that has not been characterized for other flavonoids. In an aged mouse model of SARS-CoV-2 infection, fisetin administered alongside D + Q attenuated both senescence and viral entry-associated proteins, suggesting additional utility in infection-driven accelerated senescence [58]. The transforming growth factor-beta (TGF-β) pathway, which plays critical roles in SASP regulation [58] and long-term fisetin treatment, elevates urinary and tissue α-Klotho levels, an effect that contributes to systemic anti-aging potential [59]. Nonetheless, a study demonstrated sexually dimorphic responses with male mice exhibiting greater SnC burden and greater fisetin treatment response than females [61], highlighting biological sex as a potentially important variable in senotherapeutics that warrants sex-stratified analyses in future work. Moreover, although fisetin monotherapy demonstrates robust individual activity, the combination of fisetin with dasatinib (D + F) in middle-aged mice enhances the range of targeted SnC types, particularly senescent adipose tissue that is resistant to fisetin alone [60]. This evidence suggests that the mechanistic rationale for combination senolytic agents can extend the breadth of cellular targets and disease contexts through plant-derived senotherapy.

##### Kaempferol and Its Glycosidic Derivatives

Kaempferol, a dietary flavonol, demonstrated dual senolytic and senomorphic activity in H_2_O_2_-induced endothelial senescence and in vivo in ApoE^−/−^ mice on a high-fat diet. Remarkably, the supplementation reduced SA-β-gal, p16, p53, p21, and a broad SASP panel via dose-dependent Nrf2/HO-1 antioxidant pathway activation, with the lower in vivo dose (10 mg/kg) producing a paradoxically superior anti-senescence effect over 20 mg/kg, alongside eNOS upregulation, implicating vascular-protective senomorphic activity [103]. Another study identified nine kaempferol-type flavonol glycosides from *Nephelium lappaceum* seeds. The bioassay validation in replicatively senescent HDFs confirmed that kaempferol 3-O-β-d-glucopyranosyl-7-O-α-l-rhamnopyranoside, kaempferol 3-O-rutinoside, and kaempferol 7-O-α-l-rhamnopyranoside at 10 µM significantly reduced a SASP biomarker panel, which recapitulated the dual senotherapeutic profile across structurally related glycoside variants, acting through p53/p21 cell cycle arrest suppression and SIRT1 modulation [29]. Collectively, these demonstrated that both the kaempferol aglycone and its natural glycoside derivatives constitute a mechanistically coherent flavonol senotherapy cluster with tissue-context efficacy.

##### Apigenin and Luteolin: Flavone Senomorphics

Apigenin is a natural dietary flavone derived from tea, bark, fruits, and medicinal plants like chamomile (*Matricaria recutita* L.). Studies reported that this compound suppresses SASP across multiple senescence models [76,77,78,79,96] through NF-κB inhibition via interleukin-1 α /interleukin-1 receptor-associated kinase 1 and 4/p38 mitogen-activated protein kinase (IL-1α/IRAK1/4/p38MAPK) cascade, preventing p65 nuclear translocation and subsequent transcription of SASP [77]. At optimal concentrations of 1 to 10 μM, apigenin demonstrated the mechanism across various cellular contexts, including radiation, oncogene, chemotherapy, and replicative senescence models in primary human fibroblasts and prostate stromal cells with consistent reductions in IL-6, IL-8, CXCL1, and MMP-3 in the absence of cell death [76,77,78]. This confirms apigenin as a purely senomorphic profile at these concentrations. A separate study identified that apigenin is able to restore intracellular NAD^+^ levels by activating SIRT1 and suppressing CD38, which collectively reduced p16 and p21 expression in H_2_O_2_-induced senescent fibroblasts [79]. An isomer of apigenin, isovitexin extended senomorphic and senolytic activity to UVB-induced photoaged skin, where untargeted LC-MS/MS revealed a mechanistically distinct lipid metabolic modulation signature encompassing fatty acid biosynthesis, linoleic acid, and α-linolenic acid pathways alongside canonical PI3K/AKT inhibition and Nrf2/NF-κB modulation. Moreover, a synergistic combination of apigenin with rutaecarpine, an alkaloid from *Evodia rutaecarpa*, broadens senomorphic effects through TGF-β/SMAD2 pathway activation and antioxidant defense augmentation, reducing oxidative stress-induced senescence in hBMSCs while simultaneously promoting osteoblast differentiation and bone formation capacity in aged donor cells [75,76]. This combination exemplifies an important principle of multi-target synergy whereby apigenin addresses inflammatory signaling while rutaecarpine engages differentiation-promoting pathways that produce an effect profile broader than either constituent alone. Apigenin was also identified as the principal active constituent in *Silybum marianum* flower extract, where it attenuated replicative senescence-associated SASP and morphological deterioration in human hair follicle dermal papilla cells through TGF-β pathway modulation [96]. Hence, the utilization of apigenin as a senolytic needs to be considered in further studies.

Luteolin is a flavone abundantly found in *Physalis alkekengi* (FAE), *Salvia haenkei* and other plant species, suppressing senescence by operating through SIRT1-mediated p53 deacetylation, Nrf2/HO-1 antioxidant pathway activation, and mitogen-activated protein kinase/activator protein 1 (MAPK/AP-1) inhibition [23,25,26,70,71,130]. Luteolin at 10 to 20 μM indicates attenuation of the p53-driven senescence due to reduced acetylated p53 levels and restored SIRT1 activity [70]; meanwhile, it also suppressed MMP-3 and MMP-9 secretion and reduced lipid peroxidation in ultraviolet-exposed human dermal fibroblasts [71]. Metabolomic validation by Bai et al. (2025) [23] revealed that luteolin restores retinoic acid (RA) metabolism and reduces lipotoxic metabolites (arachidonic acid (AA), sphingosine-1-phosphate (S1P), and 9(S)-hydroxyoctadecadienoic acid (9(S)-HODE)) in senescent vascular endothelial cells and senescence-accelerated mice. This evidence directly links these metabolic shifts to SASP attenuation through NF-κB and immunosenescence pathway inhibition, with histological improvements observed in cardiac, vascular, and hepatic tissues [23]. This evidence positions luteolin as a senomorphic candidate for intervention aimed at anti-senescence in age-related conditions and warrants further investigation of its senolytic potential.

##### Flavan-3-Ols: Epigallocatechin Gallate (EGCG) and Procyanidin C1 (PCC1)

EGCG is the most abundant catechin in green tea. EGCG demonstrates senomorphic activity at 10 to 100 μM through multiple complementary mechanisms with context-dependent senolytic properties emerging at higher doses [63,83,84,85]. Three independent studies conducted in oxidative stress-induced senescent preadipocytes and aged macrophages consistently showed that EGCG reduces SA-β-gal activity, downregulates p53, p21^WAF1^, and p16 expression, and suppresses SASP components. The primary mechanism of EGCG involves potent dual antioxidant effects leading to direct radical scavenging, enhancement of endogenous antioxidant systems, and restoration of the cellular redox balance mediated by Nrf2/ARE pathway activation [83]. Preclinical evidence further indicates that EGCG exhibits robust anti-inflammatory activity through NF-κB-mediated and mitochondrial deacetylase SIRT3, which together inhibit the PI3K/AKT/mTOR signaling axis to reduce SASP secretion and moderate cell cycle arrest [84,85]. At doses of 50 to 100 μM, this compound induces moderate apoptosis in senescent preadipocytes [84], whereby, at the concentration above, senomorphic activity transitions towards senolysis. Interestingly, the combination of EGCG with resveratrol and spermidine achieved comparable SASP suppression at substantially lower combined doses (20 to 30 μM), with greater Nrf2 and SIRT3 induction than EGCG alone, indicating synergistic interactions within the cocktail mixture [85]. EGCG and co-administered flavonoids exhibit dual senotherapeutic mechanisms, with context-dependent pro-oxidant activity in the presence of transition metals and suppressing of the senescent phenotype in vitro [63]. While this pro-oxidant mechanism may confer senolytic benefit in certain contexts, it raises concern in iron-rich biological milieu such as bone marrow, where unintended oxidative damage to non-senescent hematopoietic progenitors could represent a safety concern. In vivo corroboration was provided by Sharma et al. (2022), whose longitudinal murine study showed that sustained EGCG supplementation at 100 mg/kg/day markedly reduced age-dependent DDRs, p53/p21-mediated cell cycle inhibition, and p38MAPK/COX-2-driven SASP activation predominantly in visceral adipose and intestinal tissues [135]. These findings extended the mechanistic scope of EGCG in cell-based studies, as chronic supplementation additionally restored mitochondrial SIRT5 expression, enhanced autophagic flux as evidenced by increased LC3II and ATG5 alongside reduced p62 accumulation and corrected excessive AMPK/AKT activation in aging tissues, collectively suggesting that mitochondrial redox maintenance and autophagy restoration represent independent senomorphic axes of this compound. At the systemic level, EGCG reduced circulating concentrations of IL-1β and TNF-α, preserved intestinal microbial diversity, and suppressed the abundance of pathogenic bacterial genera, effects accompanied by an approximately 47% lower mortality risk in treated animals, which together substantiate the translational relevance of EGCG’s anti-senescence activity beyond isolated preadipocyte models.

Moreover, PCC1 is a catechin/epicatechin trimer prominently found in grape seeds [86,87,88,136]. Its senotherapeutic classification is strictly concentration-dependent, with distinct activity thresholds demonstrated across multiple cell types. At concentrations below 50 μM, PCC1 exhibits predominantly senomorphic activity, suppressing broad SASP secretomes, including TNF-α, IL-6, iNOS, IL-1β, MMP1, and MMP3, through NF-κB pathway inhibition without inducing cell death [86]. Conversely, at concentrations of 50 μM and above, PCC1 induces robust senolytic effects through ROS-mediated mitochondrial dysfunction and selective apoptosis in SnCs [86,88]. This dual threshold of <50 μM senomorphic and ≥50 µM senolytic was reproduced consistently across diverse SnC populations, including human stromal cells, lung fibroblasts, endothelial cells, renal tubular epithelial cells, and bone marrow granulocytes, regardless of whether senescence was induced by replication, oncogene activation, γ-radiation (10 Gy) or oxidative stress (H_2_O_2_, 800 µM) [71,87,88]. Importantly, the senolytic threshold was also consistent across all tested cell types and senescence induction methods, suggesting a common ROS-mitochondrial mechanism or cell-type-agnostic mechanism rather than a cell-type-specific vulnerability. This consistency contrasts with compounds such as fisetin and quercetin, which exert marked tissue-selective activity governed by differential SCAP dependencies, and positions PCC1′s concentration threshold as a predictable, cell-type-independent pharmacological profile. The effective therapeutic window for senomorphic activity therefore spans between 5 and 50 µM (a ~10-fold range) with the senolytic threshold triggered at ≥50 µM, establishing a well-defined limit that may facilitate translational dose calibration. Recent in vivo intermittent oral supplementation at 20 mg/kg in aged mice indicates the physiological benefit of PCC1-reduced SnC burden in bone marrow and spleen, suppressed SASP profiles in B cells, and improved grip strength [87]. The same regimen was observed in the renal fibrosis model, pointing to functional tissue-level outcomes beyond marker reduction. In chemotherapy-treated mice, intermittent PCC1 supplementation was associated with a 64% extension of median post-treatment lifespan [88]. This finding requires cautious interpretation given its specificity to the model context and the absence of replication in naturally aged cohorts. Nevertheless, it supports PCC1 as a high-priority candidate pending dose-response and long-term safety validation.

##### Anthocyanin

Anthocyanins are water-soluble glycosylated derivatives of anthocyanidins, representing the largest phenolic pigments in plants. This compound functions predominantly as senomorphic through complementary anti-inflammatory, antioxidant and apoptotic mechanisms across multiple senescence models [24,91,92,93]. Cyanidin-3-glucoside (C3G), the principal compound in elderberry (*Sambucus canadensis*) extracts, was identified through ^1^H NMR-based metabolomics as a dual senolytic and senomorphic agent in this review [24]. In D-gal-induced senescent HEK293T cells, C3G-rich extracts at 100 to 400 µg/mL dose-dependently suppressed SA-β-gal, p16^INK4a^, p21^CIP1/WAF1^, p53, and intracellular ROS while upregulating antioxidant enzymes, through PI3K/AKT/mTOR pathway inhibition converging on both the p53/p21 and p16/Rb axes. In vivo confirmation in D-gal-treated Kunming mice at 80 mg/kg/day reproduced these effects alongside improved body weight and organ indices [24].

For the broader anthocyanin class, with respect to NF-κB/IKK pathway suppression, anthocyanins at 50 to 100 µM in vitro and 20 to 40 mg/kg/day in vivo reduced SA-β-gal, p16, p21, and SASP in D-gal-induced hepatocyte senescence, with concurrent hepatoprotective effects and attenuation of DNA damage [91]. Anthocyanin-mediated endothelial senescence reversal in replicative HUVECs and aged Fischer 344 rats (10 mg/kg/day for 8 weeks) extended this senomorphic activity to the reversed vascular senescence by reducing SA-β-gal, superoxide anion levels, and adhesion molecules VCAM-1 and ICAM-1 while restoring NO bioavailability and endothelial function [92]. In the central nervous system, delphinidin, an anthocyanidin monomer, attenuated microglial senescence in both amyloid-driven and naturally aged mouse models through direct SIRT1 engagement, which in turn suppressed NF-κB-mediated neuroinflammatory SASP and reduced cognitive decline markers [93]. Together, these findings indicate that anthocyanin class senotherapeutic activity spans hepatic, vascular, and neurological senescence through mechanistically distinct but complementary pathways and with C3G representing the only member of this class to exhibit confirmed senolytic activity.

##### Ginkgetin

Ginkgetin, a biflavonoid from *Ginkgo biloba*, introduces different senomorphic strategies. Unlike other compounds in this review, ginkgetin directly binds the carboxy-terminal domain of the stimulator of interferon genes (STING) protein, thereby inhibiting the cGAS-STING pathway upstream of both the interferon regulatory arm and the NF-κB activation arm [115]. During senescence, persistent DNA-damage signaling and genomic instability generate cytoplasmic chromatin fragments (CCFs) that are sensed by cGAS to activate STING; importantly, the same DDR that produces these fragments also drives ATM–p53–p21 signaling to enforce cell-cycle arrest [137]. cGAS-STING therefore lies downstream of the shared DDR but is functionally dedicated to the secretory rather than the growth-arrest output of senescence. Once activated, cGAS-STING signaling drives SASP amplification through two convergent routes, including the IRF3-dependent induction of interferon-stimulated genes (ISGs), including CXCL10, and the STING-TBK1-mediated activation of IKK, which directly phosphorylates IκB and enables p65/p50 nuclear translocation; in particular, the cGAS-STING pathway located upstream of the canonical NF-κB axis that most senomorphic flavonoids in this review target or below the IKK/p65 level. By binding directly to STING, ginkgetin inhibition therefore suppresses SASP at or above this node, halting the IRF3/ISG and NF-κB activation and eventually providing broader SASP coverage than agents targeting NF-κB alone [138]. This positions ginkgetin as a unique entry point into SnC biology that complements the NF-κB and PI3K/AKT targeting strategies employed by many included compounds. Moreover, cGAS-STING can reinforce p53 signaling, in which it creates a DDR–STING–SASP feedback loop in SnCs. Gingkgetin interrupts this loop by acting upstream of STING without directly altering p53 or p21 expression [115]. This mechanism explains ginkgetin’s ability to suppress NF-κB/IRF3-driven SASP production while preserving the p53/p21-mediated cell-cycle arrest, defining it as a pure senomorphic that acts without eliminating SnCs or reversing senescence. Ginkgetin thus offers an interesting upstream point of intervention in senescence biology that complements, rather than replicates, the NF-κB- and PI3K/AKT-directed mechanisms of the other compounds reviewed here.

#### 3.5.2. Stilbene: Resveratrol and Polydatin

Resveratrol (3,5,4′-trihydroxy-trans-stilbene) and its glycosylated derivative polydatin represent naturally occurring stilbene polyphenols that function predominantly as senomorphics. Resveratrol initiates signaling primarily through SIRT1 activation, functioning as a master regulator of cellular metabolism, stress responses, and longevity pathways [65,66,67,68]. SIRT1 activation leads to deacetylation of multiple target proteins, including p53, forkhead box O (FOXO) transcription factors, and the p65 subunit of NF-κB, which in turn attenuate transcription of pro-inflammatory mediators IL-6, IL-8, MCP-1 [62,139]. SIRT1 activation by resveratrol occurs through both direct allosteric binding to the SIRT1 enzyme and indirect mechanisms involving AMPK-mediated phosphorylation, distinguishing it mechanistically from quercetin, whose SIRT1 upregulation is exclusively indirect via NAD^+^ elevation. This SIRT1-centered mechanism also has been directly validated in cigarette-smoke-induced senescent airway epithelial cells, resveratrol downregulated miR-34a-5p, and a miR-34a-5p mimic together with a dual-luciferase reporter assay confirming that miR-34a-5p directly represses SIRT1, establishing a causal resveratrol–miR-34a-5p–SIRT1–NF-κB axis that suppresses the SASP [67]. Concurrent AMPK-mediated mTOR inhibition and Nrf2 signaling cascade activation further mitigate the elevated ROS burden characteristic of SnCs. Remarkably, Jacob et al. demonstrated that the combination of resveratrol and fisetin suppressed senescent human osteoarthritic chondrocytes more effectively than either compound alone at the musculoskeletal compartment [62]. This combination of fisetin aligns with the mechanistic profile of resveratrol with respect to the SIRT1-mediated senomorphic and the BCL-2/BCL-XL-directed senolytic activity of fisetin, enabling simultaneous attenuation of both SnCs and the pro-inflammatory tissue environment. In contrast, consistent with prior reports dating to Demidenko & Bhatt (2008), a critical finding revealed context-dependent effects, demonstrating that resveratrol paradoxically induced senescence in human dermal fibroblasts at ≥25 μM through ATM-p53-p21 pathway activation and SIRT1 downregulation [65]. This concentration-dependent reversal of senomorphic activity to pro-senescent underscores that resveratrol’s senotherapeutic window is exceptionally narrow. Human plasma resveratrol concentrations following standard dietary intake are approximately 0.01–0.1 µM, rising to 0.3–2 µM with supplementation [134], which is well below both reported therapeutic (10–20 µM) and pro-senescent (≥25 µM) in vitro thresholds. This pharmacokinetic reality suggests resveratrol is most appropriately positioned as a low-dose preventive intervention or multi-targeted combination component, rather than a standalone senolytic or high-dose senomorphic agent.

On the other hand, polydatin demonstrated a wider apparent therapeutic window (10–400 μM in vitro; 50 mg/kg/day in rats) with potentially improved aqueous solubility relative to resveratrol. In nucleus pulposus cells with TNF-α-induced senescence, polydatin (at 200 to 400 µM) reduced senescence markers (SA-β-gal, p53, p16), suppressed matrix-degrading enzymes, and restored extracellular matrix components, specifically aggrecan and collagen II, indicating that its senomorphic activity encompasses tissue-level matrix preservation beyond SASP suppression [69]. Unlike resveratrol, polydatin also operates through Nrf2/Keap1 pathway activation and mitochondrial antioxidant protection, despite sharing the same stilbene scaffold [69]. The combination of polydatin with curcumin and quercetin [45] with orthosilicic acid, vitamin K2, curcumin, and quercetin [38] exhibited superior anti-senescence effects by suppression of inflammaging-associated microRNAs and enhanced osteogenic differentiation capacity in human MSCs and HUVECs, respectively, compared to individual compounds. This combination acts synergistically through NF-κB pathway inhibition, which the authors reported as a synergistic interaction based on multi-marker suppression profiles.

#### 3.5.3. Curcuminoid: Curcumin

Curcumin, a polyphenolic compound derived from *Curcuma longa* (turmeric), exhibits primarily senomorphic effects, suppressing SASP components including IL-6, IL-8, MCP-1, and MMP through NF-κB signaling inhibition [38,45]. Additional mechanisms include activation of Nrf2-dependent antioxidant responses, collectively reducing oxidative stress burden [72,136]. Curcumin also modulates PI3K/AKT/mTOR and SIRT/AMPK/mTOR pathways, though with lower potency compared to quercetin and fisetin [73]. At the cellular level, curcuminoids are associated with SIRT1 and promote AMPK phosphorylation, which collectively induces autophagy through mTOR-dependent mechanisms, reduces intracellular oxidative stress, and downregulates p16 and p53 expression under a dose-dependent manner [73]. When dealing with malignant cells, curcumin induces senescence by upregulating the p21^WAF1/CIP1^ and p53 pathways that lead to DDR, which in turn forces the cancer cells to permanently halt replication [140].

However, curcumin suffers from severely limited bioavailability (below 1%), attributable to low aqueous solubility, poor gastrointestinal absorption, and rapid phase II metabolism. Hesperetin as a single agent exerted senomorphic activity in a tissue-injury context, suppressing SASP cytokines (IL-6, TNF-α) and fibrotic collagen deposition in a bleomycin-induced pulmonary fibrosis model (A549 cells and C57BL/6 mice) via the autophagy–restoration axis and concurrent Nrf2 activation [118]. Curcumin combined with hesperetin further produced dual senomorphic and senolytic activity in senescent neuronal cells, in both in vitro and in vivo studies, for neurodegeneration-associated senescence [74]. The strategic pairing of curcumin (a senomorphic and pro-senescent oncogenic inducer) with potent senolytic quercetin and fisetin facilitates the targeted depletion of the malignant cell population without allowing surviving cells to fuel recurrence or tissue damage via SASP [140]. Notably, a study conducted by Chu et al. (2025) reported that curcumin encapsulated in neutrophil-derived nanovesicles (Cur-NV) enables blood–brain barrier penetration for SnC clearance in an Alzheimer’s mouse model while maintaining p16/p21 and SASP suppression [72]. This targeted delivery strategy decouples efficacy from systemic challenges and represents a promising translational avenue for curcumin-based senotherapy for CNS. Curcumin analogs or targeted delivery warrant further investigation to enhance translational potential beyond current combination therapy applications.

#### 3.5.4. Other Non-Flavonoid Compounds

Parishin (*Gastrodia elata*) is a phenolic glycoside which exerts cardioprotective senomorphic effects by upregulating SIRT1 and Klotho/FoxO1 pathways, thereby reducing hypertrophy, fibrosis, and SASP markers while increasing lamin B1 in senescent human coronary artery endothelial cells and naturally aged mice [94]. Transcriptomic analysis in a second in vivo study confirmed SIRT1 activation as the dominant mechanism, with downregulation of Gja1 and senescence-associated transcripts in cardiac tissue, alongside reduced p16, p21, and growth differentiation factor 15 (GDF15). Specificity is further refined in chalcone derivatives, where methoxy substitution at specific ring positions enhances senolytic selectivity that provides a structure–activity relationship rationale for natural product optimization [121]. Caffeic acid attenuated bleomycin-induced pulmonary fibrosis alongside SASP suppression in lung cells [123], while eleutheroside E and magnolin both targeted skin senescence, which uniquely demonstrated statistically significant reductions in wrinkling (11.4%), roughness (22.9%), and improvements in elasticity (12.7%) alongside decreased p16, p21, and MMP expression in fibroblasts [117,122]. Ginger derivatives (gingerenone A and shogaol) and cycloastragenol, a triterpenoid saponin from *A. membranaceus*, exert dual functionality via inducing SnC apoptosis through BCL-family suppression and PI3K/AKT/mTOR inhibition, reducing senescence markers and dampening SASP expression [33,34,112,133]. A GinA EPA-conjugated modified form exhibited enhanced potency in aged and doxorubicin-treated mice, reducing p16-positive cells in lung tissue and IL-6 at treatment withdrawal (washout), with multi-omics metabolomics confirming AMPK/SIRT1 activation, TCA cycle normalization, and mitochondrial membrane remodeling as downstream effectors [33].

Ginseng-derived compounds mostly belong to terpenoids and demonstrate mechanistic diversity across multiple senescence-relevant pathways. Ginsenosides Rg1 isolated from *Panax* species inhibits toll-like receptor 2/myeloid differentiation and the primary response 88/nuclear factor-kappa B (TLR2/MyD88/NF-κB) pathway coupled with TNF-α signaling downregulation and suppression of SASP-associated cytokines, producing dose-dependent reductions in SA-β-gal, p53, p21CIP1, and ROS at 50 µM in in vitro models and 40 mg/kg/day in D-gal-induced animal models [80]. Furthermore, red and black ginseng extracts demonstrate senomorphic mechanisms through NF-κB pathway inhibition, and TGF-β signaling regulation, with red ginseng further inducing apoptosis in SnC via the p53/p21 axis [81,82]. Ginsenoside F1, a Rg1 metabolite, targets senescence through a telomere-preservation mechanism, thereby restoring TRF2 expression in aged human somatic cells, suppressing DDR activation, and improving mitochondrial function [141]. Molecular docking identified F1 binding proximal to the TRF2 dimerization domain (~8.3 kcal/mol), suggesting stabilization of homodimer formation as a novel anti-senescence mechanism absent from classical compound frameworks [141]. The mechanistic diversity of ginseng-derived compounds, spanning NF-κB/SASP suppression, p53/p21 modulation, and TRF2-mediated telomere preservation, positions them among the most mechanistically versatile candidates in terms of natural senotherapeutics for age-related intervention.

In contrast, plant alkaloids and amide-class compounds have two distinct mechanisms as senolytics that need attention. Berberine occupies a distinct niche by acting on the cell cycle directly via modulation of p16/cyclin D1/CDK4/RB and inhibiting mTOR to extend the replicative lifespan in yeast and human fibroblasts and reverse the senescent phenotype in aged mice [89]. Piperlongumine induced ROS-dependent proteasomal degradation of oxidation resistance protein 1 (OXR1), a regulatory protein that normally protects SnCs from oxidative stress-induced death, resulting in selective elimination of SnC across IR-, replicative-, and oncogene-induced models without equivalent cytotoxicity in proliferating cells and in vivo efficacy confirmed in naturally aged mice [111]. Indole-3-carbinol induced selective apoptosis in senescent MEFs and primary human fibroblasts under replicative senescence, establishing senolytic activity via BCL-2 family inhibition. Finally, this structurally diverse repository of natural compounds, ranging from regulators of ROS and metabolic checkpoints to addressing neurodegenerative and microbiome-mediated pathways, greatly expands the senotherapeutic paradigm beyond flavonoid-centric approaches. Interestingly, these agents offer additional strategic opportunities to target heterogeneous SnC populations across tissue types and age-related pathological conditions.

#### 3.5.5. Crude Plant Extracts and Polyherbal Formulation

Plant-derived senotherapeutics span a continuum from purified compounds with defined molecular targets to complex extract preparations whose bioactive constituents remain partially or wholly uncharacterized. The majority of extract-based studies included in this review are rich in polyphenols, flavonoids, alkaloids, saponins and other classes. The central interpretive challenge across extract-based studies is determining whether reported activity reflects a single major constituent, cooperative action among co-occurring metabolites, or matrix-enhanced bioavailability of a minor constituent. This distinction has direct implications for standardization, reproducibility, mechanism and translation of extract-based findings.

##### Senolytic Mechanisms

Several plant species demonstrate selective cytotoxicity toward SnCs through apoptotic pathway activation. *R. balansae* and Alpine Rose (*R. ferrugineum*) leaf extracts inducing caspase-3/7-mediated programmed cell death with high selectivity for replicative senescent fibroblasts and the p53/p21 pathway axis, with in vivo improvements in skin elasticity [27]. This activity was suggested to be initiated by its isolated phenolic compound, specifically Rhamnoneuronal C and Balansechromanones A and B. Similarly, extracts from *Camellia sect*. *Chrysanthaa* species reduce the BCL-2/Bax ratio to trigger mitochondrial-mediated apoptosis in D-gal-induced senescent cardiomyocytes through BCL-2 family protein downregulation alongside p53, p16I^NK4a^, and p21^CIP1/WAF1^ [30]. This represents an indirect PI3K inhibition profile mechanism consistent with flavonoid class. A mechanistically distinct approach occurs from shea-derived triterpenoids, wherein α-amyrin and β-amyrin promoted SnC elimination through cannabinoid receptor 2 (CB2R) signaling pathways independent of classical caspase activation, representing a novel non-apoptotic route [127]. *A. pilosa* and its constituent, Agrimol B, exhibited PI3K/AKT-mediated senolytic and senomorphic properties in dox-induced senescent WI-38 fibroblasts; in turn, dietary supplementation in aged C57BL/6 mice confirmed effective reduction in SnC accumulation [129]. *I. tinctoria* ethanolic extracts similarly induced apoptosis in senescent HDFs through combined inhibition of NF-κB and mTOR signaling cascades, while simultaneously reducing IL-6 secretion, though senolytic activity was restricted to skin fibroblasts and not observed in IMR90 lung fibroblasts [108]. However, the specific bioactive constituents responsible for these effects remain uncharacterized.

The composite botanical study by Matos et al. [98], who evaluated chamomile, goldenrod, and green tea extracts in etoposide-induced senescent human dermal fibroblasts, revealed a critical divergence between senolytic and senomorphic activity profiles within a single experimental system. All three extracts reduced SA-β-gal activity, confirming senolytic capacity; however, chamomile extracts paradoxically elevated multiple SASP components, including IL-6, IL-8, IL-1β, IL-1α, CXCL-1, MMP-3, and HLA-E, while goldenrod selectively suppressed IL-6 and green tea produced no significant SASP modulation. These divergent SASP profiles within a chemical combination system demonstrate that reduction in SA-β-gal activity cannot be used as a surrogate for SASP suppression and highlight the risk of pro-inflammatory outcomes from botanicals with apparent senolytic activity. Two compounds from *A. membranaceus* illustrate complementary mechanisms within a single botanical source. Cycloastragenol, a triterpenoid saponin derived from that plant, acted as a senolytic by inhibiting BCL-2 family proteins and PI3K/AKT/mTOR signaling, selectively reducing SnC viability alongside p16, p21, and SASP markers across both etoposide-induced and replicative senescence models in vitro and irradiation-induced aging in vivo [34]. Astragaloside IV suppressed multiple SASP cytokines while upregulating nuclear lamin B1 and reduced mitochondrial ROS in *n* LPS- and MPP^+^-induced senescent astrocytes by promoting PINK1-dependent mitophagy and autophagy flux [113]. These two Astragalus-derived compounds illustrate how chemical diversification within a single plant genus can be leveraged to address distinct mechanistic nodes of the SnC phenotype, offering a rational basis for combination or sequential use.

##### Senomorphic Mechanism and SASP Modulation

Multiple extracts function as senomorphics and modulate cell cycle checkpoints without inducing cell elimination. *Z. officinale* rhizome extracts significantly attenuated ROS, IL-6, IL-1β, IL-8, MCP-1, ICAM-1 and TNF-α through NF-κB/p65 inhibition, p38 MAPK suppression and PI3K/AKT/mTOR pathway modulation, alongside microRNA-126 modulation across keratinocytes, fibroblasts, endothelial cells, and microglia, thereby dampening SASP-mediated tissue dysfunction [98,102]. Notably, SA-β-gal activity was unaltered and p16I^NK4a^ showed an unexpected increase in senescent HUVECs, confirming a “pure” senomorphic profile focused on inflammatory resolution. The 50% ethanol *C. asiatica* extract demonstrates dual senotherapeutics activity through antioxidant and pro-apoptotic mechanisms where this extract reduced SA-β-gal, p16, p21, and ROS while also inducing apoptosis in H_2_O_2_-induced senescent HaCaT keratinocytes [126]. According to a study by Sun et al. (2020), luteolin, rutin, and saponins are the major active components in FAE fruit extract that mediate SnC clearance, as evidenced by reduced SA-β-gal in the liver, plasma, and hippocampus of D-gal-aged ICR mice and improved antioxidant enzyme activities alongside cognitive performance, fatigue resistance and ameliorated organ injuries [25]. In addition, the standardized ethyl acetate of FAE containing luteolin glucosides and physalins replicated these effects across five cellular models, including fibroblasts, macrophages, keratinocytes, reconstituted human epidermis, and zebrafish embryos, consistently reducing SASP markers and oxidative stress through NF-κB pathway suppression [26]. Luteolin and the luteolin-7-O-glucuronide component of ethyl acetate FAE and *S. haenkei* extracts demonstrated dual senolytic and senomorphic activity in aged mice. The senolytic mechanism involves disruption of the p16–CDK6 interaction that sustains G1/S cell cycle arrest while simultaneously SIRT1 pathway activation and NF-κB inhibition contribute antioxidant and SASP-suppressive senomorphic effects [25,130]. Markedly, this suggests that synergistic interaction among co-occurring compounds within a single extract is required to achieve full senolytic potency as this activity was not observed for luteolin monotherapy, where its senomorphic activity was retained.

*Cistus* essential oils (*C. laurifolius* and *C. monspeliensis*) at 100 ng/mL enhance mitochondrial biogenesis, DNA content, and promote expression of differentiation (involucrin, loricrin) in UVB-induced senescence in HaCaT keratinocytes [99]. Olive-derived polyphenols (oleuropein and hydroxytyrosol) reduce SA-β-gal and SASP secretomes and upregulate chondrogenic differentiation markers (COL2A1, aggrecan) in both in vitro and in vivo models [100]. Both extracts acted as senotherapeutics via activated SIRT1-dependent pathways. Furthermore, cocoa polyphenol extracts coordinated activation of AMPK, NF-κB, Nrf2, and p53/p21 pathways as well as upregulation of SIRT1/ SIRT3 that regulate p53 acetylation status and mitochondrial function in MSCs and auditory cells, with applications in age-related hearing preservation and tissue regeneration [109]. *Rosa roxburghii* fruit extracts employ a telomere targeted strategy by upregulating hTERT and telomerase expression while suppressing p53, p21, and p16 to delay replicative senescence in dermal fibroblasts [101]. *Polygonatum kingianum* saponins engaged multiple longevity-associated pathways, including SIRT1 axis orthologue sir-2.1, FoxO/DAF-16 nuclear translocation, and autophagic flux induction, besides reducing senescence and SASP markers in H_2_O_2_-induced senescent fibroblasts. Also, *P. kingianum* saponins extended lifespan and improved healthspan indicators, including body bending frequency, pharyngeal pumping rate, and lipofuscin accumulation in *C. elegans* [124], providing multi-level evidence spanning molecular, cellular, and organismal endpoints.

Sub-senolytic quercetin concentrations (20 µM or below) preserved proliferating MSC viability while suppressing SASP; however, concentrations required for senolytic efficacy in fibroblasts (50 µM or above) carry cytotoxicity risk for rapidly dividing progenitor populations [16]. The senolytic activity of extracts including *R. balansae*, *Camellia species*, *I. tinctoria* and *P. kingianum* has not been assessed for selectivity against non-senescent progenitor cells, requiring safety assessments before these extracts can be clinically used.

##### Polyherbal Formulation and Synergistic Bioactivity

Evidence synthesized in this review also supports the advancement of polyherbal formulations for senotherapeutic applications. Complex botanical mixtures offer potential benefits, including synergistic or additive effects from constituents that represent strategic integration of complementary molecular pathways, broader SnC-type coverage through diverse selectivity profiles, enhanced bioavailability via constituent interactions affecting absorption and metabolism, improved safety profiles attributable to dose reduction in individual components within an active mixture, and empirical validation reflecting centuries of traditional medicinal optimization [142].

The Qijing Mingmu decoction is a seven-herb formulation containing *L. barbarum*, *Polygonatum*, *O. japonicus*, *P. cocos*, *Glycyrrhiza*, *E. prostrata*, and *L. striatum* and was evaluated for its anti-senescence in conjunctival fibroblast senescence [105]. This decoction at 1.61 mg/L simultaneously inhibits p53/p21 and p16/Rb and downregulates MAPK/NF-κB signaling, accompanied by reductions in SASP and intracellular ROS. Additionally, a study by Imb et al. (2024) and colleagues evaluated composite extracts combining aqueous extract of chamomile (*M. chamomilla*), 40% ethanolic *S. virgaurea* extract, aqueous green tea extract, and 40% ethanolic *G. lucidum* extract, demonstrating dual senolytic–senomorphic activity [98]. Chamomile extract elevated multiple pro-inflammatory mediators via individual testing, showing a paradoxical SASP amplification profile that would constitute an adverse outcome in isolation. In contrast, goldenrod extract suppressed IL-6, and green tea produced no significant SASP modulation. The combination extracts reduced SA-β-gal activity and attenuated multiple SASP cytokines (IL-6, IL-1β, IL-8) with antioxidant protection in HDFs. This pattern, where individual extracts with divergent individual effects produce a superior combined outcome, is consistent with genuine synergy rather than simple additivity. As with all individual and combined extracts sharing equivalent senolytic activity, in this case, SA-β-gal reduction is not a reliable surrogate for beneficial senotherapeutic outcomes, and SASP characterization must be performed independently for each constituent of any multi-extract formulation before synergy claims can be substantiated. In hematopoietic stem cells, the combination of *R. glutinosa* and *A. membranaceus* preserved quiescence and self-renewal capacity through integrated mechanisms involving ROS reduction and p18^INK4C^ upregulation, with subsequent p53/p16^INK4A^ suppression [104]. Notably, this coordinated response maintains stem cell function, enhanced B-cell immunity, and extended lifespan in aged mice. This finding explicitly demonstrates the capacity of a plant-derived senomorphic preparation to benefit non-senescent stem cell populations by reducing the pro-inflammatory microenvironment exerted by accumulating SnCs in the bone marrow niche.

JM10001 (*Salsola collina* crude extract) and its optimized derivative JM10101 (comprising quercetin + β-sitosterol + salicylic acid at 6.2 mg/L) demonstrate a rational “botanical deconvolution” strategy in which initial crude extract bioactivity guided identification of a minimal effective multi-component formulation with improved standardization and mechanistic clarity [106]. JM10101 suppressed p16, p21, p53, and SASP (IL-6, IL-1β) across human MRC-5 and murine MEF fibroblast models at approximately eight-fold lower total mass concentration than JM10001 (JM10001: 50 mg/L; JM10101: 6.2 mg/L), supporting constituent-level synergy among the three components [106]. In vivo validation reported cross-species translational potential due to the ability of JM10101 to attenuate dox-induced accelerated senescence and ameliorate age-related phenotypes in aged mice, along with healthspan extension in *C. elegans*. The mechanistic attribution spans DDR mitigation, p53 pathway modulation, antioxidant activity, and a dual senomorphlytic profile. This rational combination, wherein botanical screening identifies active crude extracts to establish minimal effective multi-component formulations that offer improved standardization and mechanistic clarity compared to complex whole-plant preparations, retains senotherapeutic efficacy through synergistic constituent interactions.

Wu et al. (2025) evaluated SBT (Srolo Bzhtang), a Tibetan polyherbal formulation comprising *Solms-laubachia eurycarpa*, *B. purpurascens*, *L. lacca*, and *G. uralensis*, across D-gal-induced senescent H9c2 cardiomyocytes and H_2_O_2_-stressed zebrafish embryos [125]. SBT produced dose-dependent reductions in SA-β-gal activity (41.2% to 20% across the tested concentration range), suppressed p53 and p21, elevated SIRT1 at both protein and mRNA levels, and restored antioxidant defenses in both models, with concurrent improvements in zebrafish survival rate and restored cardiac function, providing in vivo translational support. The contribution of individual constituents, specifically *Bergenía* (a known source of bergenin and catechin), *Glycyrrhiza* (glycyrrhizin, isoliquiritigenin), and *Solms-laubachia* polyphenols, to the observed multi-pathway profile warrants constituent-level deconvolution in future study. Therefore, translation of plant-derived and formulation-based senotherapeutics into dietary supplements and therapeutic interventions should prioritize bioactive compound characterization and standardization within crude extracts, elucidation of synergistic interactions in formulations, and rigorous safety and efficacy profile evaluation across preclinical and clinical models for a promising class of healthy aging strategies.

### 3.6. Principal Findings and Integration with Existing Evidence

This current review confirms and extends the mechanistic convergence regarding natural senotherapeutics, while revealing critical divergence in cell-type selectivity, tissue specificity, and the mechanistic depth achievable through different analytical discovery approaches. Consistent with recent reviews [9,143], our findings indicate that natural compounds primarily target SCAPs and SASP regulatory mechanisms. NF-κB pathway suppression constituted the most prevalent reported primary signaling mechanism (*n* = 54, 51.9%), targeted predominantly by flavonoids, stilbenes, and extract-based preparations with senomorphic outcomes. Nrf2-dependent antioxidant and ROS-related pathway modulation was documented in 33 studies (31.7%), frequently co-occurring with NF-κB suppression in polyphenol-rich preparations. BCL-2 family protein modulation and apoptosis induction accounted for 22 studies (21.2%). The p53/p21 pathway was the primary mechanism in 47 studies (45.2%), followed by PI3K/AKT/mTOR inhibition (*n* = 39, 37.5%), modulated predominantly by quercetin, fisetin, PC1, cycloastragenol and crude extracts for targeted senolytic mechanism. SIRT1 activation was reported in 29 studies, (27.9%), and AMPK activation (*n* = 7, 6.3%) were identified as additional primary mechanisms. A compound-class to pathway analysis reveals that flavonols predominantly target PI3K/AKT/mTOR and NF-κB, stilbenes converge on SIRT1 and AMPK/mTOR, while terpenoids and saponins show the broadest pathway distribution, including PI3K/AKT, BCL-2, PINK1-dependent mitophagy, and FoxO transcription factor activation. Extract-based preparations consistently engaged multiple pathways simultaneously, consistent with their multicomponent composition and supporting the rationale for polyherbal formulation strategy approaches in contexts requiring broad SnC type coverage. The identification of the cGAS-STING pathway as a direct senotherapeutic target for ginkgetin [115] introduces an important mechanistically novel finding of this review. Importantly, genuine senomorphic activity engages the upstream transcriptional and signaling machinery that governs SASP expression, most prominently NF-κB, p38 MAPK, and cGAS-STING, and is therefore distinct from the non-specific anti-inflammatory or antioxidant effects that only lower inflammation and oxidative stress respectively. This is also consistent with emerging evidence that cytoplasmic chromatin sensing is a primary SASP driver [144]. Table 3 presents the frequency and primary senotherapeutic role of major signaling pathways identified across the 111 included studies. TCA cycle metabolic restoration, emerging from gingerenone A and its modified derivative GinA-EPA [33], *P. eryngii* peptide [36], and betaine [37] studies, further identifies central carbon metabolism as a newly recognized senomorphic intervention axis beyond canonical signaling pathway targets.

Cell-type-specific activity patterns represent the most consistent and clinically significant finding across the evidence. Quercetin demonstrates robust senolytic efficacy in HUVECs and VSMCs but limited activity in preadipocytes; meanwhile, fisetin shows robust activity against senescent HUVECs and IMR-90 fibroblasts but limited efficacy in the same preadipocyte population [5,43,50,51]. These patterns reflect differential SCAP network dependencies that characterize each SnC type and have direct implications for tissue-targeted therapeutic development, where efficacy demonstrated in one cell type cannot be assumed to generalize across tissues. HUVECs and dermal fibroblasts maintain survival predominantly through elevated BCL-2 and BCL-XL expression, which is sustained by constitutively active PI3K/AKT signaling [50,51,53]. Both fisetin and quercetin indirectly reduce BCL-2 and BCL-XL protein levels by targeting the PI3K/AKT pathway upstream, in which this upstream inhibition is sufficient to destabilize the anti-apoptotic threshold and initiate selective SnC apoptosis in these cell types [40,51]. Preadipocytes exhibit comparatively lower basal PI3K/AKT activity and depend instead on ephrin-B receptor signaling and BCL-2B-mediated survival pathways for anti-apoptotic protection [8]. It is notable that in vivo fisetin administration does reduce overall SnC burden in adipose tissue, as reported by Yousefzadeh et al. [5]. This organ-level reduction, however, most plausibly reflects the elimination of PI3K/AKT-dependent endothelial and stromal SnC subpopulations residing within the fat tissue rather than direct clearance of preadipocytes themselves [50,53]. Quercetin’s senolytic activity is strongly PI3K/AKT-dependent; meanwhile, fisetin engages a broadly similar indirect mechanism yet shows somewhat greater BCL-XL coverage and a wider tissue range. Both differ from PCC1, whose senolysis at ≥50 µM is driven not by PI3K/AKT–BCL-2 signaling but by ROS-mediated mitochondrial dysfunction, and from cycloastragenol, which combines BCL-2-family suppression with direct PI3K/AKT/mTOR inhibition. This heterogeneity also provides a mechanistic rationale for combination strategies such as D + Q, D + F, and fisetin + resveratrol or sequential strategies [57,58,59,60,61], where complementary selectivity profiles expand the breadth of SnC types targeted and necessary for systemic application.

An important methodological concern identified in this review is senescence induction model bias. Systematic analysis in Table 4 and Table S2 identifies that oxidative H_2_O_2_ SIPS (*n* = 22) and D-gal-accelerated aging models (*n* = 12) collectively dominated the induction evidence, yet these models may not truly recapitulate the SCAP network architecture of chronological senescence with regard to BCL-2 family expression profiles and PI3K/AKT dependence. In contrast, oncogene-induced senescence (OIS), which is mechanistically distinct from SIPS and replicative models through RAS-ERK/p16/Rb crosstalk, was represented in only seven studies (6.3%). Future studies should employ at least two distinct induction methods, preferably replicative senescence alongside a chemically induced or physiologically relevant model, and validate findings in naturally aged animal models to establish robustness of senotherapeutic activity. Moreover, biomarker reporting was inconsistent, in which SA-β-gal was near-universal (81.1%), yet SnCs can also express the cell cycle inhibitors p16^Ink4a^ and/or p21^Cip1^ and evidence of DDR alongside characteristic morphological changes. Such features are readily observed in vitro but far harder to resolve in vivo, where SnCs are sparse and intermixed with non-SnCs. The phenotypic phenomenon can be triggered by a variety of different stimuli, such as telomere attrition, different types of DNA damage, epigenetic alterations, oncogene activation, organelle (including mitochondrial) damage, and oxidative stress. Also, the cell-type selectivity and origin might contribute to the biomarker characteristics. Importantly, only 40% of the included studies employed SnC-selectivity assays, which are the gold-standard tests required for defining a true senolytic. Thus, there is no universal biomarker for these cells, regardless of inducer or cell type. Current guidance for assessing senescence in vitro and in vivo recommends that combining a range of complementary markers would be more effective [145] than relying on a single marker. For the secretory phenotype, the SASP Atlas [6] now catalogues the core (e.g., IL-6, IL-8, MMP1) and context-dependent array of SASP factors across human cell types, including fibroblasts and endothelial, epithelial and preadipocytes cells that have undergone replicative-, irradiation-, and oncogene-induced senescence, uncovering the variability and complexity of SASP factors not captured by traditional inflammatory markers. This represents a primary constraint on evidence synthesis and emphasizes the need for adoption of a minimum standardized biomarker panel regardless of transcriptional gene, morphology or metabolite changes in future senotherapeutic research.

Safety considerations require careful attention despite generally favorable preclinical safety profiles. The paradoxical pro-senescent effect of resveratrol at 25 µM or above [65], EGCG’s context-dependent pro-oxidant activity, and the incomplete dose-response characterization in most included studies underscore the need for systematic therapeutic window definition. Intermittent senolytic dosing employed in most in vivo studies is expected to mitigate cumulative toxicity compared with continuous senomorphic administration; long-term surveillance data remain absent, but are essential as senotherapeutics progress toward clinical implementation. In theory, intermittent dosing of senolytics like D + Q and Fisetin widens the therapeutic window due to the short half-life [146]. Senolytics are cleared from the body within a few hours to permanently remove SnCs, but the physiological benefits last for months without needing to stay in the body to keep blocking the protein or enzyme. Because it takes weeks or months for a new population of SnCs to re-accumulate, continuous presence of the drug is entirely unnecessary. Regarding tissue-selectivity of senomorphic effects, available evidence drawn from rutin and quercetin at sub-senolytic concentrations indicates that SASP suppression is reversible within days of treatment discontinuation, as senomorphics suppress but do not eliminate SnC populations responsible for ongoing SASP production [46]. A study incorporated a treatment-withdrawal (washout) arm of gingerenone A/GinA-EPA, which reported persistently reduced IL-6 at washout, but because this agent also possesses senolytic activity, its durable effect more plausibly reflects SnC clearance than reversible senomorphic suppression and therefore does not isolate senomorphic reversibility. This reversibility implies that clinical senomorphic applications would require sustained or intermittent maintenance dosing schedules, in contrast to senolytic “hit-and-run” approaches. Current evidence derives predominantly from preclinical models employing doses selected for mechanistic proof-of-concept that limit inferences regarding therapeutic windows, maximum tolerated doses, and dose-limiting toxicities relevant to human applications. This is especially critical in geriatric populations with altered pharmacokinetics, hepatic metabolic capacity and polypharmacy interactions.

Integration across studies reveals an emerging gut microbiome axis as a shared and potentially conserved feature of plant-derived senolytic activity. D + Q administration in aged mice reduced intestinal SnC burden and produced distinct gut microbiota signatures, including elevated *Akkermansia muciniphila* and a reduced *Firmicutes*-to-*Bacteroidetes* ratio that correlated negatively with senescence and inflammatory markers [131]. Saccon et al. proposed that these benefits may involve microbiome modulation as an additional mechanism alongside senolysis, noting that quercetin has a documented effect of directly regulating gut microbiota composition in animal models [131]. Furthermore, gut microbiota reciprocally metabolize quercetin and regulate its intestinal bioavailability, thereby establishing a bidirectional relationship between senolytic agent and the microbial community [131]. Fisetin and EGCG monotherapy produces comparable microbiome effects. In a murine colitis model, fisetin reduced colonic SnC burden, suppressed inflammatory markers, including MCP-1 and CXCL1, and selectively promoted *A. muciniphila*. This compound also restored Firmicutes and increased *Ruminococcus* and Verrucomicrobia, bacteria associated with short-chain fatty acid production and support for gut barrier function, thereby counteracting dysbiosis produced by intestinal injury [52].

Long-term supplementation of EGCG in aged mice preserved age-associated loss of microbial alpha diversity and reduced inter-individual beta diversity dissimilarity, effects accompanied by significant suppression of pathogenic and opportunistic genera, including *Clostridium*, *Staphylococcus*, *Streptococcus*, *Pseudomonas*, and *Enterococcus*, without significantly altering the commensal *Lactobacillus* population [135]. That mechanistically distinct senolytic regimens converge on shared beneficial microbiome outcomes suggests that gut microbiota remodeling may be a broadly conserved feature of organismal response to plant-derived senolytic activity [52,131,135]. When sulforaphane was co-administered alongside D + Q, the regimen extended this effect to the gut–brain axis, collectively attenuating neuroinflammation and restoring synaptic decline in obese female Wistar rats [64]. The gut microbiota exerts a fundamental role in transforming the pharmacokinetics of plant-derived senotherapeutics via biotransformation [147]. Since only 5 to 10% of ingested polyphenols are absorbed in the small intestine, the majority undergo microbial conversion in the colon into bioactive metabolites with greater bioavailability, including quercetin-derived hydroxyphenylacetic acids, curcumin-derived tetrahydrocurcumin, and ellagitannin-derived urolithins, each exhibiting superior pathway modulation relative to the parent compound [147,148]. The direct corollary is that interindividual variation in the gut microbiota is expected to produce corresponding variation in senotherapeutic efficacy at a given administered dose independent of pharmacogenomic variables. Hence, microbiome pharmacokinetics interaction should be incorporated into the design of in vivo and human trials of plant-derived senotherapeutics as a primary source of response heterogeneity and stratification by microbiome composition status.

### 3.7. Metabolomics-Guided Senolytic and Senomorphic Discovery: Current Status and Mechanistic Contributions

A central finding of this review is that metabolomics-guided studies, representing only 13.5% of included evidence, contribute a disproportionately rich mechanistic foundation. Metabolomics depends on advanced analytical platforms to identify compound scaffolds from complex matrices, resolve the complex metabolite profiles of in vitro and in vivo samples and pinpoint metabolic alterations within biological systems. In recent times, the principal techniques, including NMR, GC/MS, LC/MS, HPLC, capillary electrophoresis–tandem mass spectrometry (CE/MS), and ion-mobility spectrometry (IMS), offer complementary strengths for plant metabolomics. GC/MS is well suited to small, low-molecular-weight and volatile compounds, LC/MS provides high sensitivity with broad compound coverage, and CE/MS efficiently separates charged metabolites without derivatization; as well, IMS enhances metabolite identification through an additional ion-mobility dimension [149]. Furthermore, NMR is necessary for elucidation of compound structure and precise, reproducible quantification, particularly valuable for compound confirmation and for non-targeted metabolome profiling in biological matrices. In this review, the increasing adoption of untargeted or targeted metabolomics platforms in studies published after 2022 represents the most significant methodological shift in the natural senotherapy discovery pipeline and defines the direction toward future discovery and mechanistic validation strategies.

**Figure 3:**
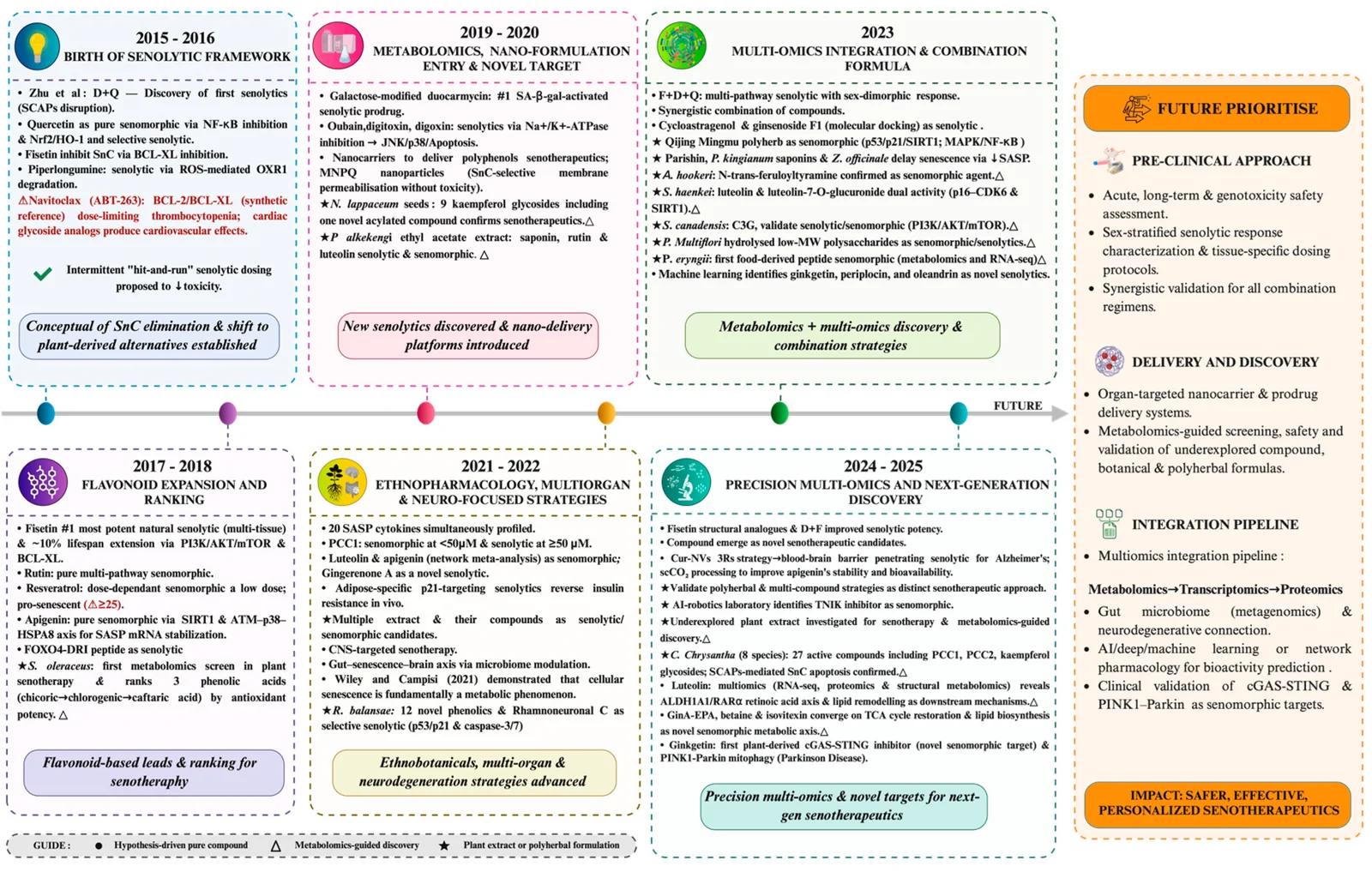
Timeline of plant-derived senolytic and senomorphic compound discovery spanning the post-senolytic era (2015–2025), illustrating the progressive transition from isolated flavonoid screening toward metabolomics and multi-omics integration, combinatorial formulation strategies, and next-generation discovery. Milestone discoveries and strategic advances are organized across six chronological phases and three parallel discovery paradigms are tracked throughout, including hypothesis-driven investigations of pure isolated compounds (●), metabolomics-guided or omics-assisted mechanistic discoveries (△), and plant extract or polyherbal formulation (★). Created in BioRender. Hussin, N. M. H. (2026). https://BioRender.com/yydt48q (accessed on 15 July 2026).

The majority of currently characterized natural senolytic and senomorphic compounds, including fisetin, quercetin, luteolin, resveratrol, and PCC1, were identified through hypothesis-driven, isolated compound screening. The timeline of plant-derived senotherapeutic discovery from 2015 to 2025 reveals a progressive methodological shift from hypothesis-driven compound screening toward metabolomics-guided and multi-omics integrated approaches (Figure 3). Traditional bioassay-guided fractionation is effective for abundant active constituents. However, this method requires iterative chromatographic separation steps before structural assignment can be made to any bioactive signal, entails substantial material loss across fractionation stages and is structurally incapable of detecting senotherapeutic effects arising from cooperative action among multiple co-occurring compounds [150,151]. By contrast, untargeted metabolomic platforms alone or integrated with molecular networking tool platforms circumvent these limitations by providing simultaneous chemical annotation across hundreds and thousands of metabolite features from a single extract. This enables compound identification before any fractionation step is undertaken [152,153,154]. ^1^H NMR metabolomics has been applied both as a discovery tool for constituent assignment [24,28,29] and as a serum biomarker platform for age-related metabolite validation in this review [153]. Given that bioactive secondary metabolites frequently occur at trace concentrations against a dense backdrop of co-extracted primary constituents, the sensitivity of these platforms is essential for rendering bioactive candidates analytically visible without prior enrichment.

An early proof-of-concept metabolomics senotherapy discovery was conducted by Ou et al. (2015), who applied metabolomics-informed extract characterization to *Sonchus oleraceus* L. (pūhā) leaf extracts tested against H_2_O_2_-induced SIPS in WI-38 human lung diploid fibroblasts [28]. Metabolite profiling identified caftaric acid, chlorogenic acid, and chicoric acid as the key active constituents, in which all caffeic acid derivatives share structural capacity for Nrf2 activation and direct radical scavenging. The extract reduced SA-β-Gal activity and improved cellular antioxidant capacity in a concentration-dependent manner, with the metabolite identification providing mechanistic rationale for the observed antioxidant-mediated senomorphic effects [28]. Lee et al. (2020) applied UPLC-qTOF-MS/MS/GNPS molecular networking to crude ethyl acetate and n-butanol fractions of *N. lappaceum* (rambutan) seeds, a tropical Southeast Asia fruit [29]. GNPS dereplication detected a novel acylated flavonol glycoside alongside nine known kaempferol-type flavonol glycosides. Subsequently, kaempferol 3-O-β-d-glucopyranosyl-7-O-α-l-rhamnopyranoside, kaempferol 3-O-rutinoside, and kaempferol 7-O-α-l-rhamnopyranoside at 10 µM were identified as contributors to senolytic and senomorphic effects in senescent HDFs across structurally related glycoside variants.

Application of ^1^H NMR metabolomics to *S. canadensis* (elderberry) extract confirmed cyanidin-3-glucoside (C3G) as the key bioactive anthocyanin responsible for senotherapeutics activity in HEK293T cells and Kunming mice [24]. Given the compositional complexity of elderberry extracts, which contain multiple co-eluting anthocyanins, acylated glycosides, and phenolic co-pigments, metabolomics confirmation of C3G was essential for attributing PI3K/AKT/mTOR pathway modulation, p16^INK4a^ and p21^CIP1/WAF1^ suppression, and antioxidant enzyme induction to a specific molecular scaffold. Similarly, the combination of NMR spectroscopy with HRESIMS was applied to *R. balansae* for characterization of 12 structurally novel phenolic compounds [27]. Of these, Rhamnoneuronal C demonstrated selective senolytic activity through suppression of p53/p21 and p16/p15 pathway markers, in addition to caspase-3/7-mediated SnC apoptosis, while Balansechromanones A and B achieved equivalent selectivity through a p53/p21-independent apoptotic mechanism. The identification of two senolytic sub-classes from a single botanical source illustrates that NMR-guided structural resolution can uncover not only novel compounds but also parallel mechanistic routes within a single extract.

In another study, Doan et al. (2023) applied metabolomics profiling to *Allium hookeri* leaf extracts and identified phenolamides as an active senomorphic constituent class [31]. Phenolamides—defined as hydroxycinnamic acid amides of polyamines, which had not previously been associated with senotherapy and their identification—underscore the discovery advantage conferred by untargeted metabolomics in terms of revealing novel lead compound classes that hypothesis-driven screening would likely miss. N-trans-feruloyltyramine was identified as the most promising SASP-inhibitory constituent within the phenolamide groups, sharing structural similarity to avenanthramide C and robustly downregulating senescence burden while attenuating SASP expression across two mechanistically distinct senescence induction models [31]. In contrast, *Camellia sect. Chrysantha* species identified 27 compounds and attributed the observed senolytic BCL-2 family protein downregulation to specific saponin-enriched fractions [30], which demonstrated that metabolomics could deconvolute multi-compound contributions within compositionally complex botanical matrices to assign bioactivity at the constituent level. Xu et al. (2025) further extended the paradigm beyond polyphenol-oriented discovery by focusing on carbohydrate-based bioactives [32]. This study demonstrates that structurally characterized low-molecular-weight polysaccharides from *Polygoni Multiflori Radix Praeparata* assessed by GC/MS exert dual senolytic and senomorphic activities in D-gal-senescent T lymphocytes and naturally aged mice. Similar metabolome to bioactivity mapping was performed for *A. membranaceus* root extracts, revealing that cycloastragenol and calycosin-7-glucoside were the dominant contributors to observed senolytics, telomerase activation, replicative senescence delay, and concurrent senomorphics [34]. This evidence showed that metabolomics can illuminate macromolecular senotherapeutics and can deconvolute multi-component contributions within a single botanical matrix.

Beyond compound discovery, metabolomics has been deployed as a mechanistic validation tool in several studies. Remodeling of lipid mediators was documented in at least three studies using structurally unrelated compounds [23,33,35]. In senescent vascular endothelial cells, luteolin reduced AA, S1P, and 9(S)-HODE while elevating pro-resolving mediators’ prostaglandin E2 (PGE2) and eicosapentaenoic acid (EPA), collectively shifting the intracellular lipid environment from pro-inflammatory to pro-resolution [23]. Isovitexin, as stated earlier, produced convergent fatty acid pathway remodeling, specifically reconfiguring fatty acid biosynthesis, linoleic acid, and alpha-linolenic acid metabolism to redirect eicosanoid and specialized pro-resolving mediator biosynthesis toward anti-inflammatory products [35]. GinA-EPA elevated mitochondrial membrane phospholipids, including phosphatidylethanolamine and phosphatidylglycerol detected by UPLC-QTOF-MS metabolomics of aged C57BL/6JN mice plasma and liver. This indicates that mitochondrial membrane lipid restoration is one of the components prone to senotherapeutics activity [33]. The convergence of lipid-mediator remodeling across three compound classes suggests that restoration of a pro-resolution lipid environment represents a conserved mechanistic feature of plant-derived senomorphic activity.

TCA cycle restoration emerged as a second consistent metabolic signature acting through anti-inflammatory mechanisms. *Pleurotus eryngii* bioactive peptides characterized through integrated RNA-seq and untargeted LC-MS/MS metabolomics restored TCA cycle function and enhanced pentose phosphate pathway (PPP) flux in D-gal-senescent PC12 neural cells, elevating NADPH to support glutathione recycling and antioxidant effect, with concurrent reduction in ROS by 51.53% and MDA by 27.11% [36]. Furthermore, GinA-EPA normalized TCA cycle metabolites and elevated hepatic NADP+ in aged mice [33]. Betaine (trimethylglycine) contributes carbon substrates to the TCA cycle and supports ATP synthesis via its sequential metabolic cascade (choline–betaine–dimethylglycine–glycine), where downstream metabolites are ultimately converted to pyruvate and acetyl-CoA. Critically, these compounds concurrently activate AMPK-mediated autophagy and FoxO/DAF-16 stress response pathways in *C. elegans*, with lifespan extension of 114% at 2 mM [37]. In the same study, age-dependent serum betaine depletion was validated in human subjects aged 23 to 77 years by ^1^H NMR metabolomics, further providing direct translational relevance to the in vivo findings. Across these studies, TCA cycle restoration consistently correlated with alleviation of oxidative senescence markers and SASP cytokines, suggesting central energy metabolism recovery as a senomorphic and antiaging intervention axis operating independently of the canonical signaling pathway. Consistent with a prior review, redox metabolomic profiling of replicatively senescent fibroblasts has uncovered systematic perturbations in intracellular metabolites that govern redox homeostasis [154]. However, metabolomics enabled studies of mechanistic precision by mapping specific upstream metabolite perturbations. In fact, metabolomics studies combined with in vitro antioxidant and anti-inflammatory bioassays have helped to reduce the time required for plant-based drug discovery by correlating the data from both methods. This resolution also has direct implications for understanding concentration-dependence and synergistic interactions in formulations.

Interestingly, studies also reported that metabolomics has been deployed as a tool to link compound treatments with specific metabolic pathway reconfiguration, providing crucial functional insights and evidence complementary to transcriptomic or proteomic data. Bai et al. (2025) demonstrated that a luteolin-treated senescent vascular endothelial cell metabolome delineates a specific lipid–retinoic acid axis with direct and separable contributions to p16/p21^CIP1^ regulation and SASP suppression [23]. Luteolin treatment elevated retinaldehyde dehydrogenase (RALDH; ALDH1A1/A2/A3 isoforms) activity in senescent vascular endothelial cells, restoring RA biosynthesis from retinaldehyde [23]. RALDH activity is characteristically suppressed in SnCs through oxidative inactivation of ALDH1 isoforms and NF-κB-mediated transcriptional repression, eventually resulting in an intracellular RA deficit. Luteolin-mediated RALDH restoration replenishes RA bioavailability, activating RA receptor-alpha (RARα). The RARα-retinoid X receptor (RXR) heterodimer complex bounds RA response elements within the p21^CIP1^ gene promoter and transactivated p21^CIP1^ transcription, reinforcing cell cycle arrest through CDK2 inhibition via the p53-NF-κB axis. Concurrently, RARα-RXR complexes suppress NF-κB by competing for shared CBP/p300 coactivators through ligand-dependent transrepression that directly attenuates SASP transcription, including IL-6, IL-8, MMP-1, MMP-3, and MCP-1, without requiring SnC clearance. In parallel, three lipotoxic metabolites (AA, S1P, and 9(S)-HODE) that independently amplify NF-κB-driven SASP were simultaneously reduced. Together, this multi-node convergence of SASP suppression without SnC clearance is consistent with a pure senomorphic classification and establishes the mechanistic precision that metabolomic pathway analysis provides beyond what protein or gene biomarker studies can resolve.

Despite these contributions, the application of metabolomics to natural senotherapy field is still at an early stage. The majority of extract-based studies in this review cannot distinguish constituent-level activity from matrix synergy without this analytical approach. For instance, extracts from *C. asiatica* exert a dual senomorphic and senolytic mechanism, yet the responsible constituents often remain uncharacterized, leaving unresolved whether the activity reflects a single major compound, additive contributions, or genuine synergy. Where deconvolution has been performed, such as the reduction of a *Salsola collina* extract to a three-component quercetin/β-sitosterol/salicylic-acid formulation that retains efficacy at approximately eight-fold lower total mass, the evidence supports constituent-level synergy rather than the dominance of a single compound. Such cases underscore that, for crude extracts, mechanistic claims engagement requires metabolomic constituent identification or profiling before they can be interpreted with the confidence achievable for isolated compounds. In fact, metabolites not only reflect the physiological and pathological activity of cells but can also directly influence cellular behavior, including the release of inflammatory cytokines that constitute the SASP [155]. Metabolomics can therefore interrogate how metabolic remodeling correlates with core senescence processes, including cell-cycle regulation, energy metabolism, and the oxidative-stress response, with the resulting maps of perturbed pathways exposing new therapeutic entry points, including molecules that reshape the metabolic profile to delay or reverse the senescent phenotype. The integration of metabolomics across the discovery to validation pipeline, from untargeted profiling to guided isolation and mechanistic metabolic mapping, is most powerfully demonstrated by the studies reviewed above, where each analytical step produced mechanistic information inaccessible to biomarker-level endpoints. The interesting point about integrated metabolomics is that any treatment-induced changes in the level of metabolites might reflect the up- or downregulation of the underlying biological processes [150]. Each of these perturbations can be directly related to the functioning of genes that are metabolite regulators since the metabolome represents the functional endpoint of gene expression. Remarkably, metabolomics is combined with complementary omics layers such as proteomics and genomics and is able to correlate metabolite changes with protein modifications and transcriptional regulation, resolve the molecular circuitry of senescence at a depth unattainable by any single platform and directly support the rational design of targeted interventions for age-related disease and the promotion of healthy aging (Table 5). Accordingly, adoption of systematic metabolomics integration as a minimum standard, encompassing standardized metabolite reporting using databases such as HMDB, KEGG, and METLIN [153], application of pathway enrichment analysis, and cross-study comparison of metabolite signatures, is recommended as a minimum standard for future botanical senotherapy investigations. Multi-omics integration offers the highest-resolution mechanistic framework currently available, and this framework is especially valuable, since it can link an individual bioactive constituent to the metabolic pathways it modulates and to the downstream transcriptional and proteomic changes that define its senolytic or senomorphic action.

### 3.8. Translational Challenges

Several interconnected challenges govern the translational trajectory of plant-derived senotherapeutics from preclinical proof-of-concept to human therapeutic application. Several studies explicitly addressed poor oral bioavailability and suboptimal dose as the primary barriers to clinical translation for most natural senotherapeutics. Quercetin, curcumin, fisetin, and resveratrol were the most extensively studied natural senotherapeutics in this review, which exhibit oral absorption below 10% due to limited aqueous solubility, extensive first-pass metabolism, and rapid phase II conjugation [156]. If the dosage from preclinical models is translated to humans, high oral doses (e.g., 1000–1250 mg quercetin) will be required to achieve therapeutic tissue concentrations, with attendant concerns about practicality, gastrointestinal tolerability and potential non-SnC toxicity at elevated systemic concentration. Conversely, polydatin demonstrates markedly higher intestinal absorption than resveratrol due to active intestinal glucose transporter-mediated uptake of the glucoside moiety prior to intestinal hydrolysis [69]. This can serve as a natural bioavailability-enhancement strategy within plant chemical space, illustrate that glycosylation of phenolic scaffolds warrants systematic pharmacokinetic evaluation as part of the lead optimization procedure. For crude plant extracts, this challenge is further compounded by the requirement for bioactive compound standardization and pharmacokinetic characterization. Effective in vitro doses reported across included studies, including 50–200 µg/mL for *C. asiatica* ethanol extract [126], 50–200 µg/mL for *S. marianum* flower extract [96,97], 10–200 µg/mL for *P. alkekengi* L. ethyl acetate fraction [25,26] and 5–60 µg/mL for *B. pandurata* ethanol extract [107], represent total extract concentrations at which the pharmacologically active fraction typically remains unquantified. An extract producing a senomorphic response at 100 µg/mL in vitro may contain its active constituent at 0.1 to 5 µg/mL, which means in vivo efficacy depends not on the total administered dose but on the achievable systemic concentration of the specific constituent following oral absorption, first-pass metabolism, and tissue distribution. The metabolomics-guided profiling represents methodological advances that directly address these issues by establishing constituent identity prior to pharmacokinetic modeling and enabling subsequent targeted pharmacokinetic studies of the relevant molecular species.

Functional food integration offers an attractive and alternative preventive pathway conferring advantages of enhanced acceptability, potential whole food matrix synergies, and applicability for health maintenance in aging populations without specific disease indications [13]. Evidence synthesized herein demonstrates comparable senotherapeutic bioactivity from complex botanical preparation relative to isolated compounds, potentially consistent with additive or synergistic mechanisms among co-occurring bioactive components through complementary molecular pathways. However, these applications confront substantial challenges, including bioactive content standardization, extraction/storage stability, accurate dosing, and efficacy validation at achievable dietary intakes. Addressing these requirements demands evidence-based senotherapeutic assessment by integration of innovative and advanced techniques incorporating multi-omic analytical platforms, particularly metabolomics combined with transcriptomics and proteomics, to elucidate bioactive mechanisms, identify synergistic interactions, and optimize formulation design for both pharmaceutical and functional food applications.

Another concern is plant toxicity due to the inherent toxicity of the combinations of compounds or microbial contamination that threatens human safety, which remains a critical consideration for translation. Accumulating evidence indicates that many traditional multi-herb formulations exhibit favorable safety margins, attributable in part to synergistic interactions that enhance therapeutic efficacy while mitigating the toxicity of individual constituents [142,157]. Comprehensive toxicological evaluations of polyherbal preparations have demonstrated high no-observed-adverse-effect levels (NOAELs) and absence of significant target organ toxicity in acute, sub-chronic, and chronic exposure studies, even at supra-therapeutic doses [142]. Nevertheless, herb–herb interactions (for example, *Glycyrrhiza pseudoaldosteronism* with long-term use, or CYP3A4 modulation by multiple flavonoid-containing herbs) and herb–drug interactions in geriatric populations represent genuine risks that must be systematically characterized [150]. In this context, structured toxicological evaluations, including repeated-dose, genotoxicity, and reproductive toxicity testing, are critical prior to clinical application, particularly in aging populations. For plant-derived senolytics specifically, the narrow therapeutic window between SnC-selective apoptosis and non-selective cytotoxicity in progenitor populations as identified across quercetin and *Camellia* extract necessitates thorough pharmacology safety studies in models that include non-senescent stem and progenitor cell populations as toxicological endpoints.

### 3.9. Future Research Directions

The advancement of targeting cellular senescence by natural senotherapeutics offers a promising approach for healthy aging and mitigating the burden of aging-related diseases. However, several critical aspects demand further research and refinement.

Strategic solutions include nanotechnology-based delivery systems, one of the most promising in terms of improving the bioavailability bottleneck while offering distinct advantages in terms of drug-loading capacity, controlled-release kinetics, and tissue-specific targeting. Diverse nanoformulation platforms have been investigated, including polymeric micelles, liposomes, mesoporous silica nanoparticles, dendrimers, hydrogels, and nanostructured lipid carriers [158]. Importantly, the therapeutic target for nanodelivering efforts should be defined prospectively, whereby future studies should aim to achieve tissue concentrations equivalent to the effective concentration required to eliminate 50% of SnCs (EC_50_) established in corresponding in vitro models. Quercetin-functionalized Fe_3_O_4_ magnetic nanoparticles (MNPQ) have demonstrated enhanced senolytic and AMPK-mediated senomorphic activity in H_2_O_2_-induced senescent fibroblasts at concentrations below those required for equivalent free-compound effects [42], while neutrophil-derived nanovesicle-encapsulated curcumin achieves blood–brain barrier penetration for CNS SnC modulation via active cellular trafficking [72]. Amorphous solid dispersion technology employing soluplus as a polymeric carrier achieved approximately 8000-fold solubility improvement for crystalline apigenin, substantially expanding its stability and oral bioavailability potential [159]. More recently, white adipose tissue-targeted liposomes co-delivering D + Q demonstrated superior SnC clearance in aged mice alongside concurrent reduction in hepatic lipid accumulation, which illustrate the dual potential of tissue-selective nanodelivery to enhance therapeutic efficacy while reducing systemic off-target exposure. Moreover, structural modification of parent compounds as demonstrated by quercetin derivatives and curcumin analogues offers an alternative complementary medicinal chemistry strategy to improve membrane permeability and metabolic stability while augmenting senotherapeutic potency [160].

Combination senotherapy strategies require more rigorous mechanistic and safety characterization before clinical advancement. Co-administration plant-derived compounds with pharmacokinetic enhancers (e.g., piperine with curcumin, fisetin or resveratrol) or with metabolism modifying co-agents (e.g., senomorphic or senolytic agents) offer a viable strategy for overcoming bioavailability hurdles and maximizing effectiveness. However, this approach introduces additional safety considerations, as pharmacokinetic and pharmacodynamic interactions may alter individual compound toxicity profiles in ways that require prospective characterization. The integration of senolytic and senomorphic agents with complementary mechanisms of action exemplified by D + Q and the sulforaphane-plus-D + Q regimen can yield compounded therapeutic benefits and extended senotherapeutic coverage. Expanding such combinations to include antioxidants or immunomodulatory agents requires careful optimization of dosing schedules and treatment duration; specifically, comparing intermittent high-dose senolytic protocols with continuous low-dose senomorphic regimens should be established as a primary endpoint, given that the pharmacodynamic rationale for administration timing differs substantially between these two mechanistic classes. Comprehensive absorption, distribution, metabolism, and excretion (ADME) interaction profiles assessing additive, synergistic, and antagonistic toxicological relationships are therefore essential prerequisites for safe clinical translation of multiagent natural senotherapeutic regimens.

The identification of senescence-modulating compounds from plant sources constitutes a high-yield but underutilized research avenue. Crude extracts, polyherbal combinations, and purified isolated bioactive compounds represent a vast reservoir of chemical diversity with potentially unique senescence modulatory mechanisms that have not yet been systematically interrogated. Recent discoveries of senolytic and senomorphic activity in constituents isolated from previously uncharacterized species [24,27,30,36] underscore the immense potential of integrating systematic bioassays and the metabolomics integrated screening pipeline with crude plant extracts. Studying the phytochemicals of medicinal plants using modern analytical techniques such as LC-MS, GC-MS, and NMR, etc., is the most precise, accurate, and robust methodology for qualitative- and quantitative-based standardization of medicinal plants, and such techniques are exponentially used to explore the metabolomics patterns of bioactive plant compounds for age-related diseases [149]. Accordingly, great attention should be given to issues of quality, efficacy and safety due to the intricate phytochemical matrix responsible for pharmacological effect. Dissecting these multi-component mixtures remains a fundamental analytical challenge, particularly when synergistic or antagonistic interactions among co-occurring constituents modify the overall pharmacological outcome. Indeed, exposure to xenobiotics and phytochemicals under specific physiological conditions may significantly alter the metabolome [150]; thus, comprehensive safety evaluations are essential. This assessment should encompass both systemic biological sampling and the target organ to sensitively detect potential metabolic disruption or unintended biological activity. Ultimately, metabolomics has enabled the effectiveness assessment of extract preparations and their safety, which constitute an important factors that must be tested in preclinical models before human use. Exploring these avenues could lead to the discovery of novel, non-invasive, and more effective senotherapeutics and anti-aging strategies.

Senescence heterogeneity across biological contexts and organ systems represents another critical frontier for the field that may require tailored research strategies. For in vitro studies, the top two models used were replicative senescence and SIPS, in which the ROS-producing oxidative stress most used in vitro is H_2_O_2_. The parameters and concentration used to induce senescence must be initially optimized individually and acquired to do dose-response tests according to each cell type for viability of SnCs but not cell death. In this review, 100–200 µM (especially 150 µM) was the most common dose included in growth media of cell cultures, whereas 5 mM H_2_O_2_ in embryo water has also been a part of practice. Hence, the recommended minimum design for future studies includes two complementary senescence models to allow both model-specific and -independent compound activity to be characterized. Moreover, small-sized animal models, particularly naturally aged mice at 20 to 24 months old, provide the most ideal model of plant-derived senotherapeutics in vivo. Such an approach is, however, time-consuming and may produce inconclusive results given the inherent heterogeneity of an aging phenotype [2]. Accordingly, accelerated- and premature-aging models offer practical alternatives, including genetically engineered progeroid strains that recapitulate features of human progeria syndromes, disease-specific models, and chemically or physically induced premature-aging models; for instance, D-gal (120–800 mg/kg/day, s.c. or i.p.), doxorubicin (5 mg/kg, i.p.), or ionizing radiation (6–10 Gy). In every case, the chosen model must be well matched to the specific experimental objective, since no single model captures the full spectrum of senescence biology.

In the absence of universally accepted gold-standard biomarkers for senescence, future studies should adopt a minimum outcome reporting panel incorporating SnC-selectivity assays (in vitro), SA-β-gal, p21, p16 and DDR marker expression and multiple secretomes, specifically IL-6, IL-8, and MCP1, as per the SASP Atlas suggested by Basisty et al. [6]. The current literature mostly addresses senescence in common cell and targeted tissues, such as skin, muscle, and adipose tissue that hinders tissue-specific senescence characterization. The roles of SnCs in complex systems like the cardiovascular system, central nervous system, and gastrointestinal tract demand particular attention, given their disproportionate impact on age-related morbidity. In addition, evaluating plant-derived senotherapeutics requires careful consideration of the bidirectional relationship between these compounds and the gut microbiome, which significantly influences both anti-aging efficacy and toxicity profiles with respect to their healthy aging efficacy and toxicity. This interplay underscores the critical need to optimize senotherapeutic delivery that maximizes direct interactions on gut microbiota. Consequently, future preclinical investigations should incorporate longitudinal metagenomic profiling at baseline and post-treatment to identify specific microbial taxa that mediate or predict therapeutic outcomes. Interaction of the microbiome with the pharmacokinetics of plant-derived senotherapeutics further introduces the interindividual variability that has not yet been systematically integrated into clinical translation frameworks and that requires specific attention [161]. Deciphering tissue-specific senescence biomarkers and their microenvironment is therefore fundamental to advancing precision aging therapies across diverse organ systems.

It is worth noting that achieving a comprehensive profiling of all compounds using a single platform is not possible given the complexity of physicochemical characteristics within biological systems. Therefore, combining NMR and MS techniques, in addition to the integration of metabolomics with other omics studies, remains indispensable for metabolomics to uncover the different mechanisms and the underlying pathways. Future research should also prioritize the complementary integration of metabolomics with other omics approaches, including transcriptomics, proteomics, and epigenomics, to fully characterize the mechanistic breadth and to identify robust molecular targets for precision therapeutic intervention. While metabolomics identifies what metabolite pools are perturbed, as demonstrated by the convergent TCA cycle restoration findings across GinA-EPA [33], betaine [37], and *Pleurotus eryngii* peptides [36], and the fatty acid biosynthesis remodeling identified via isovitexin [35] and luteolin [23], it cannot alone establish causal mechanistic direction of plant-derived senotherapeutics. For instance, LC-MS/MS metabolomics with transcriptomics (RNA-seq) within a single experimental design [23,36,37] enables the upstream gene regulatory context that explains why metabolite shifts occur by identifying differentially expressed genes in SASP-driving pathways (NF-κB, TGF-β, cGAS-STING) and the mitochondrial biogenesis mechanisms that account for it [36]. Proteomic analysis further validates the discoveries at the protein level, which is essential for senescence biomarkers regulated post-transcriptionally and for the full SASP secretomes that metabolomics cannot capture. Bioinformatic integration across these data should employ multi-omics factor analysis or weighted gene co-expression network analysis linked to metabolite set enrichment that translates raw metabolite-feature lists into mechanistically interpretable pathway signatures. Prospective implementation of molecular, metabolic, and protein expression pipeline evidence across senescence models treated with plant-derived candidates would shift mechanistic understanding from correlation to causation, enabling reproducible target validation and rational phytochemical-guided drug and functional food design.

#### Limitation

This scoping review has several limitations. First, only English-language articles were included, which may have excluded relevant studies published in other languages. Given that a substantial proportion of compound and ethnopharmacological research originates from regions where publication in the national language is common practice, the senotherapeutic potential of plant species prominent in non-Western medicinal traditions may not be captured. Second, this review relied exclusively on preclinical evidence (in vitro and in vivo animal studies), with no human clinical studies included. Although this focus enabled detailed mapping of molecular mechanisms, preclinical findings do not consistently translate to human physiology owing to species differences in genetics, disease pathways, and pharmacological responses. Accordingly, the results should therefore be interpreted as hypothesis-generating and require validation in human studies before clinical translation can be considered. Third, the review was restricted to publications between January 2015 and December 2025, which correspond to the post-senolytic discovery period. This temporal restriction necessarily excludes earlier foundational studies on plant-compound cytotoxicity against SnCs that predate formal senolytic terminology, and earlier mechanistic work on plant-derived senomorphic agents may similarly have been omitted. Finally, a proportion of the mechanistic relationships synthesized here derive from correlative biomarker changes rather than direct loss- or gain-of-function evidence, and attributions should therefore be regarded as provisional pending confirmation by gene knockdown or overexpression studies and integrated multi-omics approaches (Section 3.9).

## 4. Conclusions

This scoping review synthesized current evidence on plant-derived senotherapeutics, evaluating mechanisms of action through critical aging-related molecular pathways, therapeutic efficacy in diverse preclinical models and contribution of metabolomics-guided methodology to senotherapeutic discovery and mechanistic validation. Collectively, fisetin emerged as the most studied individual natural senolytic, followed by quercetin. Inhibition of the PI3K/AKT/mTOR pathway and suppression of p21/p16-mediated cell cycle arrest were identified as the most convergently targeted senolytic mechanisms, while NF-κB suppression and SASP modulation predominated among senomorphic agents. Ginkgetin-mediated inhibition of the cGAS-STING pathway was identified as a mechanistically novel target, reported here for the first time in a natural senotherapy review, warranting dedicated investigation in future studies. Convergent gut microbiome modulation across mechanistically distinct senolytic regimens further identifies the gut–senescence axis as an emerging and conserved feature of plant-derived senolytic activity. Methodological heterogeneity in compounds studied, senescence induction protocols, biomarker panels, and experimental models across studies constrains the generalizability of cross-study comparisons. Therefore, the adoption of standardized minimum reporting requirements and full dose-response characterization for preclinical senotherapeutic research represents an essential step toward enabling future systematic reviews and meta-analyses to generate clinically actionable comparative efficacy estimates across the growing body of evidence. Plant-derived senotherapeutics nonetheless hold translational promise by virtue of their pleiotropic pharmacological profiles, enabling simultaneous engagement of multiple senescence-driving pathways. Complementarily, metabolomics offers a rigorous and scalable methodological framework for characterizing and standardizing both compound profiles within complex plant matrices and pathway mapping following intervention. This capacity positions bioactive compounds as rational candidates for shifting conventional “one target, one drug, one disease” paradigms toward integrated, multitargeted modulation of the aging process. As global populations age, plant-derived senotherapeutics offer significant translational opportunities, not merely for lifespan extension but, more critically, for improving healthspan by integrating nutraceutical and ethnopharmacological knowledge with the precision tools of molecular gerontology.

## Figures and Tables

**Table 1:** PCC framework used to structure the search strategy and eligibility criteria for this scoping review.

**Population**	In vitro cellular models and in vivo animal models of cellular senescence.
**Concept**	Plant-derived senotherapeutic (senolytic and/or senomorphic activity) intervention.
**Context/Outcome**	Preclinical evidence of senescence modulation with documented biomarker and mechanistic assessment, including primary senescence biomarkers (SA-β-gal activity, p16^INK4a^ and p21^CIP1^ expression, cell cycle arrest) and secondary/SASP biomarkers (IL-6, IL-8, IL-1β, TNF-α, MMP-1, -3, -9, MMP-1, etc.), oxidative stress markers (ROS and MDA), as well as DNA damage markers and SnC-selectivity assays (Annexin V/P1, caspase-3/7 activity, TUNEL staining).

**Table 2:** Summary of characteristics of the 111 included studies organized by study design, publication period, senotherapeutic activity classification, compound class, and analytical discovery method.

Characteristics	Category	*n* (%)	Representative Examples
Study design	In vivo and whole-organism model	48 (43.2%)	Natural/chronological aging murine (C57BL/6, Sprague Dawley, SAMP8, 4–27 months) and progeroid genetic models (Ercc1^−/Δ^, TERT^−/−^). Induction method: D-gal (s.c., i.p.), doxorubicin (i.p.), HFD-induced metabolic senescence, specific disease-induced model (DSS-colitis, ApoE^−/−^ atherosclerosis, STZ-diabetes). Whole-organism model includes *C. elegans* and zebrafish (*Danio rerio*).
	In vitro	40 (36.0%)	Cell line: HaCaT, IMR-90 and WI-38 (human lung), BJ (human foreskin), HDFs, HUVECs, MSCs, MEFs, murine and primary preadipocytes, HAECs, BMSCs, hBMSCs SH-SY5Y (neuroblastoma), HT22 (hippocampal neuron), PC12 (neural) and BV2 (microglia), HEK293T, murine hepatocyte, cardiomyocyte, murine pre-osteoblast and chrondocyte, and others. Induction method: H_2_O_2_-induced SIPS; replicative exhaustion (serial passaging); genotoxic agents (bleomycin, etoposide, doxorubicin); ionizing radiation (UV-B); D-gal; OIS (RAS, BRAF).
	Combined in vitro + in vivo	23 (20.7%)	Parallel in vitro and in vivo models.
Publication period	2015–2020	28 (25.2%)	Early senolytic discovery: Q and fisetin.
	2021–2025	83 (74.8%)	Rapid expansion phase: discovery of novel agents, integration of transcriptomics and metabolomics, growth of crude extract and polyherbal formulation studies.
Senotherapeutic class	Senomorphic only	51 (45.9%)	Rutin, apigenin, EGCG, resveratrol, luteolin, ginkgetin, berberine, and ginsenoside Rg1.
	Dual (senolytic + senomorphic)	39 (35.1%)	Quercetin, fisetin, PCC1, cycloastragenol, Gingerenone A, *Camellia sps* extracts, and *Sonchus oleraceus.*
	Senolytic only	21 (18.9%)	Piperlongumine, cardiac glycosides (ouabain), *Rhamnoneuron balansae* extract.
	Dose-dependent	13 (11.7%)	PCC1 (senomorphic at <50 µM and senolytic at ≥50 µM), EGCG (senomorphic at 10–100 µM and senolytic at 50–100 µM), curcumin (dose-dependent senomorphic), resveratrol (senomorphic to *pro-senescent* at ≥25 µM), apigenin, C3G, FAE, kaempferol, eleutheroside E and sulforaphane.
Compound class	Flavonoids (flavonols, flavones, catechins, anthocyanins, isoflavones)	49 (44.1%)	Quercetin, fisetin, apigenin, EGCG, luteolin, rutin, kaempferol and its glycosides, isovitexin, and myricetin.
	Non-flavonoid polyphenols and stilbenes (phenolic acids, hydroxystilbenes, and diarylheptanoids)	23 (20.7%)	Resveratrol and polydatin, curcumin, chlorogenic acid and ginkgetin.
	Terpenoids, alkaloids, and nitrogen-containing derivatives (triterpenoids, saponins)	16 (14.4%)	Ginsenosides, berberine, cycloastragenol, astracaloside IV, parishin, berberine, rutaecarpine, indole-3-carbinol and ursolic acid
	Crude extracts and polyherbal formulations (multicomponent, fraction-containing preparations)	23 (20.7%)	*Silybum marianum*, *Allium hookeri*, *Nephelium lappaceum* L. seeds, *Centella asiatica*, *Panax ginseng Zingiber officinale* extract, and polyherbal (Qijing Mingmu decoction, JM10101).
Analytical discovery method	Metabolomics-guided (LC/MS, HPLC, GC/MS, NMR, etc.) and multi-omics	15 (13.5%)	[23,24,25,26,27,28,29,30,31,32,33,34,35,36,37].
	Hypothesis-driven compound	91 (82.0%)	[4,16,17,38,39,40,41,42,43,44,45,46,47,48,49,50,51,52,53,54,55,56,57,58,59,60,61,62,63,64,65,66,67,68,69,70,71,72,73,74,75,76,77,78,79,80,81,82,83,84,85,86,87,88,89,90,91,92,93,94,95,96,97,98,99,100,101,102,103,104,105,106,107,108,109,110,111,112,113,114,115,116,117,118,119,120,121,122,123,124,125].
	Bioassay-guided fractionation	5 (4.5%)	[126,127,128,129,130].

Abbreviations: HDFs, human dermal fibroblasts; HUVECs, human umbilical vein endothelial cells; MSCs, mesenchymal stem cells; H_2_O_2_, hydrogen peroxide; SIPS, stress-induced premature senescence; D-gal, D-galactose; IR, ionizing radiation; OIS, oncogene-induced senescence; HFD, high-fat diet; DSS, dextran sodium sulphate; STZ, streptozotocin; EGCG, epigallocatechin gallate; PCC1, procyanidin C1; Q, quercetin; LCMS/MS, liquid chromatography–tandem mass spectrometry; HPLC, high-performance liquid chromatography; NMR, nuclear magnetic resonance; SAMP8, senescence-accelerated mouse prone 8; Ercc1, excision repair cross-complementation group 1; TERT, telomerase reverse transcriptase; i.p., intraperitoneal.

**Table 3:** Frequency and primary senotherapeutic role of major signaling pathways targeted by plant-derived compounds.

Signaling Pathway	Studies (%)	Primary Role	Representative Compounds
PI3K/AKT/mTOR	39 (37.5%)	Senolytic	Quercetin, fisetin, cycloastragenol, EGCG, PCC1
NF-κB/SASP suppression	54 (51.9%)	Senomorphic	Quercetin, rutin, apigenin, curcumin, resveratrol, ginkgetin
p53/p21 pathway	47 (45.2%)	Both	Fisetin, quercetin, resveratrol, parishin, berberine, ginsenoside F1
Nrf2 antioxidant axis	33 (31.7%)	Senomorphic	Quercetin, EGCG, curcumin, luteolin, kaempferol, sulforaphane
SIRT1/AMPK	29 (27.9%)	Senomorphic	Resveratrol, anthocyanins, EGCG, delphinidin, parishin, kaempferol
BCL-2 family (SCAPs)	22 (21.2%)	Senolytic	Fisetin, PCC1, Cycloastragenol, *Camellia* sp. extracts
TGF-β/SMAD2	12 (11.5%)	Senomorphic	Apigenin + rutaecarpine, fisetin, rutin (indirect), red and black ginseng, *S. marianum* flower extract
p38 MAPK	11 (10.6%)	Senomorphic	Apigenin, luteolin, magnolin, fisetin (VSMC), kaempferol, *Z. officinale*, Qijing Mingmu decoction
PINK1-Parkin mitophagy	5 (4.8%)	Senomorphic	Astragaloside IV, hesperetin, curcumin
cGAS-STING	2 (1.9%)	Senomorphic	Ginkgetin (direct STING CTD binding)

Abbreviations: PCC1, procyanidin C1; EGCG, epigallocatechin gallate; SCAPs, senescent cell anti-apoptotic pathways; cGAS-STING, cyclic GMP-AMP synthase–stimulator of interferon genes; PI3K, phosphoinositide 3-kinase; AKT, protein kinase B; mTOR, mechanistic target of rapamycin; NF-κB, nuclear factor kappa B; SASP, senescence-associated secretory phenotype; Nrf2, nuclear factor erythroid 2-related factor 2; SIRT1, sirtuin 1; AMPK, AMP-activated protein kinase; BCL-2, B-cell lymphoma 2; BCL-XL, B-cell lymphoma extra-large; SCAPs, senescent cell anti-apoptotic pathways; TGF-β, transforming growth factor beta; SMAD2, SMAD family member 2; p38 MAPK, p38 mitogen-activated protein kinase; PINK1, PTEN-induced kinase 1; cGAS, cyclic GMP-AMP synthase; STING, stimulator of interferon genes; CTD, C-terminal domain; IRF3, interferon regulatory factor 3; PCC1, procyanidin C1; EGCG, epigallocatechin gallate; VSMCs, vascular smooth muscle cells.

**Table 4:** Compound class, principal molecular targets, and predominant senotherapeutic outcome of plant-derived agents identified in this review.

Compound Class	Representative Compounds	Principal Molecular Target(s)	Predominant Outcome
Flavonols	Quercetin, fisetin, kaempferol, myricetin	PI3K/AKT/mTOR → BCL-2/BCL-XL (indirect); p53/p21; Nrf2; NF-κB	Senolytic (high dose)/Senomorphic (low dose); dual
Flavones	Apigenin, luteolin	NF-κB via IL-1α/IRAK1-4/p38 MAPK; SIRT1–p53; Nrf2/HO-1; MAPK/AP-1; RALDH–retinoic acid (luteolin)	Senomorphic
Flavan-3-ols/catechins	EGCG, PCC1	NF-κB; PI3K/AKT/mTOR; ROS–mitochondrial apoptosis (PCC1 ≥ 50 µM); Nrf2/SIRT3	Dose-dependent dual
Flavonoid glycosides/biflavonoids	Rutin, ginkgetin	Rutin: ATM/HIF-1α → NF-κB; Ginkgetin: direct STING-CTD binding → cGAS-STING inhibition	Senomorphic
Anthocyanins	Cyanidin-3-glucoside, delphinidin	PI3K/AKT/mTOR → p53/p21 and p16/Rb (C3G); NF-κB; AMPK/SIRT1 (delphinidin)	Senomorphic (C3G dual)
Stilbenes	Resveratrol, polydatin	SIRT1 (direct + AMPK); Nrf2/Keap1 (polydatin)	Senomorphic (resveratrol pro-senescent ≥ 25 µM)
Curcuminoids	Curcumin	NF-κB; Nrf2; SIRT1/AMPK/mTOR → autophagy	Senomorphic
Phenolic acids	Caffeic, chlorogenic, rosmarinic acid	NF-κB suppression; Nrf2 antioxidant axis	Senomorphic
Triterpenoids/saponins	Cycloastragenol, astragaloside IV, ginsenosides Rg1/F1, α/β-amyrins	BCL-2 + PI3K/AKT/mTOR (cycloastragenol); PINK1 mitophagy (astragaloside IV); TLR2/MyD88/NF-κB (Rg1); TRF2 telomere (F1); CB2R (amyrins)	Mixed: senolytic (cycloastragenol, amyrins)/senomorphic (Rg1, astragaloside IV)
Alkaloids/amides	Berberine, piperlongumine, rutaecarpine, indole-3-carbinol, phenolamides	Berberine: p16/CDK4/RB + mTOR; piperlongumine: ROS → OXR1 degradation; I3C: BCL-2 inhibition; rutaecarpine: TGF-β/SMAD2; phenolamides: NF-κB	Mixed: senolytic (piperlongumine, I3C)/senomorphic (berberine, rutaecarpine)
Crude extracts and polyherbal formulations	*S. marianum*, *C. asiatica*, *Z. officinale*, *A. membranaceus*, FAE, JM10101, Qijing Mingmu decoction, SBT	Multi-pathway: NF-κB, p53/p21, PI3K/AKT, BCL-2 family, SIRT1 engaged	Variable/both (constituent-dependent)

Abbreviations: SCAP, senescent-cell anti-apoptotic pathway; RALDH, retinaldehyde dehydrogenase; CTD, C-terminal domain; CB2R, cannabinoid receptor 2; FAE, *Physalis alkekengi* extract. Outcome reflects the predominant activity reported and several agents switch mode with concentration (see Supplementary Table S2).

**Table 5:** Studies applying metabolomics or integrating multi-omics-guided workflows to the discovery and/or mechanistic validation of plant-derived senotherapeutics (*n* = 15; 13.5% of included studies). The table shows the analytical platform, the bioactive constituent(s) resolved, and the pathway validated, distinguishing discovery applications (identifying the active molecule from a complex matrix) from mechanistic-validation applications (mapping downstream metabolic remodeling).

Study	Plant Source/Compound	Platform	Bioactive(s) Identified	Pathway Validated	Class
[24]	*S. canadensis* (elderberry)	^1^H NMR (discovery)	Cyanidin-3-glucoside (C3G)	PI3K/AKT/mTOR → p53/p21 and p16/Rb	Dual
[27]	*R. balansae*	NMR + HRESIMS (discovery)	Rhamnoneuronal C; Balansechromanones A/B (12 novel phenolics)	p53/p21, p16/p15, and caspase-3/7 apoptosis	Senolytic
[28]	*S. oleraceus*	Metabolite profiling (discovery)	Caftaric, chlorogenic and chicoric acids	Nrf2 activation and radical scavenging capacities	Senomorphic
[29]	*N. lappaceum* seeds	UPLC-qTOF-MS/MS + GNPS networking (discovery)	Kaempferol-type flavonol glycosides (+novel acylated glycoside)	p53/p21 arrest and SIRT1 activation	Dual senolytic/senomorphic
[30]	*Camellia* sect. *Chrysantha*	Metabolite profiling (discovery)	27 compounds and saponin	BCL-2 family downregulation	Senolytic
[31]	*A. hookeri*	UHPLC-qTOF-MS/MS (discovery)	Phenolamides (N-trans-feruloyltyramine)	NF-κB/SASP suppression	Senomorphic
[32]	*Polygoni Multiflori Radix Praeparata*	Gas chromatography–mass Spectrometry (GC/MS) for polysaccharide characterization (discovery)	Low-MW polysaccharides	Nrf2-activation	Dual senolytic/senomorphic
[34]	*A. membranaceus*	Metabolome-to-bioactivity mapping (discovery)	Cycloastragenol and calycosin-7-glucoside	BCL-2/PI3K/AKT and telomerase activation	Dual senolytic/senomorphic
[25]	*P. alkekengi* (FAE fruit)	Metabolomics (discovery)	Luteolin, rutin and saponins	SnC clearance and antioxidant	Dual senolytic/senomorphic
[26]	*P. alkekengi* (ethyl acetate)	Metabolomics (discovery)	Luteolin glucosides and physalins	NF-κB suppression	Senomorphic
[23]	Luteolin	Untargeted LC-MS/MS + multi-omics (validation)	Lipid–retinoic-acid axis	RALDH → RA → RARα/RXR, ↓ AA/S1P/9(S)-HODE, and NF-κB	Senomorphic
[35]	Isovitexin	Untargeted LC-MS/MS (validation)	Fatty acid pathway remodeling	Fatty acid biosynthesis, linoleic/α-linolenic; PI3K/AKT, and Nrf2/NF-κB	Senomorphic
[33]	Gingerenone A/GinA-EPA	UPLC-QTOF-MS + RNA-seq (validation)	Mitochondrial phospholipid restoration	AMPK/SIRT1, TCA-cycle normalization and membrane remodeling	Dual
[36]	*Pleurotus eryngii* peptides	Integrated RNA-seq + untargeted LC-MS/MS (validation)	TCA + pentose-phosphate restoration	NADPH/glutathione recycling and ROS ↓ 51.5%	Senomorphic
[37]	Betaine (+quercetin)	^1^H NMR (validation)	Choline–betaine–TCA cascade	TCA carbon/ATP, AMPK autophagy and FoxO/DAF-16	Senomorphic/anti-aging

Abbreviations: GNPS, Global Natural Products Social Molecular Networking; HRESIMS, high-resolution electrospray–ionization mass spectrometry; RALDH, retinaldehyde dehydrogenase; RA, retinoic acid; AA, arachidonic acid; S1P, sphingosine-1-phosphate; 9(S)-HODE, 9(S)-hydroxyoctadecadienoic acid; TCA, tricarboxylic acid. ↓ indicates reduction.

## Data Availability

No new data were created and analyzed in this review paper. Data sharing is not applicable to this article.

## Supplementary Materials

The following supporting information can be downloaded at: https://www.mdpi.com/article/10.3390/ijms27167181/s1.

## Abbreviations

The following abbreviations are used in this manuscript:

ABT-263	navitoclax
ADME	absorption, distribution, metabolism, and excretion
AKT	protein kinase B
AMPK	AMP-activated protein kinase
ARE	antioxidant response element
ATM	ataxia-telangiectasia mutated kinase
BBB	blood–brain barrier
BCL-2	B-cell lymphoma 2
BCL-XL	B-cell lymphoma-extra-large
CAT	catalase
CCL2	C-C motif chemokine ligand 2
CDK4	cyclin-dependent kinase 4
CDK6	cyclin-dependent kinase 6
cGAS-STING	cyclic GMP-AMP synthase–stimulator of interferon genes
COX-2	cyclooxygenase-2
CXCL1	C-X-C motif chemokine ligand 1
CXCL8	C-X-C motif chemokine ligand 8
D + Q	dasatinib and quercetin
DSS	dextran sulfate sodium
EGCG	epigallocatechin gallate
FAE	Physalis alkekengi ethyl acetate fraction
FOXO	forkhead box O
GDF-15	growth differentiation factor 15
GM-CSF	granulocyte-macrophage colony-stimulating factor
GNPS	Global natural products social molecular networking
GSH	reduced glutathione
GSSG	oxidized glutathione disulfide
H_2_O_2_	hydrogen peroxide
HDFs	human dermal fibroblasts
HFD	high-fat diet
HIF-1α	hypoxia-inducible factor-1 alpha
HO-1	heme oxygenase-1
hTERT	human telomerase reverse transcriptase
HUVECs	human umbilical vein endothelial cells
I3C	indole-3-carbinol
ICAM-1	intercellular adhesion molecule-1
IKK	IκB kinase
IL-1α	interleukin-1 alpha
IL-1β	interleukin-1 beta
IL-6	interleukin-6
IL-8	interleukin-8
iNOS	inducible nitric oxide synthase
IP-10	interferon gamma-induced protein 10
IR	ionizing radiation
IRAK1	interleukin-1 receptor-associated kinase 1
IRAK4	interleukin-1 receptor-associated kinase 4
JBI	Joanna Briggs Institute
LC-MS/MS	liquid chromatography–tandem mass spectrometry
MAPK	mitogen-activated protein kinase
MCP-1	monocyte chemoattractant protein-1
MDA	malondialdehyde
MMP	matrix metalloproteinase
MNPQ	quercetin-functionalized Fe_3_O_4_ magnetic nanoparticles
MSCs	mesenchymal stem cells
mTOR	mechanistic target of rapamycin
NAD^+^	nicotinamide adenine dinucleotide
NF-κB	nuclear factor kappa B
NOAEL	no-observed-adverse-effect level
Nrf2	nuclear factor erythroid 2-related factor 2
p16^INHA^	cyclin-dependent kinase inhibitor 2A
p21^CID1^/WA^f1^	cyclin-dependent kinase inhibitor 1A
p38 MAPK	p38 mitogen-activated protein kinase
p53	tumor protein 53
PAI-1	plasminogen activator inhibitor-1
PAMP	pathogen-associated molecular pattern
PCC	Population, concept, and context
PCC1	procyanidin C1
PGC-1α	peroxisome proliferator-activated receptor gamma coactivator 1-alpha
PI3K	phosphoinositide 3-kinase
PRISMA-ScR	Preferred Reporting Items for Systematic Reviews and Meta-Analyses Extension for Scoping Reviews
RB	retinoblastoma protein
ROS	reactive oxygen species
SA-β-gal	senescence-associated β-galactosidase
SAMP8	senescence-accelerated mouse prone 8
SASP	senescence-associated secretory phenotype
SCAPs	senescent cell anti-apoptotic pathways
SIPS	stress-induced premature senescence
SIRT1	sirtuin 1
SIRT3	sirtuin 3
SnC	senescent cell
SOD	superoxide dismutase
SYRCLE	Systematic Review Centre for Laboratory Animal Experimentation
TGF-β	transforming growth factor beta
TIMP	tissue inhibitor of metalloproteinase
TLR2	toll-like receptor 2
TNF-α	tumor necrosis factor alpha
TRF2	telomeric repeat-binding factor 2
UHPLC-qTOF-MS/MS	ultra-high-performance liquid chromatography–quadrupole time-of-flight mass spectrometry
UPLC-qTOF-MS/MS	ultra-performance liquid chromatography–quadrupole time-of-flight mass spectrometry
VSMCs	vascular smooth muscle cells
γH2AX	phosphorylated histone H2AX
