# Supplementary material for: Plant-Derived Senotherapeutics in Cellular Senescence: A Scoping Review of Preclinical Evidence, Mechanistic Pathways, and Metabolomic-Guided Discovery
Source: Int J Mol Sci. 2026 Aug 11;27(16):7181. doi: 10.3390/ijms27167181 (PMC13513267; doi:10.3390/ijms27167181)
Supplement: Supplementary file 1 [file ijms-27-07181-s001.zip › ijms-4351210-Table S3.pdf]

To ensure reproducibility, this Boolean logic was adapted to the syntax of each database including MeSH terms combined with title/abstract fields in PubMed, TITLE-ABS-KEY in Scopus, the Topic (TS) field in Web of Science, and the 'All Fields' option in the Wiley Online Library (Supplementary Table S3).

Table S3. Full database-specific search strings used to identify studies on plant-derived senotherapeutics. A two-concept Boolean structure (senescence / senotherapeutic activity AND plant-derived agents) was applied and adapted to the native syntax of each database. All searches were executed in January 2026.

Database (Platform)	Full search string	Limits / filters applied
**PubMed** (NLM) (MeSH + Title/Abstract)	(("Cellular Senescence"[MeSH Terms] OR ("aging"[MeSH Terms] OR "aging"[All Fields] OR "senescence"[All Fields] OR "senesce"[All Fields] OR "senesced"[All Fields] OR "senescences"[All Fields] OR "senescent"[All Fields] OR "senescents"[All Fields] OR "senesces"[All Fields] OR "senescing"[All Fields]) OR ("aging"[MeSH Terms] OR "aging"[All Fields] OR "senescence"[All Fields] OR "senesce"[All Fields] OR "senesced"[All Fields] OR "senescences"[All Fields] OR "senescent"[All Fields] OR "senescents"[All Fields] OR "senesces"[All Fields] OR "senescing"[All Fields]) OR "Senescence-Associated Secretory Phenotype"[MeSH Terms] OR "cellular senescence*"[Title/Abstract] OR "senescence associated secretory phenotype*"[Title/Abstract] OR "sasp*"[Title/Abstract]) AND ("2015/12/02 00:00":"3000/01/01 05:00"[Date - Publication] AND "animals"[MeSH Terms:noexp] AND "english"[Language]) AND (("Senotherapeutics"[MeSH Terms] OR "Geroscience"[MeSH Terms] OR "senotherapeutic*"[Title/Abstract] OR "senolytic*"[Title/Abstract] OR "senomorphic*"[Title/Abstract] OR "geroscience*"[Title/Abstract]) AND ("2015/12/02 00:00":"3000/01/01 05:00"[Date - Publication] AND "animals"[MeSH Terms:noexp] AND "english"[Language])) AND (("Phytochemicals"[MeSH Terms] OR "Plant Extracts"[MeSH Terms] OR "phytochemical*"[Title/Abstract] OR "plant extract*"[Title/Abstract] OR "polyphenol*"[Title/Abstract] OR "flavonoid*"[Title/Abstract] OR "terpenoid*"[All Fields] OR "alkaloid*"[All Fields] OR "saponin*"[All Fields] OR (("medicin"[All Fields] OR "medicinal"[All Fields] OR "medicinally"[All Fields] OR "medicinals"[All Fields] OR "medicine"[MeSH Terms] OR "medicine"[All Fields] OR "medicine s"[All Fields] OR medicines"[All Fields]) AND "plant*"[All Fields]) OR ("quercetin"[Supplementary Concept] OR "quercetin"[All Fields] OR "quercetin"[MeSH Terms] OR "quercetin s"[All Fields] OR "quercetine"[All Fields] OR "quercetins"[All Fields]) OR ("resveratrol"[Supplementary Concept] OR "resveratrol"[All Fields] OR "resveratrol"[MeSH Terms] OR "resveratrol s"[All Fields] OR "resveratrols"[All Fields]) OR ("curcumin"[Supplementary Concept] OR "curcumin"[All Fields] OR "curcumin"[MeSH Terms] OR "curcumin s"[All Fields] OR "curcumine"[All Fields] OR "curcumins"[All Fields]) OR ("fisetin"[Supplementary Concept] OR "fisetin"[All Fields]) OR ("luteolin"[Supplementary Concept] OR "luteolin"[All Fields] OR "luteolin"[MeSH Terms] OR "luteoline"[All Fields] OR "luteolins"[All Fields]) OR ("kaempferol"[Supplementary Concept] OR "kaempferol"[All Fields] OR "kaempferols"[MeSH Terms] OR "kaempferols"[All Fields]) OR "EGCG"[All Fields] OR "ginsenoside*"[All Fields] OR ("icariin"[Supplementary Concept] OR "icariin"[All Fields] OR "icariine"[All Fields]) OR ("berberin"[All Fields] OR "berberine"[Supplementary Concept] OR "berberine"[All Fields] OR " erberine"[MeSH Terms] OR "berberine s"[All Fields] OR "berberines"[All Fields]) OR "flavonoid*"[All Fields])	Publication date 2015–2025; Species =cell culture, in vitro, animals, in vivo [MeSH]; Language = English (filters embedded in the query).

**Web of Science** (Core Collection, Clarivate) Topic (TS) field	((ALL=("senescence" OR "cellular senescence" OR "senolytic" OR "senotherapeutic" OR "senomorphic" OR "senescence-targeted therapies" OR "senescent cell*" OR SASP)) AND ALL=("natural products" OR "plant-derived compounds" OR "plant bioactive compound" OR "herbal extracts" OR "phytochemicals" OR "natural senolytics" OR "botanical extracts" OR "phytoconstituents" OR "natural compound" OR polyphenol* OR flavonoid* OR "herbal medicine" OR "botanical extract*"))	Publication Years 2015–2025; Document Type = Article; Language = English
**Scopus** (Elsevier) (TITLE-ABS-KEY)	("natural product*" OR "herbal medicine" OR "botanical extract*" OR "polyherbal" OR "medicinal plant*" OR "natural compounds" OR "plant bioactive compound" OR "phytochemicals" OR "natural senolytics") AND (senolytic* OR senomorphic* OR "cellular senescence" OR sasp) AND (mechanism OR pathway OR "mode of action" OR inhibition OR activation)	Publication years 2015–2025; Document type = Article; Language = English
**Wiley Online Library** (Advanced Search) (All Field)	("cellular senescence" OR "senescent cells" OR SASP) AND (senolytic* OR senomorphic* OR "anti-aging") AND ("natural product*" OR polyphenol* OR flavonoid* OR "herbal medicine" OR "botanical extract") AND (mechanism OR pathway OR apoptosis OR autophagy)	Date range 2015–2025; article / research-type; English.
**Google Scholar and References checking** (Supplementary / grey literature)	("senolytics" OR "senotherapeutics" OR "senomorphics" OR "senescence-targeted therapies" OR "cellular senescence" OR "SASP")<br>AND ("natural products" OR "plant-derived compounds" OR "plant bioactive compounds" OR "herbal extracts" OR "phytochemicals" OR "natural senolytics" OR "phytoconstituent" OR "natural compound" OR "botanical extracts" OR "medicinal plants" OR "phytotherapeutics")<br>AND "research article"	Custom range 2015–2025; used to supplement grey literature and cross-check references.
