# Supplementary material for: Plant-Derived Senotherapeutics in Cellular Senescence: A Scoping Review of Preclinical Evidence, Mechanistic Pathways, and Metabolomic-Guided Discovery
Source: Int J Mol Sci. 2026 Aug 11;27(16):7181. doi: 10.3390/ijms27167181 (PMC13513267; doi:10.3390/ijms27167181)
Supplement: Supplementary file 1 [file ijms-27-07181-s001.zip › ijms-4351210-Table S2.pdf]

Table S2. Summary of studies related to plant-derived senotherapeutics and related mechanisms (2015-2025)

Author & Year	Compound/Extract	Study Type & Details	Key Senescence Biomarkers (Treatment Effect)	Mechanism & Pathway	Senotherapeutic Classification
[21]	Quercetin	In vitro model: 3T3-L1 pre-adipocytes. Senescence inducer: Stress-Induced Premature Senescence (SIPS), 3 consecutive days of H_2O_2 150 μM × 3 days, 3h/day. Dose Q: 0-100 μM	SA-β-gal ↓; p21 ↓; IL-6 ↓; ROS ↓; MCP-1 ↓; miR-155-5p ↓	Nrf2 pathway activation; NF-κB pathway inhibition); silent information regulator 1 (SIRT1) upregulation; p53/p21 pathway modulation; apoptotic induction	Dual (Senolytic + Senomorphic)
[22]	Quercetin	In Vitro: Werner Syndrome hMSCs, Hutchinson-Gilford Progeria Syndrome (HGPS). Model: replicative-senescence. In Vivo: Mouse tibialis anterior muscle implantation, NOD SCID mice fat pad transplantation. Dose Q: NR	SA-β-gal ↓; p21 ↓; IL-6 ↓, ROS ↓; Ki67 ↑; Telomere length ↑; γ-H2AX ↓; BCL-2 ↓; AKT ↓; Lamin B1 ↑	PI3K/AKT pathway inhibition; Nrf2 pathway; Apoptosis induction; Anti-inflammatory	Dual (Senolytic + Senomorphic)
[23]	Quercetin	In vivo. Male C57BL/6J high fat diet (HFD)-induced obesity/ hypercholesterolemia. Dose quercetin: 50 mg/kg oral gavage, 5 days biweekly × 10 weeks.	SA-β-gal ↓; p16 ↓; p21 ↓; p53 ↓; IL-6 ↓; TNF-α ↓	Nrf2 pathway activation; Apoptosis induction	Senomorphic
[24]	Quercetin surface functionalized Fe_3O_4 nanoparticles (MNPQ)	In vitro: Human foreskin fibroblasts (BJ), Maximum population doubling level= 72. Senescence inducer: SIPS: H_2O_2 100 μM × 2h. Dose Q: 0-100 μM.	SA-β-gal ↓; p16 ↓; p21 ↓; ROS↓; SASP ↓; Apoptosis ↑ (SnC selective); Cell membrane permeability ↑; Cell viability ↓ (SnC)	AMPK pathway activation; NF-κB pathway inhibition; PI3K/AKT pathway inhibition; Apoptosis induction	Dual (Senolytic + Senomorphic)
[25]	Quercetin	In vitro: VSMCs isolated from thoracic aorta of Sprague-Dawley rats. Senescence inducer: H_2O_2 50 μM (selected from dose-response: 0-400 μM) × 1 h single exposure.	SA-β-gal ↓; p16 ↓; p21 ↓; p53 ↓; ROS ↓; BCL-2 ↓; BAX ↑; Cytochrome C ↑; Caspase-3 ↑; Cleaved caspase-3 ↑	AMPK pathway activation; p53/p21 pathway modulation; Bcl-2 family modulation; Mitochondrial apoptosis pathway activation; Apoptosis induction	Senolytic
[14]	Quercetin	In vitro: IL-1β-induced senescence in human nucleus pulposus cells (NPCs). Dose Q: 0, 10, and 20 μM (tested up to 100 μM for toxicity). In vivo: Puncture-induced intervertebral disc degeneration in adult male Sprague-Dawley rats. Dose: 100 mg/kg, intragastric, daily for 4 weeks.	SA-β-gal ↓; p16 ↓; p21 ↓; SASP ↓; IL-6 ↓	NF-κB pathway inhibition; Nrf2 pathway activation	Senomorphic
[13]	Quercetin	In vitro: H_2O_2-induced senescence human bone marrow mesenchymal stem cells (BMSCs). Optimal dose: 1 μM (range 1- 20 μM). In vivo: puncture-induced intervertebral disc (IVD)	SA-β-gal ↓; p16 ↓; p21 ↓; SASP ↓ (MMP-3, MMP-13, IL-6, IL-8)	NF-κB pathway inhibition; Nrf2 pathway activation	Senomorphic

Author & Year	Compound/Extract	Study Type & Details	Key Senescence Biomarkers (Treatment Effect)	Mechanism & Pathway	Senotherapeutic Classification
		degeneration in male Sprague-Dawley rats. Dose Q: 100 mg/kg intragastric daily × 4 weeks.			
[26]	Quercetin	In vivo rat myoblast cell line H9c2 (non-contractile cardiomyocyte phenotype). Senescence inducer: 40 g/L D-gal × 48 h. Optimal concentration Q: 6.5 mg/L (optimal)	SA-β-gal ↓; p16 ↓; p21 ↓; IL-1β ↓; ROS; SASP ↓ (IL-1β, MCP-1, and CXCL-1). Cell viability ↓ (SnC): ROS ↓	p53/p21 pathway; anti-SASP; ROS reduction	Dual (Senolytic + Senomorphic)
[27]	Polydatin + Curcumin + Quercetin (combination & individual)	In vitro: Young and senescent Human Endothelial Cells (HUVECs); Snescnce inducer: high glucose (D-Glucose 45 mM). Dose: Polydatin 10 µM; Curcumin 1 µM; Quercetin 0.5 µM; MIX (combination) 10 µM polydatin+ 1 µM Curcumin + 0.5 µM quercetin	SA-β-gal ↓; p16 ↓; SASP (IL-6) ↓; IL-1β ↓; ROS↓	NF-κB pathway; Anti-inflammatory; synergistic senomorphic activity (MIX > individual compound)	Senomorphic
[15]	Orthosilicic acid + Vit K2 + Curcumin: + Polydatin + Quercetin (combination & individual)	In vitro: Young and senescent human bone marrow mesenchymal stromal cells (MSCs). Model: replicative senescence. Dose: Orthosilicic acid 75 µM, Vit K2 0.1 µM, Curcumin 1 µM, Polydatin 10 µM, Quercetin 0.5 µM	SA-β-gal ↓; p16 ↓; p21↓; SASP ↓ (IL-1β, IL-6, IL-8, IL-1β, MCP-1 (mRNA expression and protein secretion), miR-21, and miR-146a (inflammaging-associated microRNAs)), Cell viability ↓ (SnC)	NF-κB pathway; Synergistic anti-inflammatory activity (↓ Inflammaging-combined effects (MIX 5 compounds); Enhanced osteogenic differentiation	Senomorphic
[28]	Rutin	In vitro: Prostate stromal cells (PSC27), WI38, IMR90. Senescence inducer: Bleomycin 50 µg/mL. Dose rutin: 100 µM. In vivo: NOD/SCID male mice. Dose rutin: 10 mg/kg intraperitoneal, weekly.	SA-β-gal ↔ ; p16 ↔; p21 ↔; IL-6 ↓; IL-8 ↓; IL-1α ↓; IL-1β ↓; CXCL3 ↓; MMP-3 ↓; GM-CSF ↓	ATM/HIF1α pathway inhibition; NF-κB pathway inhibition; Antioxidant; Pure senomorphic (no SnC elimination)	Senomorphic
[29]	Fisetin	In vivo: C57BL/6N mice (male young 6–8 mo; old 27 mo male, 29 mo female); p16-3MR mice (male and female). Comparators: genetic SnC clearance, ABT-263. Dose: fisetin 50 mg/kg/day oral gavage, intermittent	$p16^{INK4A}$ ↓; $p21^{CIP1}$ ↓; Plasminogen activator inhibitor-1 (PAI-1) ↓; Lamin B1 ↑; SASP ↓; DNA damage and Cell cycle arrest markers	PI3K/AKT pathway inhibition; mTOR pathway inhibition; Apoptosis induction; SnC clearance from skeletal muscle myonuclei, pericytes, fibro-adipogenic progenitors, neural cells;	Senolytic
[30]	Fisetin	In Vitro: Murine pre-osteoblasts (MC3T3-E1) and chondrocytes (ATDC5). Model: Replicative senescence. Dose: 25 and 50 µM × 48h. In Vivo: Zmpste24$^{-/-}$ progeroid model mice. Dose: 100 mg/kg oral gavage, weekly × 4 wk, treatment at 3 months	In Vitro: SA-β-gal+ cells ↓. In Vivo: p16 mRNA (kidney) ↓; SASP ↓ (IL-6, TGF-β mRNA in kidney); Trabecular bone density loss ↓ (attenuated)	Anti-apoptotic pathway (SIRT1, BCL-2/BCL-XL, HIF-1α); SnC clearance; Senolytic (prevents bone degeneration in progeria)	Senolytic

Author & Year	Compound/Extract	Study Type & Details	Key Senescence Biomarkers (Treatment Effect)	Mechanism & Pathway	Senotherapeutic Classification
[31]	Fisetin	In Vivo: Telomerase-deficient progeria mice G3 (Tert -/-). Senescence inducer: Genetic telomere dysfunction (premature aging). Dose: Continuous dietary supplementation 500 mg/kg of fisetin in standard chow and intermittent gastric gavage 20 mg/kg × 3 times per wk for 7 wk.	$p16^{INK4a}$ ↓; $p21^{CIP1}$ (kidney and liver); Collagen fiber deposition ↓ (Fibrosis ↓); ALT ↓; Urea ↓ (kidney/liver function markers); ↓ Stanniocalcin-1 (Stc-1) mRNA ↓	AKT signalling pathway inhibition (via Stc1 suppression → apoptosis of SnCs); Senolytic (reverses premature aging signs)	Senolytic
[32]	Fisetin	In Vitro: HUVECs, Human Aortic Endothelial Cells (HAECs). Senescence inducer: Replicative senescence (passage 15) × 48h. In Vivo: Aged wild-type C57BL/6N mice (27 months) & p16-3MR transgenic mice. Dose: 100 mg/kg/day oral gavage, intermittent (1 wk on, 2 wks off, 1 wk on)	SA-β-gal ↓ (in vitro); p16 ↓ (vascular); Arterial stiffness (PWV) ↓; Collagen deposition ↑; Endothelium-dependent dilation (EDD) ↑; Mitochondrial ROS ↓; SASP-related inflammation ↓	$p16^+$ SnC clearance; Mitochondrial ROS/oxidative stress reduction; Endothelial function improvement; Senolytic	Senolytic
[33]	Fisetin	In Vitro: Mouse aortic endothelial cells (ECs), HUVECs, and mouse VSMCs. Senescence induced by: High glucose (HG, 35 mM × 7 days). Dose: 20-30 μM. In Vivo: Type 2 diabetes mellitus (T2DM) C57BL/6 mice. Senescence inducer: HFD + low-dose streptozotocin 50 mg/kg × 5 days, and aged mice (16-mo). Optimal dose: 100 mg/kg/day, oral gavage, 3 consecutive days per 2 wks × 12 wks (intermittent).	SA-β-gal ↓ (aorta and cells); $p16^{INK4a}$ ↓; $p21^{CIP1}$ ↓; γ-H2AX ↓; Cell viability (SnC) ↓; SASP ↓ (in circulation & aorta) (IL-6 ↓, IL-8 ↓, IL-18 ↓, IL-1α ↓, IL-1β ↓, MMP-2 ↓, MMP-9 ↓)	PI3K/AKT pathway inhibition; Bcl-2/Bcl-xL ↓ (anti-apoptotic); Bax ↑ / Bak ↑ (pro-apoptotic); SASP suppression; Aortic SnC clearance	Senolytic
[34]	Fisetin	In Vivo: Female C57BL/6 mice (8-12 mo). Senescence inducer: DSS-induced colitis. Dose: 100 mg/kg oral gavage.	p16 ↓;p21 ↓; p53 ↓; β-gal ↓; SASP ↓ (CXCL1; MCP1); Apoptosis ↑; gut permeability maintained; A*kkermansia*, *Ruminococcu*s (beneficial) ↑; miR-149-5p ↑; miR-34a-5p ↑; Restoration of colon health; Promotes mucus production, immune response, and produces short-chain fatty acids	NF-κB pathway inhibition; Anti-inflammatory; Gut microbiota modulation (Akkermansia muciniphila ↑); Gut barrier integrity ↑; Senolytic and senomorphic	Dual (Senolytic + Senomorphic)
[35]	Fisetin	In Vitro: HUVECs Inducer: Replicative senescence. Dose: 5–15 μM (dose-dependent killing). In Vivo: Progeroid mice ($Ercc1^{-}/\Delta$). Dose: 100 mg/kg (Oral), Once weekly for 4 wks.	In Vitro: Selective clearance of senescent HUVECs. In Vivo: p16 ↓, p21 ↓ in liver, lung, spleen, kidney. Pathology ↓ in progeroid mice.	Anti-apoptotic pathway targeting; SnC selective elimination	Senolytic

Author & Year	Compound/Extract	Study Type & Details	Key Senescence Biomarkers (Treatment Effect)	Mechanism & Pathway	Senotherapeutic Classification
[36]	Fisetin	In vivo: Rats destabilization of medial meniscus (DMM) surgery-induced osteoarthritis model. In vitro: Chondrocytes-induced IL-1β to mimicking OA cell model.	SA-β-gal ↓; SIRT6 ↓; ROS ↓; Apoptosis ↓ (chondrocytes)	SIRT6 pathway modulation; ROS reduction; Apoptosis reduction in chondrocytes	Dual (Senolytic + Senomorphic)
[4]	Fisetin	In Vivo: Wild-type C57BL/6 mice (Old: 22-24 months) & Progeroid ($Ercc1^{-}/\Delta$). Dose: 100 mg/kg, Oral, Intermittent (5 days on, 2 days off, for 2 weeks or life-long)	$p16^{Ink4a}$ ↓; $p21^{Cip1}$ (Fat, Spleen, Liver, Kidney); SASP ↓ (IL-6, TNF-α, MCP-1) in serum and tissues; Median and maximum lifespan ↑ (~10% extension);	Reduction of oxidative stress; restoration of tissue homeostasis. Senolytic (Extends healthspan and lifespan; reduces frailty).	Dual (Senolytic + Senomorphic)
[37]	Fisetin	In Vivo: Lupus nephritis (MRL/lpr) mice. Dose: 50 mg/kg, intraperitoneal, twice weekly × 4 wks (12 to 16 weeks of age)	SA-β-gal ↓ (kidney tubular epithelial cells); $p16^{Ink4a}$ and p21 mRNA ↓ (Kidney); Renal fibrosis ↓ (Collagen I); ↓ Glomerulosclerosis ↓; interstitial inflammation	Selective elimination of senescent tubular epithelial cells; Inhibition of proliferative fibroblasts via potential cell-cycle arrest.	Senolytic
[38]	Fisetin	In Vivo: $Mdr2^{-/-}$ mice (primary sclerosing cholangitis model). Genetic Comparison: INK-ATTAC and $p16^{-/-}$ × $Mdr2^{-/-}$ mice. Dose: NA	p16 ↓ and p21 ↓ (80–90% reduction); Inflammatory markers (TNF-α, IL-1β, MCP-1) ↓; Hepatic fibrosis ↓ (~60% ↓); Cholangiocyte senescence ↓	Selective killing of senescent cholangiocytes; Profibrotic SASP reduction	Senolytic
[39]	D+Q	In Vitro: HDFs. Senescence inducer: Replicative & Ionizing Radiation (IR) 10 Gy X-rays. In Vivo: Mouse/human chimeric model (aged human skin grafts on nude mice). Dose: 50 mg/kg, intraperitoneal injection × 30 days.	In Vitro: SA-β-gal ↓; Selective elimination of senescent HDFs. In Vivo: SA-β-gal ↓, SASP ↓ (MMP-3,-9, IL-1, -6 and TNF)-α,; Collagen density ↑	Caspase-3/8/9 activation (intrinsic and extrinsic apoptosis pathways); SnC elimination → skin rejuvenation	Senolytic
[40]	Fisetin & D+Q	In Vivo: Aged mice C57BL/6 mice (20-24 mo) infected with SARS-CoV-2. Dose: Fisetin 20 mg/kg/day + D+Q: dasatinib 5 mg/kg + quercetin 50 mg/kg, intermittent dosing.	p16 ↓; p21 ↓; SASP ↓ (IL-1α, IL-1β, IL-6, IL-10, TNFα, MCP-1, IP-10, CXCL1, CXCL2, PAI-1, PAI-2)	TGF-β pathway, IL-1α-mediated paracrine effects, Immune cell dysfunction, PAMP-induced SASP amplification & Viral entry protein regulation (ACE2, TMPRSS2)	Dual (Senolytic + Senomorphic)
[41]	Fisetin & D+Q	In Vitro: HKEs, human umbilical vein endothelial cells (HUVECs), and HBAs. Senescence inducer: X-ray radiation 20 Gy. In Vivo: Naturally aged mice (26-29 mo, male and female), Diet-induced obese mice, and INK-ATTAC transgenic mice for genetic clearance comparison. Dose: fisetin 100	SA-β-gal ↓; p16 ↓; IL-1α ↓ (brain/ hippocampus, urine); IL-1β ↓, IL-18 ↓, CXCL1 ↓, IL-6 ↓, TNF-α ↓, PAI-1 ↓ (in vitro); SnCs viability/apoptosis; Urinary, kidney, and brain α-Klotho	SCAPs disruption; pro-survival network inhibition; p53/p21 pathway modulation; mTOR pathway inhibition; TGF-β pathway modulation; Apoptosis induction	Senolytic

Author & Year	Compound/Extract	Study Type & Details	Key Senescence Biomarkers (Treatment Effect)	Mechanism & Pathway	Senotherapeutic Classification
		mg/kg; D+Q: dasatinib 5 mg/kg + quercetin 50 mg/kg.			
[42]	Fisetin & D+Q	In Vivo: Male C57BL/6J mice 6 to 9 mo. Dose: Fisetin 50 mg/kg and D 5 mg/kg + Q 50 mg/kg, oral weekly.	SA-β-gal ↓, SASP ↓ (IL-6 and TNF-α)	SCAPs, disrupt pro-survival networks inhibition	Dual (Senolytic + Senomorphic)
[43]	Fisetin & D+Q	In Vivo: C57BL/6 mice. Dose: Fisetin: 50 mg/kg and D 5 mg/kg + Q 50 mg/kg, oral weekly.	$p16^{INK4a}$ ↓; $p21^{Cip1}$↓; SASP ↓ (TNFα; MCP-1; IL-6; IL-10; MCP-1)	PI3K/AKT pathway; mTOR pathway; Apoptosis induction; Sex-dimorphic mechanisms: males show greater senescence burden and fisetin response than females	Senolytic
[44]	Fisetin & Resveratrol	In Vitro: Chondrogenic progenitor cells (CPCs) isolated from osteoarthritic knee cartilage. Dose: Fisetin: 5μM-100μM; Resveratrol: 5μM-100μM. Complementary approach: In silico molecular docking analysis.	SA-β-gal ↓; p53 ↓; $p38^{MAPK}$ ↓; $p16^{INK4A}$ ↓; $p21^{Waf1/Cip1}$ ↓; SASP ↓ (MMP-9 and MMP-13 (gene and protein), IL-1β (gene/protein), TGF-β (gene) IL-6 (gene)); cell viability and apoptosis ↑	p53/p21 pathway suppression; NF-κB pathway	Senomorphic
[45]	Q+Q + Fisetin + Epigallocatechin-3-gallate	In Vitro: Senescent murine and human fibroblasts. In Vivo: Mouse and rat models. Flavonoid-copper/iron interaction study.	SA-β-gal ↓; p16 ↓; p21 ↓; SASP ↓; Apoptosis ↑; ROS ↑; DNA damage markers ↓	ROS generation via flavonoid-metal ion interaction → selective senolytic activity (quercetin/fisetin oxidation in presence of Cu/Fe); SASP reduction (senomorphic); Apoptosis induction	Dual (Senolytic and Senomorphic)
[46]	Sulforaphane + D+Q, & combination	In vivo: Female Rattus norvegicus, 14-15 months; HFD-induced obesity for 8 months; Dose: D 5 mg/kg + Q 50 mg/kg and D+Q & Sulforaphane 5 mg/kg, subcutaneously	SA-β-gal ↓; p21 ↓; IL-6 ↓; ROS ↓; MCP-1 ↓; Apoptosis/Cell Viability: BCL-2 protein ↓; Synaptophysin ↑; PSD95 ↑	SnC anti-apoptotic pathways (SCAP): Influencing the BCL-2/Bax balance modulation; NF-κB pathway inhibition; Gut-brain axis modulation (gut dysbiosis ↓ → neuroinflammation ↓)	Dual (Senolyitc + Senomorphic)
[47]	Resveratrol	In Vitro: Primary HDFs. Senescence inducer: Drug-induced premature senescence) Concentration: 10, 25, 50 μM (48h treatment)	SA-β-gal ↑ (Dose-dependent); ↑ p53 ↑; p21 ↑; DNA damage markers ↑ (gamma-H2AX foci); SIRT1 and SIRT2 protein ↓	ATM-p53-p21 pathway activation and downregulation of SIRT1/SIRT2. Pro-senescent (Note: at these concentrations ≥25 μM in normal fibroblasts, resveratrol INDUCES rather than prevents senescence)	Pro-senescent (dose-dependent; contra-indicated in normal fibroblasts at ≥25 μM)
[48]	Resveratrol	In Vitro: Human ovarian granulosa-like tumor cells. Senescence inducer: Cisplatin	SA-β-gal ↓; $p16^{INK4a}$ ↓; $p21^{CIP1}$ ↓; Apoptosis rates ↓	Upregulation of SIRT1 and p53/p21 pathway suppression	Senomorphic

Author & Year	Compound/Extract	Study Type & Details	Key Senescence Biomarkers (Treatment Effect)	Mechanism & Pathway	Senotherapeutic Classification
		(Chemotherapy-induced premature senescence). Dose: 20 μM			
[49]	Resveratrol	In vitro: Human airway epithelial cells (BEAS-2B). Senescence inducer: Cigarette smoke extract (CSE) exposure. Dose: 10μM, 25μM, and 50μM. Mechanism validation: miR-34a-5p mimic transfection, dual-luciferase reporter assay	SA-β-gal ↓; p16 ↓; p21 ↓; p53 ↓; IL-1β ↓; IL-6 ↓; IL-8 ↓; TNF-α ↓; NF-κB p65 ↓; Proliferation/ viability; miR-34a-5p; SIRT1 ↑	SIRT1 pathway (direct target); NF-κB pathway (deacetylation). Reduction of miR-34a-5p → ↑ SIRT1 → ↓ NF-κB → ↓ SASP → ↓ Senescence	Senomorphic
[50]	Resveratrol	In Vitro: Human Bone Marrow Stromal Cells (hBMSCs). Model: replicative senescence & Adipogenic differentiation. Dose: 10 μM (optimal anti-aging dose)	SA-β-gal ↓; $p16^{INK4a}$ ↓; $p21^{CIP1}$ ↓; Intracellular ROS ↓; Cell proliferation (Ki67) ↑; Stemness markers ↑	ROS scavenging (antioxidant). Adipogenic differentiation inhibition. Stem cell function preservation. Delays replicative senescence	Senomorphic
[51]	Polydatin	In vitro: NPCs. Senescence inducer: TNF-α (50 ng/mL). Dose: 200 μM-400 μM. In vivo: rats; puncture-induced IVD degeneration; Dose: 50 mg/kg oral, daily.	SA-β-gal ↓; p53 ↓; p16 ↓; MMP-3 ↓; MMP-13 ↓; Aggrecan ↑; Collagen II ↑; ROS ↓;	Nrf2/Keap1 pathway activation. Mitochondrial protection. Antioxidant	Senomorphic
[52]	Luteolin	In Vitro: Auditory cell line (HEI-OC1). Senescence inducer: H_2O_2. Dose luteolin: 10, and 20 μM.	SA-β-gal ↓; ROS ↓; Acetylated p53 ↓; SIRT1 ↑	SIRT1-mediated p53 deacetylation. Protective against oxidative stress	Senomorphic
[53]	Luteolin	In Vitro: HDFs. Senescence inducer: UVA radiation (photoaging). Dose: 2.5, 5, 10 μM.	SA-β-gal ↓; ROS ↓; MDA ↓; MMP-9 ↓; MMP-3 ↓	Nrf2/HO-1 pathway activation, MAPK/AP-1 pathway inhibition to prevent collagen degradation	Senomorphic
[54]	Luteolin	In vitro: Canine and feline vascular endothelial cells (cVECs and fVECs). Senescence inducer: Dox, dose at 0.8 μM (cVECs) and 1.6 μM (fVECs) for 24 h. Dose Luteolin :0.625–2.5 μM co-treatment. In vivo: Male senescence-accelerated mouse prone 8 (SAMP8). Dose Luteolin: 10 mg/kg/day orally for 8 wks ; multi-omics analysis	SA-β-gal ↓ ~10% (0.625 μM); SASP (IL-1β, TNF-α, IL-6, IL-8, MMP-1, MMP-3, MCP-1) ↓; MDA ↓; SOD ↓; VEGF and bFGF restored; improved cardiac, arterial wall, and hepatic histology	Aldehyde dehydrogenase 1 (ALDH1A1/A2/A3), NF-κB and immunosenescence pathways, Metabolomic validation: retinoic acid fold change, 9-cis-retinal; pro-lipolytic metabolites elevated (PGE2, EPA); lipotoxic metabolites reduced (arachidonic acid, S1P, 9(S)-HODE)	Senomorphic
[55]	Curcumin (encapsulated in neutrophil-derived nanovesicles, Cur-NVs)	In Vitro: HT22 hippocampal neurons Inducer: Aβ oligomers. Dose: NA. In Vivo: APP/PS1 transgenic Alzheimer's mice model. Dose: 5 mg/kg intravenous.	p16 and p21 ↓ in brain; Aβ plaque deposition ↓; SASP ↓ (TNF-α, IL-1β, IL-6);	3R Strategy (Remove, Remodel, Repair) of curcumin. Blood-brain barrier penetration via nanovesicles; Immune microenvironment modulation (anti-inflammatory). Neuronal repair	Dual (Senolytic + Senomorphic)

Author & Year	Compound/Extract	Study Type & Details	Key Senescence Biomarkers (Treatment Effect)	Mechanism & Pathway	Senotherapeutic Classification
[56]	Curcumin	In Vitro: Primary neonatal Sprague-Dawley rats cardiomyocytes. Senescence induction: D-gal 10 g/L × 48 h). Dose curcumin: 1 µM, 5 µM, 10 µM × 48 h.	SA-β-gal ↓; p53 ↓; p16 ↓; Intracellular ROS ↓; LC3-II/LC3-I ratio ↑; p62 ↓	SIRT1 activation, AMPK phosphorylation, Oxidative stress reduction; Autophagy induction (mTOR dependent). Dose-dependent effects observed across all parameters	Senomorphic
[57]	Curcumin & hesperetin (combination)	In Vitro: Human neuroblastoma cell SH-SY5Y cells. Senescence inducer: D-galactose. Dose: Curcumin 1µM, hesperetin 1µM, combination: Curcumin 1µM + hesperetin 1µM. In Vivo Wistar rats. Senescence induction: D-gal 250 mg/kg subcutaneous daily. Dose : Curcumin 50 mg/kg, hesperetin 50 mg/kg, and combination (curcumin 50 mg/kg + hesperetin 50 mg/kg), Oral × 3 mo.	In Vivo & In Vivo: SA-β-gal ↓; p16 ↓; p21 ↓; Apoptosis ↑; Antioxidant enzymes (in vitro): SOD1 ↑ glutathione peroxidase 1 (Gpx1) ↑	Apoptosis induction and antioxidant.	Dual (Senolytic + Senomorphic)
[58]	Rutaecarpine (from *Evodia rutaecarpa*)	In Vitro: Lung epithelial cells. In Vivo: Pulmonary Fibrosis induced mice model). Induction: Bleomycin.	SA-β-gal ↓; p16 ↓; p21 ↓; Epithelial-Mesenchymal Transition (EMT) markers ↓; Fibrosis scores ↓	TGF-β1 signaling inhibition, EMT inhibition, restoration of E-cadherin promoter activity, anti-fibrotic and anti-senescence.	Senomorphic
[59]	Apigenin and Rutaecarpine	In Vitro: hBMSCs (hTERT immortalized cell line and primary from young donors age 25–26 and elderly osteoporotic patients age 86–91). Senescence inducer: Tert-butyl hydroperoxide (TBHP) at 50-55 µM. Ex Vivo: Embryonic chick femur organotypic culture. Dose: 1 µM for both	SA-β-gal ↓; p16 ↓; p21 ↓; p53 ↓; SASP ↓ (IL-1β, IL-6, IL-8, TNF-α, MMP3,); ROS production ↓; HMOX1 ↑; SOD2 ↑; SOD3 ↑	TGF-β/SMAD2 pathway activation, oxidative stress pathway, senomorphic activity, antioxidant defence, NF-κB pathway inhibition, Synergistic combination effect	Senomorphic
[60]	Apigenin	In Vitro Primary human fibroblasts. Senescence inducer: IR-induced, oncogenic RAS, replicative. Dose Apigenin: 10µM (optimal dose).	SASP ↓ in 3 human fibroblast strains (IL-6 ↓, IL-8 ↓, CXCL1 ↓, MMP-3 ↓, GM-CSF ↓); SA-β-Gal ↔; p16 ↔; p21 ↔	NF-κB pathway inhibition	Senomorphic
[61]	Apigenin	In Vitro: Primary human prostate stromal cell line. Senescence inducer: Chemotherapy and Bleomycin. In Vivo: Mice. Senescence inducer: Chemotherapy-induced fatigue/resistance and naturally aged model.	SASP ↓ (IL-6, IL-8, CXCL1) Chemoresistance ↓; Physical dysfunction in aged mice	Disruption of ATM/p38MAPK–HSPA8 interaction required for SASP; Senomorphic	Senomorphic
[62]	Apigenin	In Vivo: WI-38 human embryonic lung fibroblast cells. Senescence inducer: H_2O_2-SIPS, 150 µM for 2 h and doxorubicin 100 ng/mL. Dose apigenin: 1, 5, and 10 µM.	SA-β-gal ↓; p16^{INK4a} ↓; p21^{CIP1} ↓; phospho-Rb ↑; SIRT1 ↑; nicotinamide adenine dinucleotide (NAD+) ↑; cluster of	SIRT1-NAD+-CD38 axis.	Senomorphic

Author & Year	Compound/Extract	Study Type & Details	Key Senescence Biomarkers (Treatment Effect)	Mechanism & Pathway	Senotherapeutic Classification
			differentiation 38 (CD38) activity ↓ (concentration-dependent manner)		
[63]	Ginsenoside Rg1	In Vitro: Mouse BMSCs co-culture with hematopoietic stem/progenitor cells (HSPCs). Senescence inducer: D-galactose 166 mM × 48h. Dose: 50 μM. In Vivo: male and female C57BL/6J mice, males and females. Senescence inducer: D-galactose 120 mg/kg/day × 42 days. Dose: 40 mg/kg/day.	SA-β-gal ↓; p53 ↓; p21 ↓; ROS ↓ (MDA ↓, catalase ↑); SASP ↓ (IL-1β, IL-6, TNF-α) – before and after co-culture	NF-κB pathway inhibition NF-κB pathway inhibition, TNF-α signaling downregulation; Antioxidant pathways activation, senomorphic and senolytic	Dual (Senolytic + Senomorphic)
[64]	Red Ginseng extract	In vivo: Male C57BL/6J mice. Young: 9 wks vs Aged: 19 mo. Dose: 300 mg/kg oral gavage every 4 days.	SA-β-gal ↓; p53/p21 ↓; p16/p15 ↓; Improved metabolic function weeks; Apoptosis ↓	NF-κB pathway; p53/p21 pathway; TGF-β pathway; Apoptosis induction	Senomorphic
[65]	Black Ginseng extract	In vivo: Male C57BL/6J mice, young (9 wk) vs. aged (19 mo). Dose: 300 mg/kg/bw oral gavage every 4 days.	SA-β-Gal ↓; p53 ↓; p21 ↓; p16 ↓; p15 ↓; Metabolic function ↑	p53/p21 pathway modulation; Senomorphic	Senomorphic
[66]	Epigallocatechin Gallate (EGCG)	In Vitro/Ex Vivo: Peritoneal macrophages from C57BL/6J mice (young: 4 mo vs 18 mo); 3T3-L1 preadipocytes. Senescence inducer: H_2O_2 150 μM × 3h/day × 3 consecutive days. Dose: 10 μM.	p53 ↓; $p21^{WAF1}$ ↓; $p16^{Ink4a}$ ↓; SASP ↓ (TNF-α, IL-6, IL-1β, IL-10); ROS ↓	ROS production reduction, inflammatory cytokine dysregulation reversal, and anti-inflammaging	Dual (Senolytic + Senomorphic)
[67]	EGCG	In vitro: 3T3-L1 murine preadipocytes. Induction: H_2O_2-induced premature senescence (150 μM × 3h × 3days). Dose: 50 μM 100 μM.	SA-β-gal ↓ (>60% ↓); ↓ Cell size ↑; SASP ↓ (TNF-α, IL-6, iNOS, NF-κB); p53 ↓; $p21^{WAF1}$ ↓; $p16^{INK4a}$ ↓; Proliferative capacity ↑; Moderate SnC viability; Dose-dependent SnC apoptosis	PI3K/Akt/mTOR pathway inhibition, Oxidative stress reduction, Apoptotic pathway regulation (moderate senolytic mechanism), senomorphic	Dual (Senolytic + Senomorphic)
[68]	EGCG and EGCG-resveratrol-spermidine mix	In Vitro: 3T3-L1 mouse preadipocytes. Senescence inducer: Bromodeoxyuridine (BrdU) 100 μM. Dose: EGCG 50 μM and 100 μM; Mix (40% EGCG + 40% resveratrol + 20% spermidine) 20 and 30 μM.	SA-β-gal ↓; p21 ↓; SASP ↓ (IL-6), EGCG strongest; Nrf2 and SIRT3 ↑	SIRT3 activation (mitochondrial); Nrf2/ARE pathway activation; Senomorphic (primarily) with potential senolytic activity	Dual (Senolytic + Senomorphic)
[69]	Procyanidin C1	In Vivo: C57BL/6 male mice. Model: Unilateral ureteral obstruction-induced renal fibrosis. Dose: 20 mg/kg intraperitoneal twice weekly. In Vitro: Human proximal tubular epithelial cells (HK2 cells). Senecence induction: H_2O_2 800 μM × 48h	SA-β-gal ↓; p21 ↓; p16 ↓; γH2AX ↓; Apoptosis induction: TUNEL-positive cells ↑ (~3-fold ↑ in SnC); Moderate ↓ SnC; SASP ↓ (TNF-α, IL-6, iNOS, IL-1β, MMP1,	ANGPTL4/NOX4 signaling inhibition, apoptotic pathway activation (senolytic at ≥50 μM); NF-κB pathway	Dual (Senolytic + Senomorphic)

Author & Year	Compound/Extract	Study Type & Details	Key Senescence Biomarkers (Treatment Effect)	Mechanism & Pathway	Senotherapeutic Classification
		and γ-radiation 10-Gy. Co-culture: Human kidney fibroblasts with SnC conditioned medium.	MMP3, TIMP1, TIMP2); NF-κB ↓; Fibroblast activation ↓		
[70]	Procyanidin C1	In Vitro: Granulocytes and myeloid cells. In Vivo: Aged Mice (20 mo). Dose: 20 mg/kg intermittent	SA-β-gal ↓ (bone marrow & Spleen); ↓ SASP (B cells); ↑ Grip strength (Physiological improvement)	Senolysis (apoptosis via ROS/mitochondrial dysfunction in granulocytes/myeloid cells)	Dual (Senolytic + Senomorphic)
[71]	Procyanidin C1	In Vitro: Human stromal cells (PSC27), fibroblasts (WI-38), HUVECs. Senescence inducer: Replicative, Oncogene-induced (RAS), Radiation 10 Gy. Dose: ≥ and <50 μM). In Vivo: Aged mice (24-27 mo) & Chemo-treated mice. Dose: 20 mg/kg intermittent	SA-β-gal ↓; p16INK4a ↓; p21CIP1 ↓; SASP ↓ (IL-6, IL-8, MMPs); Median post-treatment lifespan ↑ (by ~64%); Dose-dependent activity	ROS-dependent apoptosis (senolytic at ≥50 μM); NF-κB pathway inhibition (senomorphic at <50 μM); Dual concentration-dependent activity	Dual (Senolytic + Senomorphic)
[72]	Berberine	In Vitro: *S. cerevisiae* (replicative lifespan, 20 μg/mL); human fetal lung diploid fibroblasts (2BS, WI-38); Senescence inducer: Replicative senescence, Dox 0.1 μM, H_2O_2 0.8 mM × 48h. In Vivo: Male C57BL/6J mice. Senescence inducer: Doxorubicin and naturally aged mice (18-22 mo). Dose: 50 mg/kg oral daily × 1–4 mo.	SA-β-Gal ↓; p16 ↓; Replicative lifespan ↑ (yeast: 28% extension; human fibroblasts: extended PD); Cell morphology improvement ↑; G1→S progression ↑; Cells in S/G2-M phase ↑ (~18% increase)	p16/Cyclin D1/CDK4/RB axis modulation; mTOR pathway inhibition; Cell cycle checkpoint regulation	Senomorphic
[73]	Myricetin	In Vitro: Human nucleus pulposus cells (NPCs) - isolated from mild IDD patients (Pfirrmann grade I-II). Senescence inducer: H_2O_2 100 μM × 24h (primary). Dose: 10, 50, 100, 500 μM.	SA-β-gal ↓; p16 ↓; p21 ↓; IL-6 ↓; SASP ↓	SERPINE1 regulation and anti-inflammatory	Senomorphic
[74]	Anthocyanins	In Vitro: Mouse AML12 cells (normal hepatocyte cell line). Dose: 50 or 100 μM. In Vivo: Male C57BL/6J mice (6-8 wks). Senescence inducer: D-gal 800 mg/kg, subcutaneous daily × 8 weeks. Dose: 20 mg/kg and 40 mg/kg.	SA-β-gal ↓; p16 and p21 protein ↓; Improved cell morphology; SASP ↓ (IL-1β mRNA, ↓ IL-6 mRNA, TNF-α mRNA), Protected liver function: ↓ ALT levels ↓; AST levels ↓; DNA damage: γ-H2AX ↓(both doses)	NF-κB pathway, apoptosis induction, anti-inflammatory, and liver function protection	Senomorphic
[75]	Cyanidin-3-glucoside (C3G)-rich anthocyanin extracts of *Sambucus*	In vitro: HEK293T cells. Senescence inducer: D-gal (75 g/L, 72h). Doses of intervention: 100–400 μg/mL.In vivo: Kunming mice. Senescence inducer: D-gal i.p. 500 mg/kg/day × 6 wks. Dose of intervention: 80 mg/kg/day oral (in vivo)	SA-β-gal ↓ (in vitro + in vivo); p16INK4a ↓; p21CIP1/WAF1 ↓; p53 ↓; ROS ↓; SOD ↑; CAT ↑; MDA ↓; Body weight ↑; liver and spleen index ↑ (in vivo). Effects	PI3K/AKT/mTOR pathway inhibition → p53/p21 axis suppression + p16/Rb pathway suppression → SnC burden ↓ antioxidant enzyme upregulation	Dual (Senolytic + Senomorphic)

Author & Year	Compound/Extract	Study Type & Details	Key Senescence Biomarkers (Treatment Effect)	Mechanism & Pathway	Senotherapeutic Classification
	canadensis (elderberry)	C3G identified as principal bioactive by ^{1}H NMR metabolomics.	dose-dependent (100–400 μg/mL in vitro).	(SOD, CAT). C3G identified as key active compound by ^{1}H NMR.	
[76]	Anthocyanins	In Vitro: HUVECs. Senescence inducer: Replicative. In Vivo: Male fischer 344 rat, young (6 mo) vs. Old (22 mo), Dose: 10 mg/kg/day oral gavage × 8 weeks.	SA-β-gal positive cells ↓; ROS ↓ (Superoxide anion) levels; NO ↑; Inflammatory markers (VCAM-1 ↓, ICAM-1 ↓); Endothelial function ↑	Endothelial function and vascular senescence reduction	Senomorphic
[77]	Delphinidin (anthocyanins)	In vitro: BV2 microglia with Aβ42-induced senescence; Dose: 30 μM. In Vivo: APP/PS1 mice and naturally aged mice (18 mo). Dose; 15 mg/kg in vivo intravenously.	SA-β-gal ↓; p16 ↓; p21 ↓; IL-1β ↓; IL-6 ↓; TNF-α ↓; ROS ↓	AMPK/SIRT1 pathway activation (via direct SIRT1 binding), and senomorphic (microglial senescence prevention)	Senomorphic
[78]	Parishin (phenolic glucoside from *Gastrodia elata*)	In Vitro: Cell line: Human coronary artery endothelial cells (HCAECs). Senescence model: Replicative senescence. In Vivo: Male C57BL/6 mice, 19 mo (naturally aged)	γ-H2AX ↓; p16Ink4a ↓; SASP ↓ (IL-6, CXCL9); Lamin B1 ↑; GDF15 (growth differentiation factor 15) ↓	Klotho/FoxO1 and SIRT signalling pathway activation and cardioprotective and vascular anti-aging	Senomorphic
[79]	Parishin	In Vivo. Naturally aged C57BL/6 mice (19 mo) compared to young mice (12 weeks), Dose parishin: 20 mg/kg/day × 8 weeks.	p16Ink4a ↓; p21Cip1 ↓; IL-6 ↓; γH2AX ↓; PARP1 ↓; SIRT1 ↑; Improved cardiac function, cardiac weight/bw ratio, and no hypertrophy and fibrosis; Gja1↓; Cyp2e1, Ccna2, Cdca3, and Fgf12 ↓ in heart tissues	SIRT1 pathway activation and Gja1 downregulation (transcriptomic analysis)	Senomorphic
[80]	*Centella asiatica* (50% ethanol extract, Active compounds: Asiatic acid, Madecassic acid, Asiaticoside, and Madecassoside)	In vitro Human epidermal keratinocytes (HaCaT cells). Senescence inducer: H_2O_2. Dose extract: 50 μg/mL, 100 μg/mL, and 200 μg/mL	SA-β-gal ↓; p16 ↓; p21 ↓; ROS ↓ Apoptosis ↑	Apoptosis induction; ROS reduction (Antioxidant); Senomorphism	Dual (Senolytic + Senomorphic)
[81]	*Silybum marianum* flower extract (Main compound: Apigenin)	In Vitro: human hair follicle dermal papilla cells (HFDPCs). Senescence model: Replicative senescence. Dose extract: 10, 50 and 100 μg/mL × 72h. Positive controls: Finasteride (20 μg/mL), EGCG (20 μM)	SA-β-gal positive cells ; ROS ↑; SASP ↓ (IL-6); morphological changes	TGF-β pathway; Senomorphic activity	Senomorphic
[82]	*Silybum marianum* flower extract	In Vitro Human neonatal dermal fibroblasts (HDFs) and human epidermal keratinocytes	SA-β-gal positive cells ↓; p53 ↓; p16INK4A ↓; p21CIP1 ↓; ROS ↑;	Apoptosis induction; and Senomorphic activity; Antioxidant activity	Dual (Senolytic and Senomorphic)

Author & Year	Compound/Extract	Study Type & Details	Key Senescence Biomarkers (Treatment Effect)	Mechanism & Pathway	Senotherapeutic Classification
		(HEKa). Senescence model: Replicative senescence, and induction by H_2O_2 200 µM, doxorubicin 100 nM and etoposide 10 µM × 3 days. Dose extract: 50, 100, and 200 µg/mL.	SASP ↓ (IL-6 & MMP-1); morphological changes; annexin V-FITC-positive apoptotic cells ↑		
[83]	*Physalis alkekengi L.* (active compounds saponins, rutin, and luteolin	In Vivo: Male ICR mice. Senescence inducer: D-galactose 150 mg/kg via subcutaneous injection . Dose extract: 3, 10, and 30 mg/kg. Positive control: Vit E 100 mg/kg. Identification of active compound by HPLC	SA-β-gal ↓ (liver, spleen, and hippocampus); Total antioxidant capacity (T-AOC) ↑; T-SOD ↑; MDA ↓; CAT ↑, and Acetylcholinesterase (AChE) activity ↓ (brain only)	Antioxidant Defense, Improved learning and memory, enhanced immune function and anti-fatigue and senomorphic	Senomorphic
[84]	*Physalis alkekengi L.* ethyl acetate layer (contain standardized: Luteolin-7,4′-di-O-β-D-glucopyranoside, Physalins and flavonoids)	In Vitro: lHFF-1 fibroblasts, murine macrophages (RAW264.7), HaCaT and reconstituted human epidermis (RHE). Senescence inducer: D-galactose 20 mg/mL (IHFF-1), H_2O_2 400 µM (HFF-1) and 800 µM (HaCaT) and Lipopolysaccharides (LPS) in RAW264.7 macrophages (1 µg/mL), HaCaT cells and the RHE (10 µg/mL). Dose extract: HFF-1 cells (100 ng/mL), RAW264.7 cells (10-200 µg/mL), HaCaT (500 ng/mL), RHE 3D skin model (1 mg/mL). In Vivo: Zebrafish embryos. Dose: 30-100µg/mL	HFF-1: SA-β-gal ↓; IL-1β ↓; ROS ↓; MMP-9 ↓. RAW264.7: NO ↓; NOS ↓; dose-dependent protective effect against LPS-induced cell apoptosis; IL6 ↓; IL-1β ↓; MMP-9 ↓; IL-10 ↓; IL-α ↓; TNF-α ↓; COX-2 ↓; PGE2 ↓. HaCaT: LPS-Induced Cytotoxicity ↓; TNF-α ↓; IL-6 ↓; IL-1β ↓. RHE 3D: IL-α ↓. In Vivo: ROS ↓; IL6 ↓; Neutrophil count ↓	Senolytic; NF-κB pathway; Anti-inflammatory pathway; Antioxidant; suppress SASP	Dual (Senolytics + Senomorphic
[85]	Composite botanical extract- *Matricaria chamomilla* extract aq., + *Solidago virgaurea* 40% EtOH + green tea extract aq. + *Ganoderma lucidum* 40% EtOH	In Vitro: HDFs. Senescence inducer: Etoposide 25 µM. Dose extract: Green tea: 70.4 µg/mL (1:54 dilution of original extract), chamomile: 211.1 µg/mL (1:18 dilution), goldenrod: 104.2 µg/mL (1:18 dilution). Positive control: Quercetin 25 µM	All extracts: SA-β-gal ↓; p16INK4a ↔; p21 ↔. Chamomile: IL-6 ↑; IL-8 ↑; IL-1β ↑; IL-1α ↑; CXCL-1 ↑; MMP3 ↑; HLA-E ↑. Goldenrod: IL-6 ↓. Green tea: IL-6 ↔	Apoptosis induction; Senolytic activity (all tested extracts); Senomorphic activity (goldenrod and green tea); Antioxidant potential; chamomile's pro-inflammatory effect	Senolytic (all tested herbal extracts) and Senomorphic activity (goldenrod and green tea)
[86]	Cistus essential oils (*C. laurifolius, C. monspeliensis*)	In Vitro: HaCaT keratinocytes. Senescence inducer: Ultraviolet B (UVB) 10 mJ/cm². Dose oils: 100 ng/mL	SA-β-gal ↓; SIRT1 ↑; PGC-1α ↑; mtDNA ↑; Involucrin ↑; Loricrin ↑;	SIRT1/PGC-1α and senescence inhibition via mitochondrial biogenesis	Senomorphic
[87]	Olive polyphenols (Oleuropein (Ole) &	In Vitro: Human osteoarthritic chondrocytes. Model: Replicative senescence. Dose: 10 µM (Ole), 50 µM (HTy). In Vivo: C57BL/6J mice	SA-β-gal ↓ (in vitro & in vivo); SASP ↓ (IL-6, COX-2, MMP-13);	Senolysis of SnC and SIRT1 activation (Senomorphic/Pro-regenerative).	Dual (Senolytic + Senomorphic)

Author & Year	Compound/Extract	Study Type & Details	Key Senescence Biomarkers (Treatment Effect)	Mechanism & Pathway	Senotherapeutic Classification
	Hydroxytyrosol (HTy))	(Male, 10 wks). Model: ACLT-induced Osteoarthritis. Dose by Intra-articular injection	SIRT1 ↑; Chondrogenic markers ↑ (COL2A1, Aggrecan)		
[88]	*Rosa roxburghii* Fruit Extracts	In Vitro: HDFs & 293T cells. Model: Replicative senescence. Dose: 20 μg/mL (Optimal)	SA-β-gal ↓; p53 ↓; p21 ↓; p16 ↓ (mRNA & protein); Telomerase activity ↑ (Telomerase Reverse Transcriptase (hTERT)); ROS ↓	Telomere lengthening and antioxidant; Senomorphic (delays replicative senescence)	Senomorphic
[89]	*Rhamnoneuron balansae* (phenolic constituent)	In vitro: HDFs. Model: Replicative senescence (Old vs. Young). Dose 20 μM. Method: NMR spectroscopy and High-Resolution Electrospray Ionization Mass Spectrometry (HRESIMS) found 12 new compounds	SA-β-gal ↓ (Rhamnoneuronal C, Balansechromanones A and B); p53/p21 ↓; p16/p15 ↓ (Rhamnoneuronal C, not Balansechromanones A and B); Viability of SnCs ↓ (high selectivity index); ↑ Caspase-3/7 activity (Apoptosis)	p53/p21 pathway (Rhamnoneuronal C), and induction of Apoptosis (Caspase-3/7 activation) specifically in SnCs and Balansechromanones A and B via different pathways	Senolytic
[90]	*Zingiber officinale Roscoe* (ZOE), rhizome extract (ZTE)	In vitro: HUVECs; BV2 (murine microglia). Model: Replicative senescence (HUVECs) & LPS-induced 250 ng/mL (BV2); Dose extract: ZOE 10 μg/mL, ZTE 3 μg/mL.	SA-β-gal ↔; p16 ↑ (sHUVEC) (note: unexpected, possibly reflects senescent baseline); IL-1β ↓; TNF-α ↓; IL-8 ↓; MCP-1 ↓; ICAM-1 ↓; miR-126 ↑	NF-κB/p65, p38 MAPK, and PI3K/AKT/mTOR pathway inhibition, and miRNA regulation; senomorphic	Senomorphic
[91]	*Sonchus oleraceus* L. (pūhā) leaf extracts	In vitro: WI-38 human lung diploid fibroblasts (Coriell Cell Repositories). Senescence inducer: Single exposure to 100 μM H_2O_2 for 1 h. Dose tested: 1, 5, and 20 mg/mL. Key antioxidants identified by HPLC-DPPH: caftaric acid, chlorogenic acid, chicoric acid (chicoric acid at highest concentration)	SA-β-gal ↓ (concentration dependent): 5 mg/mL reduced H_2O_2 SA-β-gal by 2/3 ($p<0.05$); 20 mg/mL completely suppressed SA-β-gal to control level ($p<0.05$); Cellular Antioxidant Activity ↓, Cell viability >90% maintained up to 100 mg/mL.	Antioxidant-mediated SIPS suppression: peroxyl radical scavenging → intracellular ROS ↓ → oxidative stress ↓ → SA-β-gal ↓	Senomorphics
[92]	Kaempferol	In Vitro: Human endothelial cells (EA.hy926 cells). Senescence inducer: H_2O_2 300 μM. Dose kaempferol: 20 μM and 40 μM. In Vivo: ApoE−/− mouse induced with HFD. Dose kaempferol: 10 mg/kg (low) and 20 mg/kg (high)	SA-β-gal ↓; p16 ↓; p53 ↓; p21 ↓, SASP ↓ (IL-6, IL-8, TNF-α, ICAM1, CCL2 and MMP9); endothelial nitric oxide synthase (eNOS) ↑	Dose-dependent activate Nrf2/HO-1/NQO1 Pathway Activation (10 mg/kg) , synergistic antioxidant and anti-senescence effect than 20 mg/kg	Dual (Senolytic + Senomorphic)
[93]	*Nephelium lappaceum* seeds (9 isolated kaempferol-type compounds)	In vitro model: Primary HDFs (young and aged/senescent). induction model: Replicative senescence. Dose: 10 μM for all compounds (Kaempferol 3-O-β-d-glucopyranosyl-7-O-α-l-rhamnopyranoside, Kaempferol 3-O-rutinoside,	SA-β-gal ↓; p16INK4A ↓; p53 ↓; p21 ↓, SASP ↓ (IL-6, IL-8, IL-1α, IL-1β); SIRT1 ↓	Suppression of cell cycle arrest: p53/p21 pathway; SIRT1 pathway; Senomorphic activity	Dual (Senolytic + Senomorphic)

Author & Year	Compound/Extract	Study Type & Details	Key Senescence Biomarkers (Treatment Effect)	Mechanism & Pathway	Senotherapeutic Classification
		Kaempferol 7-O-α-l-rhamnopyranoside, Compound 7, novel acylated flavonol glycoside)			
[94]	*Rehmannia glutinosa* & *Astragalus membranaceus*	In Vivo: C57BL/6J female mice (20 mo). Target tissue: Hematopoietic stem cells (HSCs) from bone marrow. Dose: *R. glutinosa* 200 mg/day and *A. membranaceus* 200 mg/day.	SA-β-gal ↓; p16 ↓, p53 ↓, p21 ↓; ROS↓; B-cell immunity ↑; Lifespan ↑	ROS reduction, senescence reduction, enhanced HSC self-renewal and B-cell immunity enhancement	Senomorphic
[95]	Qijing Mingmu decoction (Formulation from *L. barbarum, Polygonatum, O. japonicus, P. cocos, Glycyrrhiza, E. prostrata* and *L. striatum*).	In Vitro: Human conjunctival fibroblasts (HCFs) derived from patients with conjunctivochalasis (CCh). Senescence inducer: TNF-α. Dose of decoction granule: 1.61 mg/L.	SA-β-gal ↓; p38α ↓; p21 ↓; p53 ↓; SMP30 ↑ (protein and mRNA). SASP ↓ (IL-1β, IL-6, IL-8 and TNF-α. Cell viability); ROS ↓; SOD ↓	Antioxidant, inhibition of the p53/p21 and p16/Rb pathways and MAPK/NF-κB signaling axis inhibition.	Senomorphic
[96]	JM10001 (*Salsola collina* extract) and JM10101 (quercetin (Q) + β-sitosterol (β) + Salicylic acid (S) - optimised 3-compound combination)	In Vitro: Human fetal lung fibroblasts (MRC-5 cells), and mouse embryonic fibroblasts (MEFs). Model: Replicative senescence. Dose: JM10001 50 mg/L and JM10101 6.2 mg/L. In Vivo: Replicative senescence (*C. elegans),* Doxorubicin 5 mg/kg intraperitoneally (C57BL/6J mice) and naturally aged mice (18 mo). Dose treatment: *C. elegans*: JM10001 200 mg/L and JM10101 0.85 mg/L (1 μM Q + 1 μM β + 1 μM S), Doxorubicin-induced mice: JM10001 1 and 2 mg/kg, JM10101 12.5 mg/kg and 50 mg/kg oral, and natural aged mice: JM10001 5 mg/kg, JM10101 6.25 mg/kg and 25 mg/kg oral).	SA-β-gal ↓; p16 ↓; p21 ↓; p53 ↓; SASP ↓ (IL-6; IL-1β)	DNA damage response mitigation, p53 signaling pathway regulation, senomorphlytic (combined senolytic + senomorphic strategy) role and antioxidant	Dual (Senolytic + Senomorphic)
[97]	*Camellia* Sect. *Chrysantha* Chang extracts (8 species); key active metabolites: Sanchakasaponin C & D, flavan-3-ols (catechin, epicatechin, PCC1,	In vitro: Rat H9c2 cardiomyocytes. Senescence inducer: D-gal; Analysis: UPLC-QTOF-MS non-targeted metabolomics (27 compounds identified)	SA-β-gal ↓; p16INK4a ↓; p21CIP1/WAF1 ↓; p53 ↓; BCL-2 ↓; Bcl-w ↓; Bcl-xL ↓; Bax ↓; BCL-2/Bax ratio ↓ (promotes apoptosis of SnCs); selective SnC viability ↓	SCAPs pathway inhibition (BCL-2 family gene down-regulation → SnC apoptosis); DDR pathway modulation; antioxidant activity (radical scavenging)	Senolytic

Author & Year	Compound/Extract	Study Type & Details	Key Senescence Biomarkers (Treatment Effect)	Mechanism & Pathway	Senotherapeutic Classification
	PCC2), flavonols (rutin, quercetin glycosides, kaempferol glycosides); EtOAc extracts 100–200 µg/mL; n-BuOH extracts 50 µg/mL				
[98]	Shea-derived amyrins (α-amyrin & β-amyrin, triterpenoids from *Vitellaria paradoxa*)	In Vitro: MEFs from wild-type C57BL/6J and and human diploid fibroblasts (TIG-1). Senescence inducer: Replicative senescence (TIG-1) and UV radiation (MEFs). Dose: α- or β-amyrin 20 µM, and triterpene fraction 780 µg/mL.	SA-β-gal positive cells ↓; p21CIP1/WAF1 ↓; Annexin V/PI+ cells apoptosis; Cell viability ↓; cannabinoid receptor 2 (Cnr2) ↑	Senolytic activity, Cnr2 signaling, and caspase-independent, β-Amyrin's potent senolytic	Senolytic
[99]	*Boesenbergia pandurata* (Fingerroot) ethanol extract.	In Vitro: BJ. Senescence inducer: Etoposide 20 µM. Dose extract: 5, 30, and 60 µg/ml.	SA-β-gal ↓; p16 ↓; p21 ↓; p53 ↓; IL-6 ↓; CD90+	Broader anti-aging and antioxidant properties in fibroblast	Dual (Senolytic + Senomorphic)
[100]	*Isatis tinctoria* (woad) 70% (v/v) ethanolic extracts	In Vivo: HDFs, BJ cells and human lung fibroblasts (IMR90). Senescence inducer: bleomycin 10 µg/ml and replicative. Dose extract: 200 and 500 parts per million (ppm).	SASP (IL-6) ↓; Apoptosis (Caspase-3 and PARP) ↑;	NF-κB pathway; mTOR pathway; Apoptosis induction; Context-dependent activity selective for skin-origin fibroblasts (e.g., BJ cells) but not IMR90 cells	Dual (Senolytic + Senomorphic)
[101]	4 plant species (*Falcaria vulgaris, Ixiolirion tataricum*, *Ajuga chamaecistus*, and *Scabiosa flavida*)	In Vitro: Rat NIH3T3 fibroblasts. Induction: H_2O_2 400 µM. Dose extract: *F. vulgaris* 151.443 µg/mL, *I. tataricum* 84.057 µg/mL, *A. chamaecistus* 112.143 µg/mL, and *S. flavida* 215.108 µg/mL.	SA-β-gal ↓; p16 ↓; p21 ↓; p53↓; IL-6 ↓; TNF-α ↓; IL-1β ↓; NF-κB ↓; ROS ↓	Apoptosis induction, antioxidant, anti-inflammatory, NF-κB signaling pathway modulation; p53/p21 and p16INK4a/Rb pathway	Dual (Senolytic + Senomorphic)
[102]	Phenolamide skeleton from *Allium hookeri* leaves — identified by UHPLC-qTOF-MS/MS	In Vitro: RAW264.7 cells, A549 human lung carcinoma epithelial cell line, and HDF. Senescence inducer: Bleomycin 5 mg/mL (A549 cells), LPS 1 µg/mL (RAW264.7 cells), and replicative (HDF). Dose extract: Isolated compound tested at 5 µM and 20 µM (RAW264.7 cells), Compounds 6-8 tested at 10 µM (HDF & A549)	SA-β-gal ↓; IL-6 ↓; SASP ↓ (IL-6, IL-8, IL-1α)	Anti-inflammatory, antioxidant and N-trans-feruloyltyramine (7) most promising SASP-inhibitory, Phenolamides, compound (6–8) share a similar skeleton to avenanthramide C (senomorphic)	Senomorphic

Author & Year	Compound/Extract	Study Type & Details	Key Senescence Biomarkers (Treatment Effect)	Mechanism & Pathway	Senotherapeutic Classification
[103]	Hydrolysed low-MW polysaccharides from *Polygoni Multiflori Radix Praeparata* — structural characterization approach	In Vitro: T lymphocytes. Senescence inducer: D-galactose 20 mg/mL. In Vivo: Naturally aged C57BL/6J mice (15 months) vs young controls (8 weeks); Model: Fast-aging TERT−/− mice (telomerase knockout). Dose: low (50 mg/kg/day) and High (200 mg/kg/day), intraperitoneal. Metabolomics	SA-β-gal ↓; p16 ↓; p21 ↓; IL-6 ↓; TNF-α ↓; IL-1β ↓;	Senolytic and senomorphic	Dual (Senolytic + Senomorphic)
[104]	*Agrimonia pilosa Ledeb*. (APL) (90% ethanol extraction, lyophilized *Agrimol* B: Pure phloroglucinol compound isolated from APL)	In Vitro: WI38 cells. Senescence inducer: Doxorubicin. Dose: APL-E mixed diet: 25 μg/mL for 3 days, Agrimol B: 0.1–3.0 μM (primary dose: 3 μM for 3 days). In Vivo: Aged mice (Male C57BL/6J mice, 82-83 weeks old), Young controls. Dose: 0.2% APL-E mixed diet (low dose), 0.4% APL-E mixed diet (high dose), 3 months of continuous feeding	SA-β-gal ↓; p16 ↓; p53 ↓; IL-6 ↓; IL-1β ↓	PI3K/AKT pathway; Apoptosis induction; Senolytic	Senolytic
[105]	Cocoa Polyphenol Extract	In vitro: BM-MSCs, isolated from young adult male Wistar albino rats. Senescence inducer: H_2O_2 100 μM, 2h for premature senescence, H_2O_2 (25 μM, 2h) for ROS induction, LPS (100 ng/mL, 12h) for inflammation induction	p21 ↓; p53 ↓; TNF-α; ROS ↑; Apoptosis ↑	AMPK pathway; NF-κB pathway; Nrf2 pathway; p53/p21 pathway	Senomorphic
[106]	*Solidago virgaurea* (Goldenrod), Ethanolic extract from dried aerial plant parts	in vitro: Primary HDFs. Model: Replicative senescence model: SIPS (Stress-Induced Premature Senescence): Chronic oxidative stress (9 treatments with 100 μM H_2O_2 for 1h each).	SA-β-gal ↓; p16 ↓; p21 ↓; ROS ↓; SASP ↓; Apoptosis ↑	NF-κB pathway; Senolytic activity; Senomorphic activity	Dual (Senolytic + Senomorphic)
[107]	Extract of *Salvia haenkei* (Bolivian prawn sage), contains luteolin and luteolin-7-O-glucuronide	In Vitro High-throughput screening using different murine and human cell models, including HaCaT cells. In Vivo: Naturally aged mice (treatment started at 20 mo). Dose xtract: 0.5 mg/kg bw, administered daily in drinking water).	SA-β-Gal ↓; p16INK4a ↓; SASP ↓	luteolin in HK disrupts the p16-CDK6 interaction, Inhibits the G1 to S phase cell cycle arrest, and; SIRT1 pathway; Antioxidant	Dual (Senolytic + Senomorphic)
[108]	Piperlongumine, (alkaloid/amide from *Piper longum* — Long Pepper)	In Vitro: Human WI-38 fibroblasts, IMR-90 fibroblasts, and HUVECs. Induction: IR, replicative exhaustion, or oncogene expression (Ras). Concentrations: ranging from 2.5 to 10 μM. In Vivo: Naturally aged mice (24 months).	Cell Viability ↓; SA-β-gal ↓; Apoptosis, Caspase-3/7 activity ↑	ROS-Dependent Proteasomal Degradation, leads to the ubiquitin-proteasomal degradation of OXR1 (Oxidation Resistance 1 protein) → Apoptosis in SnC	Senolytic

Author & Year	Compound/Extract	Study Type & Details	Key Senescence Biomarkers (Treatment Effect)	Mechanism & Pathway	Senotherapeutic Classification
		Dose: 50 mg/kg/day administered via intraperitoneal injection for 28 days.			
[109]	Gingerenone A (ginger derivative)	In Vitro: WI-38 human diploid fibroblasts (HDFs). Inducer: IR at 10 Gy. Dose: 6-shogaol (20 μM), D+Q: 250 nM D + 10 μM Q (positive control). Other ginger components tested: 6-gingerol (20 μM), 8-gingerol (100 μM), 10-gingerol (100 μM), 8-shogaol (33 μM), zingerone (100 μM)	SnC viability ↓; Selective killing of SnCs (no effect on proliferating cells); SA-β-gal ↓; SASP ↓ (IL-6, MCP-1, IP-10). Apoptosis induction ↓; cleaved caspase-3 ↓; Bcl-XL ↓	Apoptosis pathway activation	Dual (Senolytic + Senomorphic)
[110]	Gingerenone A (GinA); modified forms GinA-EPA *Zingiber officinale*	In vivo; two models: (1) aged C57BL/6JN mice (79–80 wks), oral gavage; (2) p16TdTom knock-in mice, doxorubicin-induced; analysis: UPLC-QTOF-MS plasma/liver metabolomics	p16+ cells in lung ↓ (GinA: 7.3→1.1%, $p=0.014$; modGinA: 7.3→0.8%, $p=0.0022$); IL-6 ↓ (modGinA, $p<0.05$ at washout); IL-5 ↓; NADP+ ↑ in liver; PE/PG ↑ (mitochondrial membrane remodeling); TCA cycle metabolites normalized	AMPK/SIRT1 activation; mitochondrial function restoration (TCA cycle, oxidative phosphorylation); SASP suppression (IL-6 ↓, IL-5 ↓); lipid metabolism reprogramming	Dual (Senolytic + Senomorphic
[111]	Cycloastragenol (CAG), metabolite isolated (triterpenoid saponin from *Astragalus membranaceus*)	In Vitro: IMR-90. Senescence inducer: Etoposide (VP-16) or Replicative senescence. Concentration: 50 μM and 100 μM. In Vivo: Mice with irradiation-induced aging (Total Body Irradiation)	SA-β-gal activity ↓ (In vitro and In vivo); p16INK4a ↓; p21CIP1 ↓; SASP ↓ (IL-6, IL-8, MMP-3); SnC viability ↓ (Selective apoptosis)	Induction of apoptosis, Bcl-2 family protein inhibition PI3K/AKT/mTOR signalling pathway. Senolytic mechanism	Senolytic
[112]	Astragaloside IV	In Vitro: Primary mouse astrocytes. Model/Induction: replicative senescence, LPS (500 ng/ml) or MPP^{+} (200 μM). Dose Astragaloside IV: 1, 10, 50, 100, 200 μM. In Vivo: MPTP-induced Parkinson's disease (PD) mouse model, male, 4–5 months. Dose: 100 mg/kg, intraperitoneal	SA-β-gal ↓; p16Ink4a ↓; nuclear lamin B1 ↑; SASP ↓ (IL-6, IL-1β, IL-1α, CXCL1, MMP3, MMP9); ↓ mitochondrial ROS and improved mitochondrial membrane potential	Promotion of mitophagy: ↓ p62. ↑ PINK1 and Autophagy-dependent Effects: Inhibition of astrocyte senescence and neuroprotection in PD model	Senomorphic
[113]	Pyrroloquinoline Quinone	In Vitro: BJ cells and IMR-90 cells. Senescence inducer: Replicative and H_2O_2 200 μM, Doxorubicin, etoposide	SA-β-gal ↓; p16 ↓; p21 ↓; ROS ↓; γH2A.X ↓	Nrf2 Pathway Activation, Apoptosis induction; Antioxidant	Dual (Senolytic + Senomorphic)

Author & Year	Compound/Extract	Study Type & Details	Key Senescence Biomarkers (Treatment Effect)	Mechanism & Pathway	Senotherapeutic Classification
[114]	Ginkgetin (biflavonoid from *Ginkgo biloba*)	In Vitro: MEFs with Dox and IR-induced senescence; Dose: 2 μM. In Vivo (C57BL/6J mice with Dox-induced and TBI-induced aging; Dose: 5 mg/kg	↓ p16, p21↓, IL-6↓, IL-1β↓, and SA-β-gal ↓	Senomorphic activity and inhibits cGAS-STING pathway by directly binding to STING carboxy-terminal domain	Senomorphic
[115]	Cardiac glycosides (ouabain, digitoxin, digoxin — from *Digitalis* and *Strophanthus* species)	In vitro: IMR-90, primary bronchial epithelial cells (PBEC), Various cancer cell lines (SKHep1, A549, HCT116). Induction: oncogene and IR (6 Gy). Dose: 1 mg/kg (Ouabain and Digitoxin), Ouabain: 10–100 nM, Digoxin: ~100 nM, Digitoxin: 20–33 nM, Bufalin, ouabagenin, and strophanthidin, intraperitoneal, intermittent dosing	SA-β-gal ↓; p16 ↓; p21 ↓; Apoptosis ↓; caspase-3/7 ↑	Apoptosis induction (senolytic); Pathway: CG → Na^{+}/K^{+}-ATPase inhibition → ion imbalance → JNK/p38/GSK3 → apoptosis	Senolytic
[116]	Eleutheroside E	In Vitro: Human Skin Fibroblasts (HSFs) Inducer: D-gal. Concentration: Dose-dependent	SA-β-gal ↓; ROS, ↓; MDA↓ ; ↑ SOD, CAT; ↓ Apoptosis (Bax/Bcl-2); ↓ PI3K/AKT phosphorylation;	PI3K/AKT pathway inhibition; attenuation of oxidative stress; Senomorphic	Senomorphic
[117]	Hesperetin	In Vitro: A549 cells. Senescence inducer: Bleomycin. In Vivo: C57BL/6 mice. Induction: Bleomycin (Pulmonary Fibrosis model). Dose: 100, 200 mg/kg/day (oral)	SA-β-gal ↓; p21 ↓; SASP ↓ (IL-6, TNF-α); Collagen deposition ↓ (Fibrosis); ↑ Autophagy	CISD2/BECN1 pathway (restores impaired autophagy); Nrf2 activation; Senomorphic (Anti-fibrotic/Anti-senescence)	Senomorphic
[118]	Indole-3-Carbinol (I3C) (glucosinolate metabolite from cruciferous vegetables)	In Vitro: MEFs and Human Fibroblasts (primary). Model: Replicative senescence	SA-β-gal ↓; Apoptosis ↑	Induction of apoptosis in SnCs (Senolytic)	Senolytic
[119]	Rosmarinic Acid (phenolic acid from *Salvia rosmarinus* and others)	In Vitro: BV2 Microglial cells. Senescence inducer: LPS (repeated stimulation) Concentration: 1 μM In Vivo: Mice (Spared Nerve Injury model) Dose: 5 mg/kg (oral)	SA-β-gal ↓; SASP ↓ (IL-1β release); SAHF ↓ (Senescence-Associated Heterochromatin Foci)	Anti-inflammatory; reduction of microglial activation and oxidative stress. Senomorphic (Reduces senescence markers and neuroinflammation)	Senomorphic
[120]	4-methoxychalcone and 4-bromo-4'-methoxychalcone	In Vitro: Human Aortic Endothelial Cells (HAEC) & IMR-90 Fibroblasts. Senescence inducer: Replicative senescence. Concentration: (IC50 values determined)	SnCs viability ↓ (Selective cytotoxicity: Selectivity Index (SI) ↑ for senescent vs. young cells)	Selective cytotoxicity (Structure-Activity Relationship: 4-OMe substitution enhances selectivity).<br>Senolytic	Senolytic

Author & Year	Compound/Extract	Study Type & Details	Key Senescence Biomarkers (Treatment Effect)	Mechanism & Pathway	Senotherapeutic Classification
[121]	Magnolin (lignan from *Magnolia biondii* flower)	In vitro: HSFs. Ex vivo: human skin, clinical trial: n=30, 56 days	Wrinkles ($\downarrow$ 11.37%); skin glossiness ($\uparrow$ 15.26%); roughness ($\downarrow$ 22.94%); elasticity ($\uparrow$ 12.71%); p16 $\downarrow$; p21 $\downarrow$; SA-β-gal $\downarrow$; MMPs $\downarrow$; collagens $\uparrow$	Senomorphic; Targets CXCL10-induced inflammaging; Inhibits CXCL10/CXCR3/p38 pathway, suppresses p38 phosphorylation	Senomorphic
[122]	Isovitexin	In vitro: HaCaT; Senescence inducer: AAPH (2,2′-azobis-2-methyl-propanimidamide), 1 mM; Dose Iso: 20–40 μg/mL. In vivo: Female Kunming mice. Senescence inducer: UVB irradiation, UVB dose escalated from 60 mJ/cm² (weeks 1–2) to 240 mJ/cm² (weeks 5–8), cumulative dose ~6.9 J/cm²; Iso: 25 and 50 mg/kg oral daily for 8 wks	SA-β-gal $\downarrow$; SAHF +cells $\downarrow$; p21CIP1 $\downarrow$; p16INK4a; SASP $\downarrow$ (IL-6, IL-1β, TNF-α, MMP-1 and MMP-3); Oxidative damage markers (MDA) $\downarrow$; Antioxidant enzymes (SOD, CAT, GSH-Px) $\downarrow$	PI3K/AKT pathway, lipid metabolic pathway remodeling including fatty acid biosynthesis, linoleic acid, α-linolenic acid metabolism, and unsaturated fatty acid biosynthesis (untargeted LC-MS/MS), NF-κB-driven SASP suppression; Nrf2/antioxidant enzyme axis activation; MAPK pathway modulation	Dual (Senolytic + Senomorphic)
[123]	Caffeic acid	In Vitro: A549 Cells and BEAS-2B Cells. Senescence inducer: A549 cells: 15 μM cisplatin; BEAS-2B cells: UVA radiation. Dose: 3 μM to 100 μM . In Vivo: Bleomycin-induced pulmonary fibrosis balb/c mouse model. Dose CA: 10 mg/kg orally × 3 wks	In Vitro: SASP $\downarrow$ (IL-6, TNF-α, IL-1β); PKCO and ANXA5 $\downarrow$. In Vivo: SASP $\downarrow$ (IL-6, TNF-α, IL-1β)	NF-κB Pathway Inhibition, reduced SASP secretion, and alleviation of lung fibrosis by targeting the ANXA5-PKCθ-NF-κB signaling axis.	Senomorphic
[124]	*Polygonatum kingianum* saponins (PkS); crude saponin extract from *Polygonatum kingianum* rhizome	In vitro: WI-38 human lung fibroblasts. Senescence inducer: H_2O_2-SIPS (200 μM, 2 h). PkS treatment: 0, 50, 100, 200 μg/mL for 24 h post-induction. In vivo: *C. elegans* wild-type N2; lifespan and healthspan assays. PkS dose at 0.25, 0.5, 1.0 mg/mL from L4 stage.	SA-β-gal $\downarrow$; SASP $\downarrow$ (IL-6 $\downarrow$, IL-8 $\downarrow$, MMP-1 $\downarrow$); p21 $\downarrow$; p16 $\downarrow$; γH2AX foci $\downarrow$; lifespan $\uparrow$; body bending frequency $\uparrow$; pharyngeal pumping rate $\uparrow$; lipofuscin $\downarrow$; ROS $\downarrow$; SOD $\uparrow$	ASP suppression via NF-κB pathway inhibition; sir-2.1/autophagy pathway activation (*C. elegans* SIRT1 orthologue); FoxO transcription factor nuclear translocation $\uparrow$; autophagic flux $\uparrow$ (LC3-II/LC3-I ratio $\uparrow$, p62 $\downarrow$)	Senomorphic
[125]	*Pleurotus eryngii* (king oyster mushroom)<br>- Bioactive peptides (food-derived)	In vitro: PC12 cells (rat pheochromocytoma). Senescence inducer: D-gal-induced senescent; Analysis: integrated transcriptomics (RNA-seq, DESeq2) + untargeted metabolomics (LC-MS/MS, UHPLC-Q Exactive HF-X)	SA-β-gal $\downarrow$ (39.86%); ROS $\downarrow$ (51.53%); SOD $\uparrow$; CAT $\uparrow$; GSH-Px $\uparrow$; MDA $\downarrow$ (27.11%); Arap3 $\downarrow$; Grin2d $\downarrow$; Npy1r $\uparrow$; TCA cycle restored; PPP/NADPH $\uparrow$	TLR4/NF-κB/MAPK pathway suppression; TCA cycle restoration; pentose phosphate pathway (PPP) $\rightarrow$ NADPH $\uparrow$; cAMP signaling; glutamatergic synapse modulation	Senomorphic
[126]	Betaine (trimethylglycine; primary intervention, 2 mM optimal); quercetin (100 μM)	In vivo: C. elegans wild-type N2 and human serum validation (n=100, age 23–77); NMR metabolomics (¹H 600-MHz, CPMG); RNA-seq; electron microscopy (autophagy)	Lipofuscin $\downarrow$; ROS $\downarrow$ 30%; SOD $\uparrow$ 1.3×; lifespan $\uparrow$ +114% (betaine 2 mM, $p<0.0001$); autophagosome $\uparrow$; rheb-1 $\downarrow$; let-363 $\downarrow$; aak-2 $\uparrow$; daf-16 $\uparrow$; akt-1 $\downarrow$; akt-2 $\downarrow$; sgk-1 $\downarrow$;	AMPK/mTOR (autophagy induction); FoxO/DAF-16 (oxidative stress response); p38-MAPK; betaine metabolism cascade (choline $\rightarrow$ betaine $\rightarrow$ DMG (TCA cycle / ATP synthesis) $\rightarrow$ glycine); methyl donor activity	Senomorphic / Anti-aging

Author & Year	Compound/Extract	Study Type & Details	Key Senescence Biomarkers (Treatment Effect)	Mechanism & Pathway	Senotherapeutic Classification
			betaine ↓ with age (C. elegans + human serum)		
[127]	SBT (Srolo Bzhtang), Tibetan polyherbal formulation comprising *Solms-laubachia eurycarpa*, *Bergenía purpurascens*, *Laccifera lacca* and *Glycyrrhiza uralensis*	In vitro: H9c2 cardiomyocytes. Senescence inducer: D-gal 50 mg/mL for 24 h. SBT treatment doses: 50, 100, 200, 300, and 400 µg/mL. In vivo: AB wild-type zebrafish. Senescence inducer: H_2O_2 at 5 mM in embryo water. SBT treatment doses: 10, 20, and 30 µg/mL in embryo water	SA-β-gal ↓ (41.2% → 20%), cell viability ↑, p53↓, p21↓, SIRT1↑ (both models, protein + mRNA); Bax↓, Bcl-2↑ (anti-apoptotic: TUNEL↓ in H9c2; AO↓ in zebrafish); NF-κB↓, MAPK↓ (zebrafish qPCR); SOD↑, CAT↑, GSH↑, MDA↓ (zebrafish antioxidant); zebrafish: survival rate ↑, heart rate restored	p53/p21/SIRT1 axis (primary); Bax/Bcl-2 apoptosis pathway; NF-κB/MAPK (anti-inflammatory); Antioxidant defence (SOD/CAT/GSH)	Senomorphic

Note: ↓ indicates decrease; ↑ indicates increase; ↔ indicates unchanged. . All biomarker changes reflect treatment effects relative to untreated senescent cell controls unless otherwise specified.

Abbreviations: SnC, senescent cell; SASP, senescence-associated secretory phenotype; SIPS, stress-induced premature senescence; SCAPs, senescent cell anti-apoptotic pathways; D+Q, dasatinib plus quercetin; HDFs, human dermal fibroblasts; HUVECs, human umbilical vein endothelial cells; MSCs, mesenchymal stem cells; BMSCs, bone marrow MSCs; hBMSCs, human BMSCs; HSCs, hematopoietic stem cells; NPCs, nucleus pulposus cells; ECs, endothelial cells; VSMCs, vascular smooth muscle cells; MEFs, mouse embryonic fibroblasts; D-gal, D-galactose; HFD, high-fat diet; IR, ionizing radiation; TBI, total body irradiation; OA, osteoarthritis; IVD, intervertebral disc; IVDD, IVD degeneration; UUO, unilateral ureteral obstruction; GNPS, Global Natural Product Social Molecular Networking; PD, population doubling; NA, not reported.

References

1. López-Otín C, Blasco MA, Partridge L, Serrano M, Kroemer G. Hallmarks of aging: An expanding universe. Cell 2023; 186:243–78. https://doi.org/10.1016/j.cell.2022.11.001.
2. Kudlova, N., De Sanctis, J. B., & Hajduch, M. (2022). Cellular senescence: Molecular targets, biomarkers, and senolytic drugs. International Journal of Molecular Sciences, 23(8), 4168. https://doi.org/10.3390/ijms23084168
3. Klepacki H, Kowalczuk K, Łepkowska N, Hermanowicz JM. Molecular Regulation of SASP in Cellular Senescence: Therapeutic Implications and Translational Challenges. Cells. 2025;14(13):1094. https://doi.org/10.3390/cells14130942
4. Zhu Y, Tchkonia T, Fuhrmann-Stroissnigg H, Dai HM, Ling YY, Stout MB, et al. Identification of a novel senolytic agent, navitoclax, targeting the Bcl-2 family of anti-apoptotic factors. Aging Cell 2016;15:428–35. https://doi.org/10.1111/acel.12445.
5. Yousefzadeh MJ, Zhu Y, McGowan SJ, Angelini L, Fuhrmann-Stroissnigg H, Xu M, et al. Fisetin is a senotherapeutic that extends health and lifespan. EBioMedicine 2018;36:18–28. https://doi.org/10.1016/j.ebiom.2018.09.015.
6. Zhang L, Pitcher LE, Prahalad V, Niedernhofer LJ, Robbins PD. Targeting cellular senescence with senotherapeutics: senolytics and senomorphics. FEBS Journal 2023;290:1362–83. https://doi.org/10.1111/febs.16350.
7. Ashkenazi A, Fairbrother WJ, Leverson JD, Souers AJ. From basic apoptosis discoveries to advanced selective BCL-2 family inhibitors. Natural Review Drug Discovery 2017;16:273–84. https://doi.org/10.1038/nrd.2016.253.

8. Ziff OJ, Kotecha D. Digoxin: the good and the bad. Trends Cardiovascular Medicine. 2016;26(7):585–595. https://doi.org/10.1016/j.tcm.2016.03.011
9. Newman DJ, Cragg GM. Natural Products as Sources of New Drugs over the Nearly Four Decades from 01/1981 to 09/2019. Journal of Natural Product 2020;83:770–803. https://doi.org/10.1021/acs.jnatprod.9b01285.
10. Gurău F, Baldoni S, Prattichizzo F, Espinosa E, Amenta F, Procopio AD, et al. Anti-senescence compounds: A potential nutraceutical approach to healthy aging. Ageing Research Rev 2018;46:14–31. https://doi.org/10.1016/j.arr.2018.05.001.
11. Chen Z, Wu Y, Lin Q, Cai J, Liu X, Liang Y. Nutrition Interventions of Herbal Compounds on Cellular Senescence. Oxid Med Cell Longevity. 2022; 2022:1059257. https://doi.org/10.1155/2022/1059257
12. Mbara KC, Devnarain N, Owira PMO. Potential Role of Polyphenolic Flavonoids as Senotherapeutic Agents in Degenerative Diseases and Geroprotection. Pharmaceutical Medicine 2022;36:331–52. https://doi.org/10.1007/s40290-022-00444-w.
13. Sun Y, Wang C, Wen L, Ling Z, Xia J, Cheng B, et al. Quercetin ameliorates senescence and promotes osteogenesis of BMSCs by suppressing the repetitive element-triggered RNA sensing pathway. International Journal of Molecular Medicine. 2025;55:e5445. https://doi.org/10.3892/ijmm.2024.5445
14. Shao Z, Wang B, Shi Y, Xie C, Huang C, Chen B, et al. Senolytic agent quercetin ameliorates intervertebral disc degeneration via the Nrf2/NF-κB axis. Osteoarthritis Cartilage. 2021;29(3):413–422. https://doi.org/10.1016/j.joca.2020.11.006
15. Giordani C, Matacchione G, Giuliani A, Valli D, Scarpa ES, Antonelli A, et al. Pro-osteogenic and anti-inflammatory synergistic effect of orthosilicic acid, vitamin K2, curcumin, polydatin and quercetin combination in young and senescent bone marrow-derived mesenchymal stromal cells. International Journal of Molecular Science. 2023;24(10):8820. https://doi.org/10.3390/ijms24108820
16. Deleray V, Dorrestein PC, Avalon NE. Molecules to medicine: advances in metabolomics for natural product drug discovery. Current Opinion in Biotechnology. 2025;93:103318. https://doi.org/10.1016/j.copbio.2025.103318
17. Cho MH, Jin H, Ha J, Chu S, An S. An integrated systems pharmacology approach combining bioinformatics, untargeted metabolomics and molecular dynamics to unveil the anti-aging mechanisms of *Tephroseris flammea*. Biomolecules. 2025;15:1740. https://doi.org/10.3390/biom15121740
18. Peters, M. D. J., Marnie, C., Tricco, A. C., Pollock, D., Munn, Z., Alexander, L., McInerney, P., Godfrey, C. M., & Khalil, H. (2020). Updated methodological guidance for the conduct of scoping reviews. JBI Evidence Synthesis, *18*(10), 2119–2126. https://doi.org/10.11124/JBIES-20-00167
19. Tricco, A. C., Lillie, E., Zarin, W., O'Brien, K. K., Colquhoun, H., Levac, D., Moher, D., Peters, M. D. J., Horsley, T., Weeks, L., Hempel, S., Akl, E. A., Chang, C., McGowan, J., Stewart, L., Hartling, L., Aldcroft, A., Wilson, M. G., Garritty, C., … Straus, S. E. (2018). PRISMA extension for scoping reviews (PRISMA-ScR): Checklist and explanation. Annals of Internal Medicine, 169*(7), 467–473.* https://doi.org/10.7326/M18-0850
20. Hooijmans, C. R., Rovers, M. M., de Vries, R. B. M., Leenaars, M., Ritskes-Hoitinga, M., & Langendam, M. W. (2014). SYRCLE's risk of bias tool for animal studies. BMC Medical Research Methodology, *14*, 43. https://doi.org/10.1186/1471-2288-14-43
21. Zoico E, Nori N, Darra E, Tebon M, Rizzatti V, Policastro G, et al. Senolytic effects of quercetin in an in vitro model of pre-adipocytes and adipocytes induced senescence. Scientific Reports. 2021;11:23237. https://doi.org/10.1038/s41598-021-02544-0
22. Geng LL, Liu ZP, Zhang WQ, Li W, Wu ZM, Wang W, et al. Chemical screen identifies a geroprotective role of quercetin in premature aging. Protein & Cell. 2019;10(6):417–435. https://doi.org/10.1007/s13238-018-0567-y
23. Kim SR, Jiang K, Ogrodnik M, Chen XJ, Zhu XY, Lohmeier H, et al. Increased renal cellular senescence in murine high-fat diet: Effect of the senolytic drug quercetin. Translational Research. 2019;213:112–123. https://doi.org/10.1016/j.trsl.2019.07.005
24. Lewinska A, Adamczyk-Grochala J, Bloniarz D, Olszowka J, Kulpa-Greszta M, Litwinienko G, et al. AMPK-mediated senolytic and senostatic activity of quercetin surface functionalized Fe3O4 nanoparticles during oxidant-induced senescence in human fibroblasts. Redox Biology. 2020;28:101337. https://doi.org/10.1016/j.redox.2019.101337
25. Kim SG, Sung JY, Kim JR, Choi HC. Quercetin-induced apoptosis ameliorates vascular smooth muscle cell senescence through AMP-activated protein kinase signaling pathway. Korean Journal of Physiology & Pharmacology. 2020;24(1):69–79. https://doi.org/10.4196/kjpp.2020.24.1.69

26. Jiménez R, Zúñiga-Muñoz A, Álvarez-León E, García-Niño WR, Navarrete-Anastasio G, Soria-Castro E, et al. Quercetin preserves mitochondria-endoplasmic reticulum contact sites improving mitochondrial dynamics in aged myocardial cells. Biogerontology. 2024;26:29. https://doi.org/10.1007/s10522-024-10174-y
27. Matacchione G, Valli D, Silvestrini A, Giuliani A, Sabbatinelli J, Giordani C, et al. Curcumin, polydatin and quercetin synergistic activity protects from high-glucose-induced inflammation and oxidative stress. Antioxidants. 2022;11(6):1037. https://doi.org/10.3390/antiox11061037
28. Liu HX, Xu QX, Wufuer H, Li Z, Sun R, Jiang ZR, et al. Rutin is a potent senomorphic agent to target senescent cells and can improve chemotherapeutic efficacy. Aging Cell. 2024;23(3):e13921. https://doi.org/10.1111/acel.13921
29. Murray KO, Mahoney SA, Ludwig KR, Miyamoto-Ditmon JH, VanDongen NS, Banskota N, et al. Intermittent supplementation with fisetin improves physical function and decreases cellular senescence in skeletal muscle with aging. Aging Cell. 2025;24:e70114. https://doi.org/10.1111/acel.70114
30. Hambright S, Kawakami Y, Mu X, Gao X, Lu A, Kirkland, et al. The senolytic drug fisetin mitigates age-related bone density loss in the progeroid mouse model Zmpste24−/−. The FASEB Journal. 2020;34(S1):1. https://doi.org/10.1096/fasebj.2020.34.s1.06658
31. Zhao R, Kou H, Jiang D, Wang, F. Exploring the anti-aging effects of fisetin in telomerase-deficient progeria mouse model. PeerJ. 2023;11:e16463. https://doi.org/10.7717/peerj.16463
32. Mahoney SA, Venkatasubramanian R, Darrah MA, Ludwig KR, Vandongen NS, Greenberg NT, et al. Intermittent supplementation with fisetin improves arterial function in old mice by decreasing cellular senescence. Aging Cell. 2024;23(3):e14060. https://doi.org/10.1111/acel.14060
33. Ji XM, Dong XX, Li JP, Tai GJ, Qiu S, Wei W, et al. Fisetin clears senescent cells through the PI3K-AKT-BCL-2/BCL-XL pathway to alleviate diabetic aortic aging. Phytotherapy Research. 2025;39(6):2757–2775. https://doi.org/10.1002/ptr.8507
34. Ashiqueali SA, Chaudhari D, Zhu X, Noureddine S, Siddiqi S, Garcia DN, et al. Fisetin modulates the gut microbiota alongside biomarkers of senescence and inflammation in a DSS-induced murine model of colitis. Geroscience. 2024;46(3):3085–3103. https://doi.org/10.1007/s11357-024-01060-z
35. Zhu Y, Doornebal EJ, Pirtskhalava T, Giorgadze N, Wentworth M, Fuhrmann-Stroissnigg H, et al. New agents that target senescent cells: The flavone, fisetin, and the BCL-XL inhibitors, A1331852 and A1155463. Aging. 2017;9(3):955–963. https://doi.org/10.18632/aging.101202
36. Wang XZ, Li XY, Zhou JL, Lei Z, Yang XM. Fisetin suppresses chondrocyte senescence and attenuates osteoarthritis progression by targeting sirtuin 6. Chemico-Biological Interactions. 2024;390:110890. https://doi.org/10.1016/j.cbi.2024.110890
37. Ijima S, Saito Y, Nagaoka K, Yamamoto S, Sato T, Miura N, et al. Fisetin reduces the senescent tubular epithelial cell burden and also inhibits proliferative fibroblasts in murine lupus nephritis. Frontiers in Immunology. 2022;13:960601. https://doi.org/10.3389/fimmu.2022.960601
38. Alsuraih M, O'Hara SP, Woodrum JE, Pirius NE, LaRusso NF. Genetic or pharmacological reduction of cholangiocyte senescence improves inflammation and fibrosis in the Mdr2−/− mouse. JHEP Reports. 2021;3(3):100250. https://doi.org/10.1016/j.jhepr.2021.100250
39. Takaya K, Kishi, K. Combined dasatinib and quercetin treatment contributes to skin rejuvenation through selective elimination of senescent cells in vitro and in vivo. Biogerontology. 2024;25(4):691–704. https://doi.org/10.1007/s10522-024-10103-z
40. Camell CD, Yousefzadeh MJ, Zhu Y, Prata LGPL, Huggins MA, Pierson M, et al. Senolytics reduce coronavirus-related mortality in old mice. Science. 2021;373(6552):e. https://doi.org/10.1126/science.abe4832
41. Zhu Y, Prata LGPL, Gerdes EOW, Netto JME, Pirtskhalava T, Giorgadze N, et al. Orally-active, clinically-translatable senolytics restore α-Klotho in mice and humans. EBioMedicine. 2022;77:103912. https://doi.org/10.1016/j.ebiom.2022.103912
42. Hense JD, Garcia DN, Zanini BM, Barreto MM, Perreira GC, Isola JVV, et al. MASLD does not affect fertility and senolytics fail to prevent MASLD progression in male mice. Scientific Reports. 2024;14:17332. https://doi.org/10.1038/s41598-024-67697-0
43. Fang Y, Medina D, Stockwell R, McFadden S, Quinn K, Peck MR, et al. Sexual dimorphic metabolic and cognitive responses of C57BL/6 mice to fisetin or dasatinib and quercetin cocktail oral treatment. GeroScience. 2023;45(5):2835–2850. https://doi.org/10.1007/s11357-023-00843-0
44. Jacob J, Aggarwal A, Bhattacharyya S, Sahni D, Sharma V, Aggarwal, et al. Fisetin and resveratrol exhibit senotherapeutic effects and suppress cellular senescence in osteoarthritic cartilage-derived chondrogenic progenitor cells. European Journal of Pharmacology. 2025;997:177573. https://doi.org/10.1016/j.ejphar.2025.177573

45. Wang Y, He Y, Rayman MP, Zhang, J. Prospective selective mechanism of emerging senolytic agents derived from flavonoids. Journal of Agricultural and Food Chemistry. 2021;69(44):12418–12423. https://doi.org/10.1021/acs.jafc.1c04379
46. Flores-Torres RP, Salas-Venegas V, Santín-Márquez R, De la Vega-Tinajar M, Ramírez-Carreto RJ, Librado-Osorio R, et al. Senotherapy as a multitarget intervention in chronic obesity: Modulation of senescence, neuroinflammation, dysbiosis, and synaptic integrity in middle-aged female Wistar rats. Experimental Neurology. 2025;392:115331. https://doi.org/10.1016/j.expneurol.2025.115331
47. Eren MK, Kilincli A, Eren, Ö. Resveratrol induced premature senescence is associated with DNA damage mediated SIRT1 and SIRT2 down-regulation. PLOS ONE. 2015;10(4):e0124837. https://doi.org/10.1371/journal.pone.0124837
48. Zhang XY, Zhu CY, Zhang LY, Tan LY, Cheng WL, Li M, et al. RNA m6A methylation regulatory mechanism of resveratrol in premature senescence cells. Food Science & Nutrition. 2024;12(12):9238–9251. https://doi.org/10.1002/fsn3.4487
49. Zeng XL, Yang XN, Liu XJ. Resveratrol attenuates cigarette smoke extract induced cellular senescence in human airway epithelial cells by regulating the miR-34a/SIRT1/NF-κB pathway. Medicine. 2022;101(40):e31944. https://doi.org/10.1097/MD.0000000000031944
50. Ali D, Chen L, Kowal JM, Okla M, Manikandan M, AlShehri M, et al. Resveratrol inhibits adipocyte differentiation and cellular senescence of human bone marrow stromal stem cells. Bone. 2020;133:115252. https://doi.org/10.1016/j.bone.2020.115252
51. Wang J, Huang C, Lin Z, Pan X, Chen J, Zheng G, et al. Polydatin suppresses nucleus pulposus cell senescence, promotes matrix homeostasis and attenuates intervertebral disc degeneration in rats. Journal of Cellular and Molecular Medicine. 2018;22(11):5720–5731. https://doi.org/10.1111/jcmm.13848
52. Zhu RZ, Li BS, Gao SS, Seo JH, Choi BM. Luteolin inhibits H2O2-induced cellular senescence via modulation of SIRT1 and p53. Korean Journal of Physiology & Pharmacology. 2021;25(4):297–305. https://doi.org/10.4196/kjpp.2021.25.4.297
53. Yan Y, Huang H, Su T, Huang W, Wu X, Chen X, et al. Luteolin mitigates photoaging caused by UVA-induced fibroblast senescence by modulating oxidative stress pathways. International Journal of Molecular Sciences. 2025;26(5):1809. https://doi.org/10.3390/ijms26051809
54. Bai H, Jin H, Liu T, Yin Y, Wang H, Ruan S, et al. Luteolin alleviates vascular senescence through retinoic acid–peroxisome proliferator-activated receptor signaling and lipid metabolism remodeling combined with multi-omics analysis. Nutrients. 2025;17(22):3607. https://doi.org/10.3390/nu17223607
55. Chu JJ, Hu H, Xu W, Lu XY, Zhou HL, Hao MQ, et al. Top-down engineered micron-cell derived nanovesicles encapsulating curcumin targeting the 3R strategy (remove, remodel, repair) for Alzheimer's disease therapy. Materials Today Bio. 2025;34:102157. https://doi.org/10.1016/j.mtbio.2025.102157
56. Yang L, Shi J, Wang X, Zhang, R. Curcumin alleviates D-galactose-induced cardiomyocyte senescence by promoting autophagy via the SIRT1/AMPK/mTOR pathway. Evidence-Based Complementary and Alternative Medicine. 2022;2022:2990843. https://doi.org/10.1155/2022/2990843
57. Lee J, Kim YS, Kim E, Kim Y, Kim, Y. Curcumin and hesperetin attenuate D-galactose-induced brain senescence in vitro and in vivo. Nutrition Research and Practice. 2020;14(5):438–452. https://doi.org/10.4162/nrp.2020.14.5.438
58. Choi E, Cho Y, Kim M, Jin H, Yoo Y, Oh WK, et al. Rutaecarpine, isolated from Evodia rutaecarpa, inhibits epithelial-mesenchymal transition and cellular senescence in a mouse model of pulmonary fibrosis. Natural Product Sciences. 2024;30(3):190–197. https://doi.org/10.20307/nps.2024.30.3.190
59. Ali D, Okla M,Abuelreich S, Vishnubalaji R, Ditzel N, Hamam R, et al. Apigenin and rutaecarpine reduce the burden of cellular senescence in bone marrow stromal stem cells. Frontiers in Endocrinology. 2024;15:1360054. https://doi.org/10.3389/fendo.2024.1360054
60. Perrott KM, Wiley CD, Desprez P-Y, Campisi, J. Apigenin suppresses the senescence-associated secretory phenotype and paracrine effects on breast cancer cells. GeroScience. 2017;39(2):161–173. https://doi.org/10.1007/s11357-017-9970-1
61. Zhang HW, Xu QX, Jiang ZR, Sun R, Wang Q, Liu SH, et al. Targeting senescence with apigenin improves chemotherapeutic efficacy and ameliorates age-related conditions in mice. Advanced Science. 2025;12:e2412950. https://doi.org/10.1002/advs.202412950
62. Li BS, Zhu RZ, Lim SH, Seo JH, Choi BM. Apigenin alleviates oxidative stress-induced cellular senescence via modulation of the SIRT1-NAD+-CD38 axis. American Journal of Chinese Medicine. 2021;49(5):1235–1250. https://doi.org/10.1142/S0192415X21500592

63. Wei H, Liu Y, Huang C, Wang C, Jiang H, Wang L, et al. Ginsenoside Rg1 targets TLR2 to inhibit the NF-κB signaling pathway and ameliorate hematopoietic support of mesenchymal stromal cells. Journal of Ethnopharmacology. 2025;349:119917. https://doi.org/10.1016/j.jep.2025.119917
64. Lee DY, Arndt J, O'Connell JF, Egan JM, Kim, Y. Red ginseng attenuates the hepatic cellular senescence in aged mice. Biology. 2024;13(1):36. https://doi.org/10.3390/biology13010036
65. Lee SJ, Lee DY, O'Connell JF, Egan JM, Kim, Y. Black ginseng ameliorates cellular senescence via p53-p21/p16 pathway in aged mice. Biology. 2022;11(8):1108. https://doi.org/10.3390/biology11081108
66. Kumar R, Sharma A, Padwad Y, Sharma, R. Preadipocyte secretory factors differentially modulate murine macrophage functions during aging which are reversed by the application of phytochemical EGCG. Biogerontology. 2020;21(3):325–343. https://doi.org/10.1007/s10522-020-09861-3
67. Kumar R, Sharma A, Kumari A, Gulati A, Padwad Y, Sharma, et al. Epigallocatechin gallate suppresses premature senescence of preadipocytes by inhibition of PI3K/Akt/mTOR pathway and induces senescent cell death by regulation of Bax/Bcl-2 pathway. Biogerontology. 2019;20(2):171–189. https://doi.org/10.1007/s10522-018-9785-1
68. Lilja S, Oldenburg J,Pointner A, Dewald L, Lerch M, Hippe B, et al. Epigallocatechin gallate effectively affects senescence and anti-SASP via SIRT3 in 3T3-L1 preadipocytes in comparison with other bioactive substances. Oxidative Medicine and Cellular Longevity. 2020;2020:4793125. https://doi.org/10.1155/2020/4793125
69. Gan Y, Wang K, Chen X, Li Y, He Y, Zhou Y, et al. Senolytic procyanidin C1 alleviates renal fibrosis by promoting apoptosis of senescent renal tubular epithelial cells. The FASEB Journal. 2025;39:e70362. https://doi.org/10.1096/fj.202402558R
70. Xu QX, Fu Q, Li Z, Liu HX, Wang Y, Lin X, et al. The flavonoid procyanidin C1 hassenotherapeutic activity and increases lifespan in mice. Nature Metabolism. 2021;3(12):1706–1726. https://doi.org/10.1038/s42255-021-00491-8
71. Liu X, Liu Y, Gao Y, Zhang C, Gu C,Lv J, et al. Single-cell profiling unveils a geroprotective role of procyanidin C1 in hematopoietic immune system via senolytic and senomorphic effects. NPJ Aging. 2025;11:22. https://doi.org/10.1038/s41514-025-00222-3
72. Dang Y, An Y, He J, Huang B, Zhu J, Gao M, et al. Berberine ameliorates cellular senescence and extends the lifespan of mice via regulating p16 and cyclin protein expression. Aging Cell. 2020;19(3):e13060. https://doi.org/10.1111/acel.13060
73. Chen RS, Zhang XB, Zhu XT, Wang CS, Xu WH. Myricetin alleviated hydrogen peroxide-induced cellular senescence of nucleus pulposus cell through regulating SERPINE1. Journal of Orthopaedic Surgery and Research. 2023;18:45. https://doi.org/10.1186/s13018-022-03463-0
74. Wei J, Tan Z, Huang G, Zeng Y, Chen S, Yuan G, et al. Anthocyanins delay D-galactose-induced mouse liver aging by regulating the NF-κB/IKK signaling pathway. Food Science & Nutrition. 2025;13:e70161. https://doi.org/10.1002/fsn3.70161
75. Hu XQ, Yang YM, Tang S, Chen QY, Zhang MY, Ma JY, et al. Anti-aging effects of anthocyanin extracts of Sambucus canadensis caused by targeting mitochondrial-induced oxidative stress. International Journal of Molecular Sciences. 2023;24(2):1528. https://doi.org/10.3390/ijms24021528
76. Lee G-H, Hoang T-H, Jung E-S, Jung S-J, Han S-K, Chung M-J, et al. Anthocyanins attenuate endothelial dysfunction through regulation of uncoupling of nitric oxide synthase in aged rats. Aging Cell. 2020;19(3):e13279. https://doi.org/10.1111/acel.13279
77. Liu Y, Hong T,Lv M, Guo X, Zhang P, Yan A, et al. Delphinidin attenuates cognitive deficits and pathology of Alzheimer's disease by preventing microglial senescence via AMPK/SIRT1 pathway. Alzheimer's Research & Therapy. 2025;17:83. https://doi.org/10.1186/s13195-025-01783-x
78. Zhao X, Zhou S, Liu Y, Gong C, Xiang L, Li S, et al. Parishin alleviates vascular ageing in mice by upregulation of Klotho. Journal of Cellular and Molecular Medicine. 2023;27(9):1398–1409. https://doi.org/10.1111/jcmm.17740
79. Zhou S, Zhao X, Wu L, Yan R, Sun L, Zhang Q, et al. Parishin treatment alleviates cardiac aging in naturally aged mice. Heliyon. 2023;9(11):e22970. https://doi.org/10.1016/j.heliyon.2023.e22970
80. Tan Y, Hu A, Lu J, Lin Y, Li X, Yamaguchi T, et al. Protective effects of Centella asiatica against senescence and apoptosis in epidermal cells. Biology. 2025;14(2):202. https://doi.org/10.3390/biology14020202
81. You J, Woo J, Roh K-B, Jeon K, Jang Y, Choi S, et al. Evaluation of efficacy of Silybum marianum flower extract on the mitigating hair loss in vitro and in vivo. Journal of Cosmetic Dermatology. 2024;23(2):529–542. https://doi.org/10.1111/jocd.15978

82. Woo J, Shin S, Cho E, Ryu D,Garandeau D, Chajra H, et al. Senotherapeutic-like effect of Silybum marianum flower extract revealed on human skin cells. PLOS ONE. 2021;16(11):e0260545. https://doi.org/10.1371/journal.pone.0260545
83. Sun KY, Sun YT, Li HY, Han DY, Bai YT, Zhao R, et al. Anti-ageing effect of Physalis alkekengi ethyl acetate layer on a D-galactose-induced mouse model through the reduction of cellular senescence and oxidative stress. International Journal of Molecular Sciences. 2020;21(5):1836. https://doi.org/10.3390/ijms21051836
84. He B-W, Wang F-F, Qu L-P. Anti-inflammatory and antioxidant properties of Physalis alkekengi L. extracts in vitro and in vivo: Potential application for skin care. Evidence-Based Complementary and Alternative Medicine. 2022;2022:7579572. https://doi.org/10.1155/2022/7579572
85. Imb M, Véghelyi Z, Maurer M, Kühnel, H. Exploring senolytic and senomorphic properties of medicinal plants for anti-aging therapies. International Journal of Molecular Sciences. 2024;25(19):10419. https://doi.org/10.3390/ijms251910419
86. Ledrhem M, Nakamura M, Obitsu M, Hirae K, Kameyama J, Bouamama H, et al. Essential oils derived from Cistus species activate mitochondria by inducing SIRT1 expression in human keratinocytes, leading to senescence inhibition. Molecules. 2022;27(7):2053. https://doi.org/10.3390/molecules27072053
87. Varela-Eirín M, Carpintero-Fernández P, Sánchez-Temprano A, Varela-Vázquez A, Paíno CL, Casado-Díaz A, et al. Senolytic activity of small molecular polyphenols from olive restores chondrocyte redifferentiation and promotes a pro-regenerative environment in osteoarthritis. Aging. 2020;12(16):15882–15905. https://doi.org/10.18632/aging.103801
88. Huang Y, Peng HY, Wu YF, Deng SC, Ge FH, Ma WB, et al. Rosa roxburghii fruit extracts upregulate telomerase activity and ameliorate cell replicative senescence. Foods. 2024;13(11):1673. https://doi.org/10.3390/foods13111673
89. Cho HM, Lee YR, Lee BW, Zhang M, Ryu B, Nghiem DT, et al. Phenolic constituents of the roots of Rhamnoneuron balansae with senolytic activity. Journal of Natural Products. 2020;83(12):3661–3670. https://doi.org/10.1021/acs.jnatprod.0c00885
90. Matacchione G, Borgonetti V, Ramini D, Silvestrini A, Ojetti M, Galeotti N, et al. Zingiber officinale Roscoe rhizome extract exerts senomorphic and anti-inflammatory activities on human endothelial cells. Biology. 2023;12(3):438. https://doi.org/10.3390/biology12030438
91. Ou ZQ, Rades T, McDowell, A. Anti-ageing effects of Sonchus oleraceus L. (puha) leaf extracts on H2O2-induced cell senescence. Molecules. 2015;20(3):4548–4564. https://doi.org/10.3390/molecules20034548
92. Cai, X., Zhang, Y., Zhu, W., & Gu, N. (2025). Kaempferol inhibits atherosclerotic plaque development via dual-targeting of p53-p21-p16 senescence pathway and Nrf2/HO-1/NQO1 antioxidant mechanism: Insights from combined in vivo and in vitro research. *International Immunopharmacology*, *166*, Article 115587. https://doi.org/10.1016/j.intimp.2025.115587
93. Lee YR, Cho HM, Park EJ, Zhang M, Doan TP, Lee BW, et al. Metabolite profiling of rambutan (Nephelium lappaceum L.) seeds using UPLC-qTOF-MS/MS and senomorphic effects in aged human dermal fibroblasts. Nutrients. 2020;12(5):1430. https://doi.org/10.3390/nu12051430\
94. Bai L, Shi GY, Yang YJ, Chen W, Zhang LF, Qin, et al. Rehmannia glutinosa exhibits anti-aging effect through maintaining the quiescence and decreasing the senescence of hematopoietic stem cells. Animal Models and Experimental Medicine. 2018;1(3):194–202. https://doi.org/10.1002/ame2.12034
95. Xiang M, Liu J, Ma K, Sha Y, Zhan Y, Zhang W, et al. The mechanism of Qijing Mingmu decoction on cellular senescence of conjunctivochalasis. BMC Complementary Medicine and Therapies. 2023;23:383. https://doi.org/10.1186/s12906-023-04138-x
96. Wang JQ, Liu WW, Huang YY, Wang GW, Guo XB, Shi DL, et al. A senomorphlytic three-drug combination discovered in Salsola collina for delaying aging phenotypes and extending healthspan. Advanced Science. 2024;11:e2401862. https://doi.org/10.1002/advs.202401862
97. Wu JC, Bai QZ, Chen JH, Yang ZB, Zhu XY. Systemic analyses of anti-cell-senescence active compounds in Camellia Sect. Chrysantha Chang and their mechanisms. Plants. 2024;13(15):2139. https://doi.org/10.3390/plants13152139
98. Hirose M, Watanabe S, Fujita Y, Ohta S, Sugimoto, M. Senolytic potential of shea-derived amyrins in senescent fibroblasts. Bioscience, Biotechnology, and Biochemistry. 2025. https://doi.org/10.1093/bbb/zbaf153
99. Vo PH, Do NM, Nguyen ST. Primary evaluation the effects of Boesenbergia pandurata ethanol extract on etoposide-induced senescence in fibroblasts. Biomedical Research and Therapy. 2024;11(11):6869–6882. https://doi.org/10.15419/bmrat.v11i11.933

100. Kim EJ, Woo J, Shin S, Choi H, Kim Y, Kim J, et al. A focused natural compound screen reveals senolytic and senostatic effects of Isatis tinctoria. Animal Cells and Systems. 2022;26(6):310–317. https://doi.org/10.1080/19768354.2022.2143895
101. Momtaz S, Baeeri M, Rahimifard M, Haghi-Aminjan H, Hassani S, Abdollahi, et al. Manipulation of molecular pathways and senescence hallmarks by natural compounds in fibroblast cells. Journal of Cellular Biochemistry. 2019;120(5):6209–6222. https://doi.org/10.1002/jcb.27909
102. Doan TP, Zhang M, An JP, Ponce-Zea JE, Mai VH, Ryu B, et al. Metabolite profiling of Allium hookeri leaves using UHPLC-qTOF-MS/MS and the senomorphic activity of phenolamides. Nutrients. 2023;15(24):5109. https://doi.org/10.3390/nu15245109
103. Xu W, Li X-J, Zhong Y-S, He J-Q, Xie W, Kang Y-K, et al. Structural characterizations and antiaging activities of hydrolyzed low-molecular-weight polysaccharides from Polygoni Multiflori Radix Praeparata. Carbohydrate Polymers. 2025;356:123381. https://doi.org/10.1016/j.carbpol.2025.123381
104. Watanabe T, Yazaki M, Yazaki T, Furukawa M, Izumo, N. Senotherapeutic effect of Agrimona pilosa Ledeb. in targeting senescent cells in naturally aged mice. Food Bioscience. 2024;59:103903. https://doi.org/10.1016/j.fbio.2024.103903
105. Rivas-Chacón LDM, Yanes-Díaz J, de Lucas B, Riestra-Ayora JI, Madrid-García R, Sanz-Fernández R, et al. Cocoa polyphenol extract inhibits cellular senescence via modulation of SIRT1 and SIRT3 in auditory cells. Nutrients. 2023;15(3):544. https://doi.org/10.3390/nu15030544
106. Lämmermann I, Terlecki-Zaniewicz L, Weinmüllner R, Schosserer M, Dellago H, de Matos Branco AD, et al. Blocking negative effects of senescence in human skin fibroblasts with a plant extract. NPJ Aging and Mechanisms of Disease. 2018;4:4. https://doi.org/10.1038/s41514-018-0023-5
107. Zumerle S, Sarill M, Saponaro M, Colucci M, Contu L, Lazzarini E, et al. Targeting senescence induced by age or chemotherapy with a polyphenol-rich natural extract improves longevity and healthspan in mice. Nature Aging. 2024;4:1481–1497. https://doi.org/10.1038/s43587-024-00663-7
108. Wang YY, Chang JH, Liu XG, Zhang X, Zhang SP, Zhang X, et al. Discovery of piperlongumine as a potential novel lead for the development of senolytic agents. Aging. 2016;8(11):2915–2926. https://doi.org/10.18632/aging.101100
109. Moaddel R, Rossi M, Rodriguez S, Munk R,Khadeer M, Abdelmohsen K, et al. Identification of gingerenone A as a novel senolytic compound. PLOS ONE. 2022;17(3):e0266135. https://doi.org/10.1371/journal.pone.0266135
110. Moaddel, R.; Sanehira, C.; Keyes, G.; Cui, C.-Y.; Ahmadkhaniha, R.; Candia, J.; Price, N.L.; Eckroth, S.; Middleton, B.; Khadeer, M.; et al. Senolytic effects of a modified Gingerenone A. *npj Aging* 2025, *11*, 45. https://doi.org/10.1038/s41514-025-00230-3
111. Zhang Y, Gao D, Yuan Y, Zheng R, Sun M, Jia S, et al. Cycloastragenol: A novel senolytic agent that induces senescent cell apoptosis and restores physical function in TBI-aged mice. International Journal of Molecular Sciences. 2023;24(7):6554. https://doi.org/10.3390/ijms24076554
112. Xia M-L, Xie X-H, Ding J-H, Du R-H, Hu, G. Astragaloside IV inhibits astrocyte senescence: Implication in Parkinson's disease. Journal of Neuroinflammation. 2020;17:106. https://doi.org/10.1186/s12974-020-01791-8
113. Jiang BR, Zhang HW, Xu QX, Jiang ZR, He RK, Fu Q, et al. Pyrroloquinoline quinone is an effective senomorphic agent to target the pro-inflammatory phenotype of senescent cells. Aging Cell. 2025;24:e70138. https://doi.org/10.1111/acel.70138
114. Liu Y, Ye J, Fan Z, Wu X, Zhang Y, Yang R, et al. Ginkgetin alleviates inflammation and senescence by targeting STING. Advanced Science. 2025;12:2407222. https://doi.org/10.1002/advs.202407222
115. Guerrero A, Herranz N, Sun B, Wagner V, Gallage S, Guiho R, et al. Cardiac glycosides are broad-spectrum senolytics. Nature Metabolism. 2019;1(11):1074–1088. https://doi.org/10.1038/s42255-019-0122-z
116. Ma XY, Han L, Xu MR, Feng YL, Liu CS, Zhao YD, et al. Eleutheroside E ameliorates D-Gal-induced senescence in human skin fibroblasts through PI3K/AKT signaling. Current Issues in Molecular Biology. 2025;47(11):895. https://doi.org/10.3390/cimb47110895
117. Lin Q, Fan WJ, Chen ZY, Chen B, Zhang CF. Hesperetin alleviates bleomycin-induced pulmonary fibrosis by modulating cellular senescence and promoting impaired autophagy in a CISD2-dependent manner. The FASEB Journal. 2025;39:e70408. https://doi.org/10.1096/fj.202502311R

118. Sax SL,Centamo ML, Centofanti F, Rizzacasa B, Cox S, Cox C, et al. The senolytic effect of indole-3-carbinol (I3C) on mouse embryonic (MEF) and human fibroblast cell lines. International Journal of Molecular Sciences. 2024;25(21):11652. https://doi.org/10.3390/ijms252111652
119. Borgonetti V, Galeotti, N. Rosmarinic acid reduces microglia senescence: A novel therapeutic approach for the management of neuropathic pain symptoms. Biomedicines. 2022;10(7):1468. https://doi.org/10.3390/biomedicines10071468
120. Tien XY, Lee YK, Wong PF, Khor YS, Murugan DD, Abdullah, et al. Anti-senescence effects of 4-methoxychalcone and 4-bromo-4'-methoxychalcone on human endothelial cells. Drug Discoveries & Therapeutics. 2024;18(3):199–206. https://doi.org/10.5582/ddt.2024.01034
121. Wang C, Hu X, Song T, Hu F, Du L, Yan C, et al. Magnolin mitigates skin ageing through the CXCL10/p38 signalling pathway. Journal of Cellular and Molecular Medicine. 2025;29:e70507. https://doi.org/10.1111/jcmm.70507
122. Gu, Y., Zhu, Q., Qin, Y., & Han, J. (2025). Isovitexin attenuates oxidative stress-induced skin photoaging by suppressing cellular senescence: an insight provided by transcriptomic and metabolomic. *Journal of Agriculture and Food Research*, 23, 102172. https://doi.org/10.1016/j.jafr.2025.102172
123. Zhu Y, Zhang Y, Zhang Q, Song P, Zhang J, Ma A, Wang C, Gao P, Yang T, Zhou L, Shi Q, Wong YK, Luo Y, Tang H, Wang J. Caffeic Acid Acts as a Potent Senomorphic and Alleviates Inflammation and Lung Fibrosis by Covalently Targeting Annexin A5 Protein in Mice. Exploration (Beijing). 2025 Dec 12;5(6). https://doi.org/10.1002/exp.20240069
124. Yang JJ, Zhao SL, Zhang X, Ma YG, Jiang JG. Polygonatum kingianum saponins delay cellular senescence through SASP down-regulation and prolong the healthy lifespan of Caenorhabditis elegans by activating sir-2.1/autophagy. Industrial Crops and Products. 2023;201:116903. https://doi.org/10.1016/j.indcrop.2023.116903
125. Wang, S.; Li, H.; Zhao, F.; Gao, J.; Huang, S.; Liu, X.; Ma, B. Production and Optimization of Anti-Aging Peptides from *Pleurotus eryngii* Mushroom Feet: Mechanistic Insights via Integrated Transcriptomics and Metabolomics. *Foods* 2025, *14*, 3977. https://doi.org/10.3390/foods14223977
126. Lan, W.; Xiao, X.; Nian, J.; Wang, Z.; Zhang, X.; Wu, Y.; Zhang, D.; Chen, J.; Bao, W.; Li, C.; et al. Senolytics Enhance the Longevity of *Caenorhabditis elegans* by Altering Betaine Metabolism. Journals of Gerontology, Series A: Biological Sciences and Medical Sciences. 2024, *79*, glae221. https://doi.org/10.1093/gerona/glae221
127. Wu, J.; Dongzhu, N.; Zhao, C.; Liang, J.; Wang, N.; Suonan, D.; Sun, S. Multi-Target Botanical Complex Attenuates Cellular Senescence via Bidirectional P21/P53/SIRT1 Regulation: Dual Model Validation. International Journal of Molecular Sciences. 2025, *26*, 11394. https://doi.org/10.3390/ijms262311394
128. Deepika, Maurya PK. Health benefits of quercetin in age-related diseases. Molecules. 2022;27(8):2498. https://doi.org/10.3390/molecules27082498
129. Saccon TD, Nagpal R, Yadav H, Cavalcante MB, Nunes ADC, Schneider A, et al. Senolytic combination of dasatinib and quercetin alleviates intestinal senescence and inflammation and modulates the gut microbiome in aged mice. Journals of Gerontology Series A. 2021;76(11):1895–1905. https://doi.org/10.1093/gerona/glab002
130. Hense JD, Garcia DN, Isola JV, Alvarado-Rincón JA, Zanini BM, Prosczek JB, et al. Senolytic treatment reverses obesity-mediated senescent cell accumulation in the ovary. GeroScience. 2022;44(4):1747–1759. https://doi.org/10.1007/s11357-022-00573-9
131. Razazian M, Bahiraii S, Sohail A, Mandl M, Jannat I, Beilhack G, et al. Fisetin ameliorates vascular smooth muscle cell calcification via DUSP1-dependent p38 MAPK inhibition. Aging. 2025;17:885–907. https://doi.org/10.18632/aging.206233
132. Walle T. Bioavailability of resveratrol. Annals of the New York Academy of Sciences. 2011;1215(1):9–15. https://doi.org/10.1111/j.1749-6632.2010.05842.x
133. Barra V, Chiavetta RF, Titoli S, Provenzano IM, Carollo PS, Di Leonardo A. Specific irreversible cell-cycle arrest and depletion of cancer cells obtained by combining curcumin and the flavonoids quercetin and fisetin. Genes. 2022;13(7):1125. https://doi.org/10.3390/genes13071125
134. Shang R, Yang J, Hu W, Hu J, Tang Y, Wang Y, et al. Targeted elimination of senescent cells by procyanidin C1 improves diabetic wound healing and restores skin quality. Free Radical Biology and Medicine. 2025. https://doi.org/10.1016/j.freeradbiomed.2025.08.028
135. Hou J, Yun Y, Jeon B, Baek J, Kim S. Ginsenoside F1-mediated telomere preservation delays cellular senescence. International Journal of Molecular Sciences. 2023;24(18):14241. https://doi.org/10.3390/ijms241814241
136. Huang CY, Chen SH, Lin T, Liao YW, Chang YC, Chen CC, et al. Resveratrol attenuates advanced glycation end product-induced senescence and inflammation in human gingival fibroblasts. Journal of Dental Sciences. 2024;19(1):580–586. https://doi.org/10.1016/j.jds.2023.10.016

137. Dabbousy, R., Rima, M., Roufayel, R., Rahal, M., Legros, C., Sabatier, J.-M., Fajloun, Z. Plant Metabolomics: The Future of Anticancer Drug Discovery. *Pharmaceuticals* 2024, *17*, 1307. https://doi.org/10.3390/ph17101307
138. Aladejana EB. Biological properties of polyherbal formulations: a review of their antimicrobial, anti-inflammatory, antioxidant, and toxicological activities. Pharmacognosy Journal. 2023;15(6):933–963. https://doi.org/10.5530/pj.2023.15.178
139. Chaib S, Tchkonia T, Kirkland JL. Cellular senescence and senolytics: the path to the clinic. Nature Medicine. 2022;28(8):1556–1568. https://doi.org/10.1038/s41591-022-01923-y
140. Dou Z, Ghosh K, Vizioli MG, Zhu J, Sen P, Wangensteen KJ, et al. Cytoplasmic chromatin triggers inflammation in senescence and cancer. Nature. 2017;550(7676):402–406. https://doi.org/10.1038/nature24050
141. Ballini, A., Barile, S.N., De Rosa, A., Bizzoca, M.E., Boccellino, M., Scacco, S., Cantore, S., Lo Muzio, L., Lasorsa, F.M., Arrigoni, R. The Polyphenol-Microbiota Axis: Molecular Mechanisms, Metabolic Pathways, and Therapeutic Perspectives in Human Health. Journal of Personalized Medicine. 2026, *16*, 289. https://doi.org/10.3390/jpm16030289
142. Gaudêncio SP, Bayram E, Lukic Bilela L, Cueto M, Díaz-Marrero AR, Haznedaroglu BZ, et al. Advanced methods for natural product drug discovery. Marine Drugs. 2023;21(8):460. https://doi.org/10.3390/md21080460
143. Bucar F, Grillitsch A, Hamburger M. Phytochemistry meets biological activity: how to close the gap? Phytochemistry Reviews. 2013;12(4):533–557. https://doi.org/10.1007/s11101-013-9300-5
144. Wishart DS, Guo A, Oler E, Wang F, Anjum A, Peters H, et al. HMDB 5.0: the Human Metabolome Database for 2022. Nucleic Acids Research. 2022;50(D1):D622–D631. https://doi.org/10.1093/nar/gkab1062
145. Chacón E, Grünwaldt G, Marmisolle I, Martínez J, Quijano C. Redox metabolism in cell senescence: focusing on contributions from the metabolomic field. Frontiers in Molecular Biosciences. 2025;12:1754469. https://doi.org/10.3389/fmolb.2025.1754469
146. Parasuraman S, Thing GS, Dhanaraj SA. Polyherbal formulation: concept of Ayurveda. Pharmacognosy Reviews. 2014;8(16):73–80. https://doi.org/10.4103/0973-7847.134229
147. Joma N, Bielawski P-B, Saini A, Kakkar A, Maysinger D. Nanocarriers for natural polyphenol senotherapeutics. Aging Cell. 2024;23:e14178. https://doi.org/10.1111/acel.14178
148. Lewinska A, Przybylski P, Adamczyk-Grochala J, Bloniarz D, Litwinienko G, Wnuk M. Senolysis-based elimination of chemotherapy-induced senescent breast cancer cells by quercetin derivative with blocked hydroxy groups. Cancers. 2022;14(3):605. https://doi.org/10.3390/cancers14030605
149. Sharma R. Emerging interrelationship between the gut microbiome and cellular senescence in the context of aging and disease: perspectives and therapeutic opportunities. Probiotics and Antimicrobial Proteins. 2022;14(4):648–663. https://doi.org/10.1007/s12602-021-09903-3
150. Stasiłowicz-Krzemień A, Rosiak N, Racaniello GF, Denora N, Cielecka-Piontek J. Effective and stable senomorphic apigenin delivery system obtained by supercritical carbon dioxide processing. International Journal of Molecular Sciences. 2025;26(17):8126. https://doi.org/10.3390/ijms26178126
151. Yu L, Liu P. cGAS/STING signalling pathway in senescence and oncogenesis. Seminar Cancer Biology. 2024;106-107:87-102. https://doi.org/10.1016/j.semcancer.2024.08.007
